# The Resolution Axis Method (RAM) for lengthening of the femur with or without associated frontal plane deformity (a new method)

**DOI:** 10.1007/s11751-018-0312-3

**Published:** 2018-05-24

**Authors:** Sherif Galal

**Affiliations:** Department of Orthopaedic Surgery, Faculty of Medicine, Cairo University, Cairo, 11559 Egypt

**Keywords:** Deformity, Femur, Lengthening, Planning

## Abstract

**Introduction:**

Femoral lengthening with or along intramedullary (IM) nails will occur along the axis of the nail coincident with the anatomical axis of the bone. In the femur particularly, such lengthening often creates lateral mechanical axis deviation as the knee is driven medially. In cases where shortening is associated with frontal plane deformity the surgeon needs to correct the deformity intra-operatively, however, subsequent lengthening along the anatomical axis will create deformity. Thus, planning for lengthening of the femur with or along IM nails, whether shortening is associated with frontal plane deformity or not, requires a completely different planning strategy. The author questioned if a *resolution* anatomical axis can be identified and used for planning when lengthening the femur along or with IM nails while still applying the same classic CORA deformity analysis method.

**Methods:**

In a prospective study, the author included eight patients who needed femoral lengthening, five with associated frontal plane deformity and three without. The author identified a method to determine the trajectory of the nail in the lower femoral segment. It was done by calculating the angle enclosed between this *resolution* anatomical axis and the mechanical axis, also known as the anatomical-mechanical angle.

**Results:**

This new method has proven to be effective in achieving normal alignment after lengthening is completed.

**Conclusion:**

The Resolution Axis Method is a new and alternative method providing a solution for planning when lengthening the femur along the anatomical axis using an IM nail, whether a deformity is present or not.

**Electronic supplementary material:**

The online version of this article (10.1007/s11751-018-0312-3) contains supplementary material, which is available to authorized users.

## Introduction

Compared to external fixation, where deformity correction is done gradually and lengthening occurs along the mechanical axis, the deformity correction in lengthening over nails (LON) and, more recently, implantable motorized lengthening nails is done acutely, and lengthening occurs along the nail axis coincident with the anatomical axis of the bone. In the femur, this results in frontal plane medial translation of the knee creating lateral mechanical axis deviation [1, 2].

When planning for such lengthening of the femur with intramedullary (IM) nail, whether shortening is associated with frontal plane deformity or not, a different planning strategy is required. To finally align the mechanical axis and correct joint orientation angles when lengthening is completed, both the original deformity and that created by lengthening along the anatomical axis must be considered [1].

The classic center of rotation of angulation (CORA) deformity analysis method using anatomical axis planning dictates that a deformity should be represented by two or more anatomical axes representing each deformed segment, the intersection point of which is the apex of deformity, a specific CORA [3]. Around this point, osteotomy and correction is planned and occurs following osteotomy rules. Where lengthening in addition to deformity correction is required, this method cannot be used. It does not take into consideration a secondary deformity occurring when lengthening the femur along the anatomical axis or the resultant effect on the mechanical axis.

The author questioned if a *resolution* anatomical axis can be identified and used for planning when lengthening the femur with or along IM nails while still applying the same classic CORA deformity analysis method.

## Method

In a prospective study, the author included eight patients who needed femoral lengthening (five with associated frontal plane deformity and three without). Patients were either skeletally mature or had a premature closure of the lower femoral physis to make them candidates for retrograde IM nail insertion. There were five males and three females; average age was 15.2 years (12–20 years). The five patients who had combined shortening and deformity; the etiology was post-traumatic physeal closure of the lower femoral physis. The three patients who had shortening only had overgrowth of the contralateral femur; two of them following a childhood fracture and the other had local gigantism. The lengthening needed was 5 cm on average (4–8 cm). Deformity was evaluated using the anatomical lateral distal femoral angle (aLDFA) and the mechanical axis deviation (MAD) measured in centimeters. The aLDFA of the healthy contralateral side was used as a reference to the normal value. Mechanical axis deviation of zero was considered the normal, while medial deviation (varus deformity) was given a plus (+) sign and lateral deviation (valgus deformity) was given a negative (−) sign (e.g., + 3 MAD would be medial mechanical axis deviation by 3 cm, − 2 MAD would be lateral mechanical axis deviation by 2 cm).

All patients were lengthened using the lengthening over nail (LON) technique described by Paley et al. [4]; intra-operative deformity correction was done by retrograde nails using fixator-assisted nailing (FAN) described by Paley et al. [5]. A latency period of 7 days was used, after which lengthening was done at a rate of 1 mm/day performed as 0.25 mm increments 4 times/day. Patients were followed up, by radiographs of the femur, biweekly until desired length was achieved, then monthly until complete consolidation. Long leg films were obtained at the end of lengthening to confirm achieving the desired length, then at final follow-up. After desired length was achieved, the fixator was left for 1 more month to ensure improved regenerate quality before removing the fixator and locking the IM nail. Physical therapy was started after wound healing, full weight bearing was only allowed when the regenerate showed three continuous cortices, 2 mm thick on two orthogonal radiographs. Follow-up was on average 12 months (10–16 months).

### Trigonometric origin of the equation

**Fig. 1:**
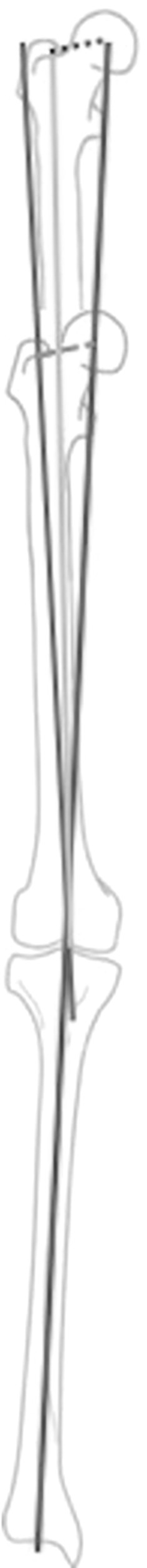
Center of femoral head (CH) must be located on the upward extension of the mechanical axis of the femur by the end of lengthening to avoid mechanical axis deviation. Red line: mechanical axis, blue line: classic anatomical axis, yellow line: resolution anatomical axis, dashed green line: femoral neck length at start of lengthening, dotted purple line: femoral neck length at the end of lengthening (note that the dashed green line and the dotted purple line are of equal length as both represent the femoral neck length which does not change during lengthening)

The center of femoral head (CH) must be located along the upward extension of the normal mechanical axis of the lower femoral segment at the end of lengthening (Fig. 1, red line). This is to ensure there would be no residual deformity in the final (post-lengthening) position.

The femoral neck length does not change during lengthening; piriform fossa (the upper element of the anatomical axis) would therefore assume a more medial position relative to its starting position. The CH–piriformis fossa distance (Fig. 1, dashed green line) spans the distance between the anatomical and the mechanical axis (the angle enclosed between the two axes is 7° on average) at the start of lengthening. Using trigonometry, the distance between two lines enclosing an angle increases as you move away from the apex of that angle. At the end of lengthening, the CH–piriformis fossa distance (Fig. 1, dotted purple line) will therefore not match the distance between the upward extension of the “original” anatomical axis (Fig. 1, blue line) and the mechanical axis. Note that the dashed green line and the dotted purple line are of equal length as both represent the CH–piriformis fossa distance which does not change during lengthening.

This is opposite to what happens to the center of the femoral head (CH) which moves to a more lateral position (causing lateral mechanical axis deviation) when lengthening is done along the anatomical axis.

Thus, we can conclude that in order to end up with a normal alignment after lengthening, the femoral anatomical-mechanical angle (AMA) in the post-lengthening position will be less than that in the pre-lengthening position and that the more you lengthen, the less the femoral anatomical-mechanical angle (AMA) would be.

**Fig. 2:**
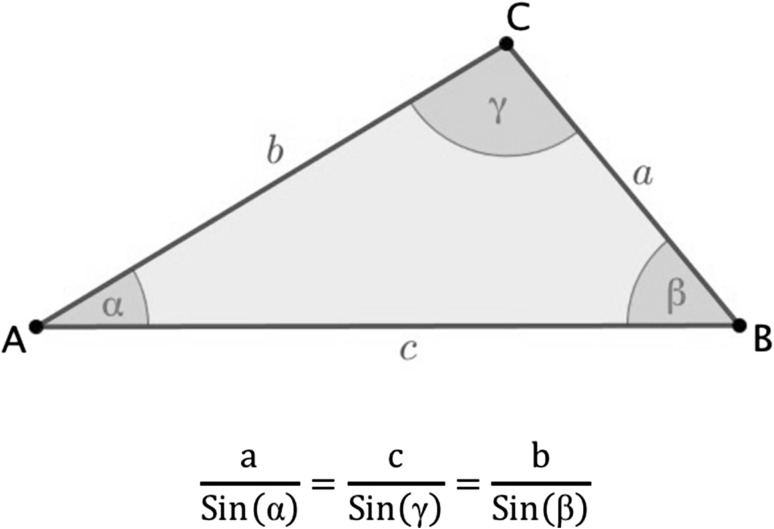
The Trigonometric “Law of Sines”

The Trigonometric “Law of Sines” states that in any triangle (Fig. 2) where a, b, and c are the lengths of the sides of a triangle, and α, γ, and β are the opposite angles, the following is true:

$$\frac{a}{{\text{Sin} \left( \alpha \right)}} = \frac{c}{{\text{Sin} \left( \gamma \right)}} = \frac{b}{{\text{Sin} \left( \beta \right)}}$$

**Fig. 3:**
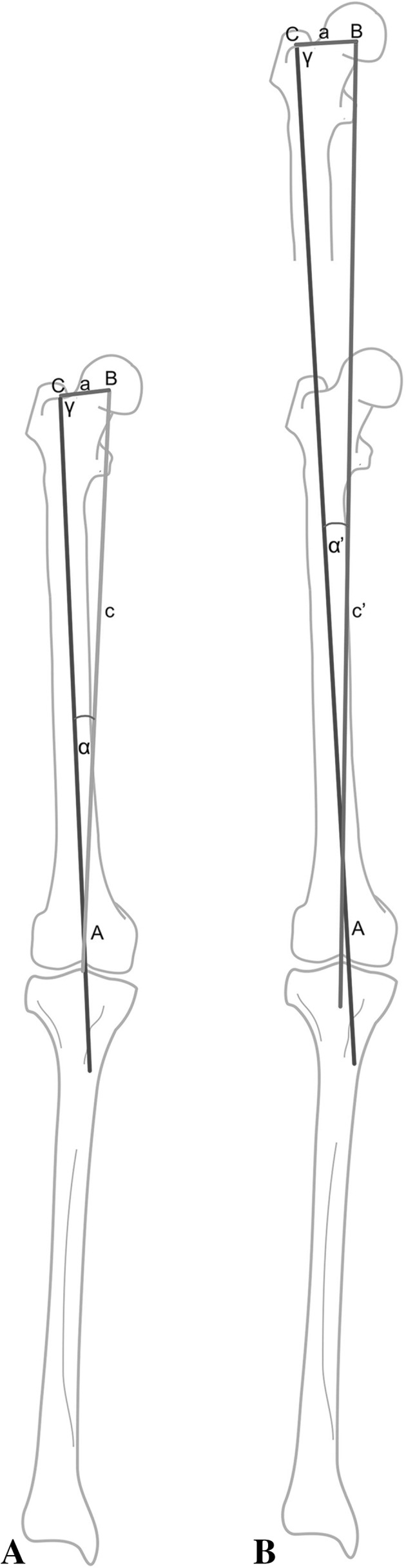
**a** Pre-lengthening condition, we can draw a triangle ABC, Point A represents the nail entry point at the center of the knee. Point B represents the center of the femoral head (CH). Point C represents the piriformis fossa. AB line represents the femoral mechanical axis and we will refer to its length as “c”. BC line is the femoral neck and we will refer to its length as “a”. We will refer to the femoral anatomic-mechanical angle (AMA) as alpha “α” (the angle between AB and AC lines). We will refer to the angle between the mid-diaphyseal (anatomical axis) line of the proximal femoral fragment (AC line) and the line joining the center of the femoral head to the piriformis fossa (BC line) as gamma “γ”. **b** Post-lengthening condition, we will use A, B, C to refer to the same points as before. We will refer to the (new) length of the femoral mechanical axis as “c′”, the femoral neck length will remain (unchanged) as “a”. We will refer to the (*new*) anatomical-mechanical angle (FMA) as alpha′ “α′”, angle “γ” will remain (unchanged) because the center of femoral head (CH), piriformis and the proximal femoral fragment retain their anatomical relationship

In the pre-lengthening condition, we can draw a triangle ABC (Fig. 3a) where Point A represents the nail entry point at the center of the knee. Point B represents the center of the femoral head. Point C represents the piriformis fossa.

AB line represents the femoral mechanical axis, and we will refer to its length as “c”. BC line is the femoral neck, and we will refer to its length as “a”. We will refer to the normal femoral anatomical-mechanical angle (AMA) as alpha “α” (the angle between AB and AC lines). We will refer to the angle between the mid-diaphyseal (anatomical axis) line of the proximal femoral fragment (AC line) and the line joining the center of the femoral head to the piriformis fossa (BC line) as gamma “γ”.

In this triangle, we only need to measure the distance “c” and angle α (normally 7° on average).

In the post-lengthening condition (Fig. 3b), we will use A, B, C to refer to the same points as before. We will refer to the new length of the femoral mechanical axis as “c′”, the femoral neck length will remain unchanged as “a”. We will refer to the *new* femoral anatomical-mechanical angle (AMA) as alpha′ “α′”, angle “γ” will remain unchanged because the center of femoral head (CH), piriformis and the proximal femoral fragment retain their anatomical relationship.

In this triangle, we only need to measure the distance “c′” (c′ = c + amount of lengthening).

For pre-lengthening triangle (Fig. 3a):

1 $$\frac{a}{{\text{Sin} \left( \alpha \right)}} = \frac{c}{{\text{Sin} \left( \gamma \right)}} \to a \times \text{Sin} \left( \gamma \right) = c \times \text{Sin} \left( \alpha \right)$$

For post-lengthening triangle (Fig. 3b):

2 $$\frac{a}{{\text{Sin} \left( {\alpha '} \right)}} = \frac{c^{\prime}}{{\text{Sin} \left( \gamma \right)}} \to a \times \text{Sin} \left( \gamma \right) = c^{\prime} \times \text{Sin} \left( {\alpha^{\prime}} \right)$$

Hence, the left-hand side of Eqs. 1 and 2 is the same [a $\times$ $\text{Sin} \left( \gamma \right)$]; then, the right-hand side of both equations would be equal

$${\text{Thus}}, \, c^{\prime} \times \text{Sin} \left( {\alpha^{\prime}} \right) = c \times \text{Sin} \left( \alpha \right) \to \text{Sin} \left( {\alpha^{\prime}} \right) = \frac{c}{c^{\prime}} \times \text{Sin} \left( \alpha \right)$$

In this equation, only (sin α′) is unknown and by using the inverse trigonometric function of “Arcsin” we can obtain the value of this angle which is the value for the *new* femoral anatomical-mechanical angle (AMA).

### Planning objective

When planning for cases where lengthening is required in addition to femoral deformity correction, by applying the classic CORA deformity analysis method using anatomical axis planning, the anatomical axis of the upper (shaft) part of the femur would be represented by a mid-diaphyseal line.

For the lower (metaphysis) part of the femur instead of representing this segment by the classic anatomical axis which is drawn referenced to the mechanical axis of the lower femur by 7º (normal femoral anatomical-mechanical angle is 7º on average), the author proposes a *resolution* anatomical axis (Fig. 1, yellow line) drawn referenced to the mechanical axis by a *new* femoral anatomical-mechanical angle (AMA) which is smaller than normal. The value of this angle would be derived from the following equation;

$$\text{Sin} \left( {\alpha '} \right) = \frac{c}{c'} \times \text{Sin} \left( \alpha \right)$$

where (α′) is the *new* femoral anatomical-mechanical angle (AMA) which is enclosed between the *resolution* anatomical axis and the mechanical axis, (α) is the normal femoral anatomical-mechanical angle (AMA) which is 7° on average, (c) is the pre-lengthening femoral length (distance between knee center and femoral head center), and (c′) is the final femoral length after lengthening = (c + needed lengthening).

In this equation, only (Sin α′) would be unknown, and by using the inverse trigonometric function of “Arcsin”, we can get the value of the angle (α′) and, hence, the value of the *new* femoral anatomical-mechanical angle (AMA).

For example, if you have a femur which is 30 cm long and you want to lengthen it by 5 cm and the femoral anatomical-mechanical angle (AMA) in that patient is 7°, applying these variables to the equation will result in a *new* femoral anatomical-mechanical angle (AMA) value of 6°, so you should draw the *resolution* anatomical axis of the lower femur referenced to the mechanical axis by 6° instead of 7°.

$$Sin \, \alpha ^{\prime} = \frac{30}{35} \times {\text{Sin}}\left( {7^\circ } \right)^\circ = 0.1044\;{\text{Thus}}\,\alpha^{\prime} \approx 6^\circ$$

### Planning method

**Fig. 4:**
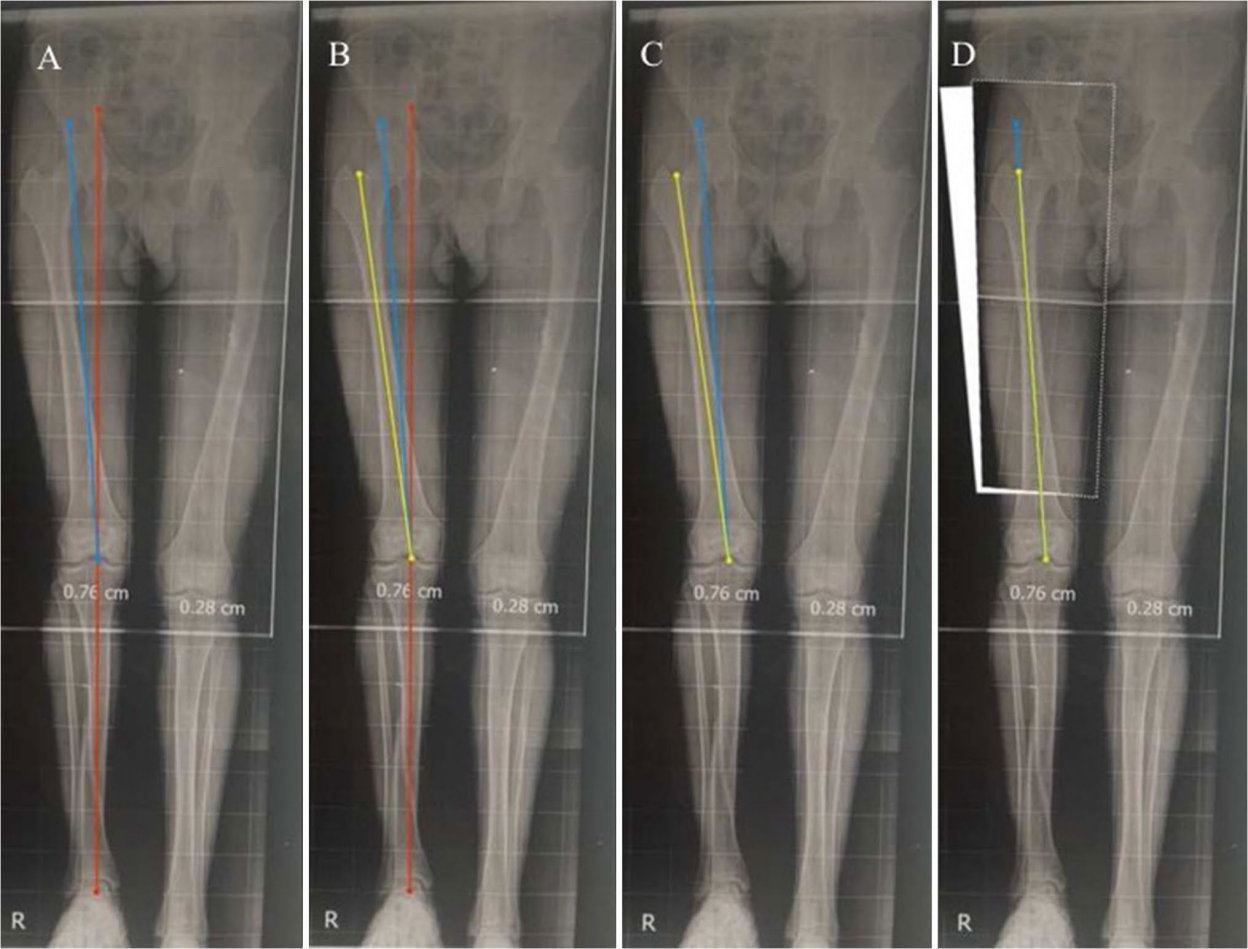
**a** Draw the normal mechanical axis of the femur (red line) by extending that of the tibia up, then draw the “*resolution*” anatomical axis of the lower femoral segment (blue line) referenced to the mechanical axis with an AMA angle as derived from the equation. **b** Draw the anatomical axis line of the upper femoral segment (yellow line) by drawing the mid-diaphyseal line. **c** The point where the 2 axes intersects would be the “resolution” CORA that puts into account both the deformity present and that resulting from lengthening along the anatomical axis of the femur. **d** Plan your osteotomy site so enough bone would be available for stabilization using IMN, determine how much translation needed at the osteotomy site if any

*Step 1* (Fig. 4a)

Draw the normal mechanical axis of the femur (red line) by either

- extending the mechanical axis line of the tibia upward, if it is normal, or
- drawing a line starting from the knee center making a lateral angle, mLDFA, of 87° (population average) or as it measures on the other normal side.

*Step 2* (Fig. 4a)

Draw the *resolution* anatomical axis line of the lower femoral segment (blue line), starting at the knee center and referenced to the mechanical axis (drawn in step 1) by the *new* femoral anatomical-mechanical angle (AMA), the value of which is derived from the equation mentioned before (this would be less than value of *normal* femoral anatomical-mechanical angle of 7°)

Starting point is the center of the knee as this is a *resolution* anatomical axis and is different from the normal anatomical axis starting point at the knee that is 10 ± 5 mm medial to the knee center. For retrograde nailing, the distal metaphysis allows for a reselection of the nail entry point and hence the *resolution* anatomical axis starting point.

*Step 3* (Fig. 4b)

Draw the anatomical axis line of the upper (shaft) femoral segment (yellow line) by drawing the mid-diaphyseal line.

*Step 4* (Fig. 4c)

The point where the anatomical axis of the upper segment (yellow line) intersects the *resolution* anatomical axis of the lower segment (blue line) would be the *resolution* apex of the deformity that takes into account both the deformity present and that resulting from lengthening along the anatomical axis of the femur.

* Step 5* (Fig. 4d)

Plan your osteotomy site so enough bone would be available for stabilization using IM nail, if the osteotomy is done at the level of the apex of the deformity, no translation will be needed, according to osteotomy rule 1 proposed by Paley [3]. But if the osteotomy is done away from the deformity apex, translation would be needed at the osteotomy site, translation will be equal to the distance between the anatomical axis of the upper segment and the *resolution* anatomical axis of the lower segment at the level of the osteotomy according to osteotomy rule 2 proposed by Paley [3].

### Surgical technique

The whole procedure is done as a fixator-assisted nailing (FAN) procedure [5].

**Fig. 5:**
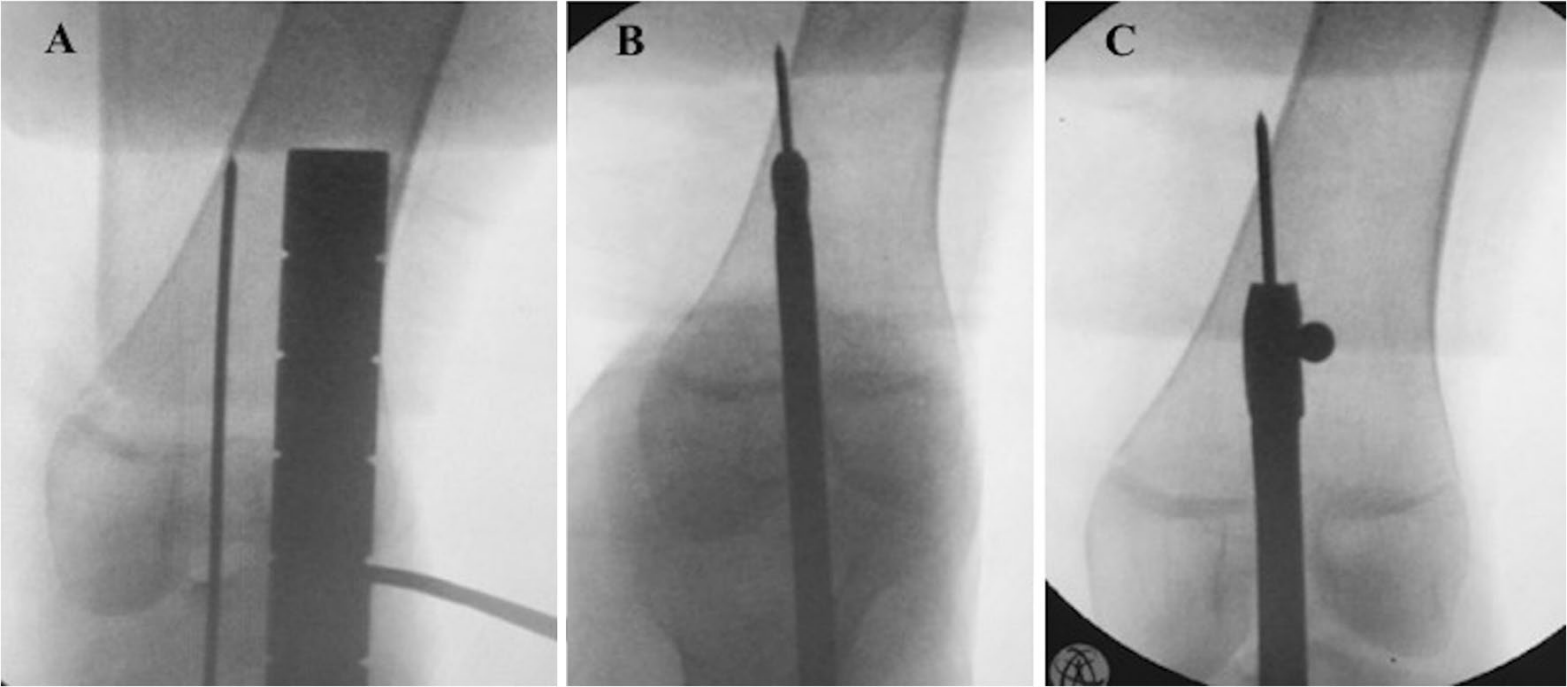
Intra-operative reproduction of the “*resolution*” anatomical axis, **a** guide pin for starter reamer. **b** reaming to prepare the canal for nail passage in the lower fragment. **c** Blocking screw to prevent any nail play in the lower femoral fragment

During pre-operative planning, the surgeon must identify where the *resolution* anatomical axis intersects the cortex proximally and measure the distance from this point to a recognizable radiographic landmark, e.g., knee joint line, so as to easily reproduce this line intra-operatively. This can be accomplished by inserting a guide pin (for starter reamer**)** from the center of the knee adding proximally to the identified point on the cortex. This pin will be the trajectory for the retrograde nail insertion. Figure 5 shows intra-operative images for the case illustrated in Fig. 9.

Canal venting is done by performing multiple drill holes at the planned osteotomy site.

A reamer is introduced along this guide pin in the lower fragment to create the canal for the IM nail but stopping short of the planned osteotomy site. Successive reaming of this segment is done until the planned canal size is reached (Fig. 5b).

With the thickest reamer still inside, blocking screws [6] are inserted along the sides of this canal to prevent any room for nail play after insertion (Fig. 5c).

**Fig. 6:**
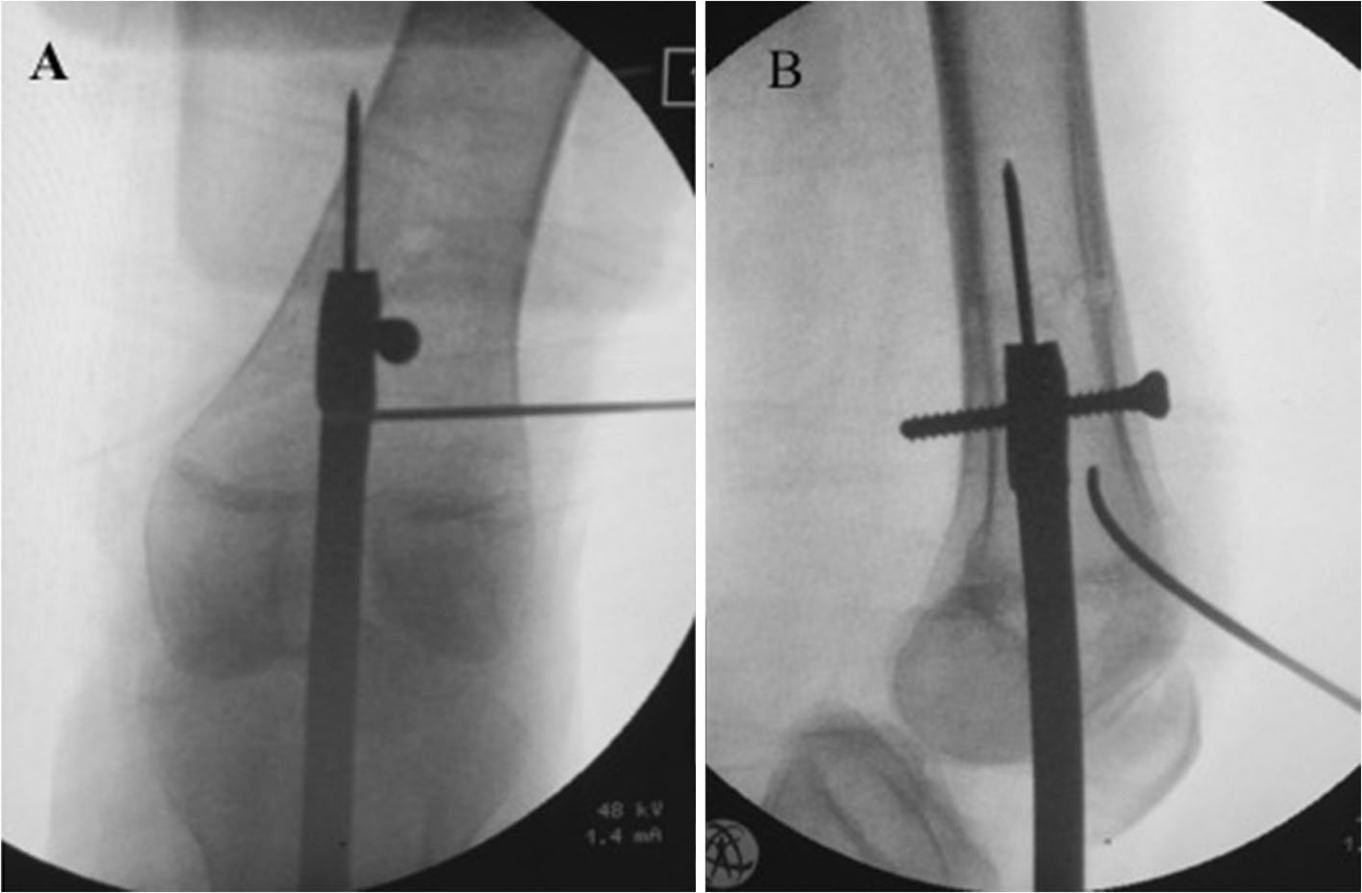
Schanz screws insertion should be perpendicular to the nail trajectory (**a**), and anterior to nail track in the lower femur (**b**)

One Schanz screw, or three in case lengthening will be done by LON, is inserted perpendicular to the guide pin in the frontal plane (Fig. 6a) and anterior to the nail in the sagittal plane, so as to clear the path of the reamers and nail insertion (Fig. 6b).

**Fig. 7:**
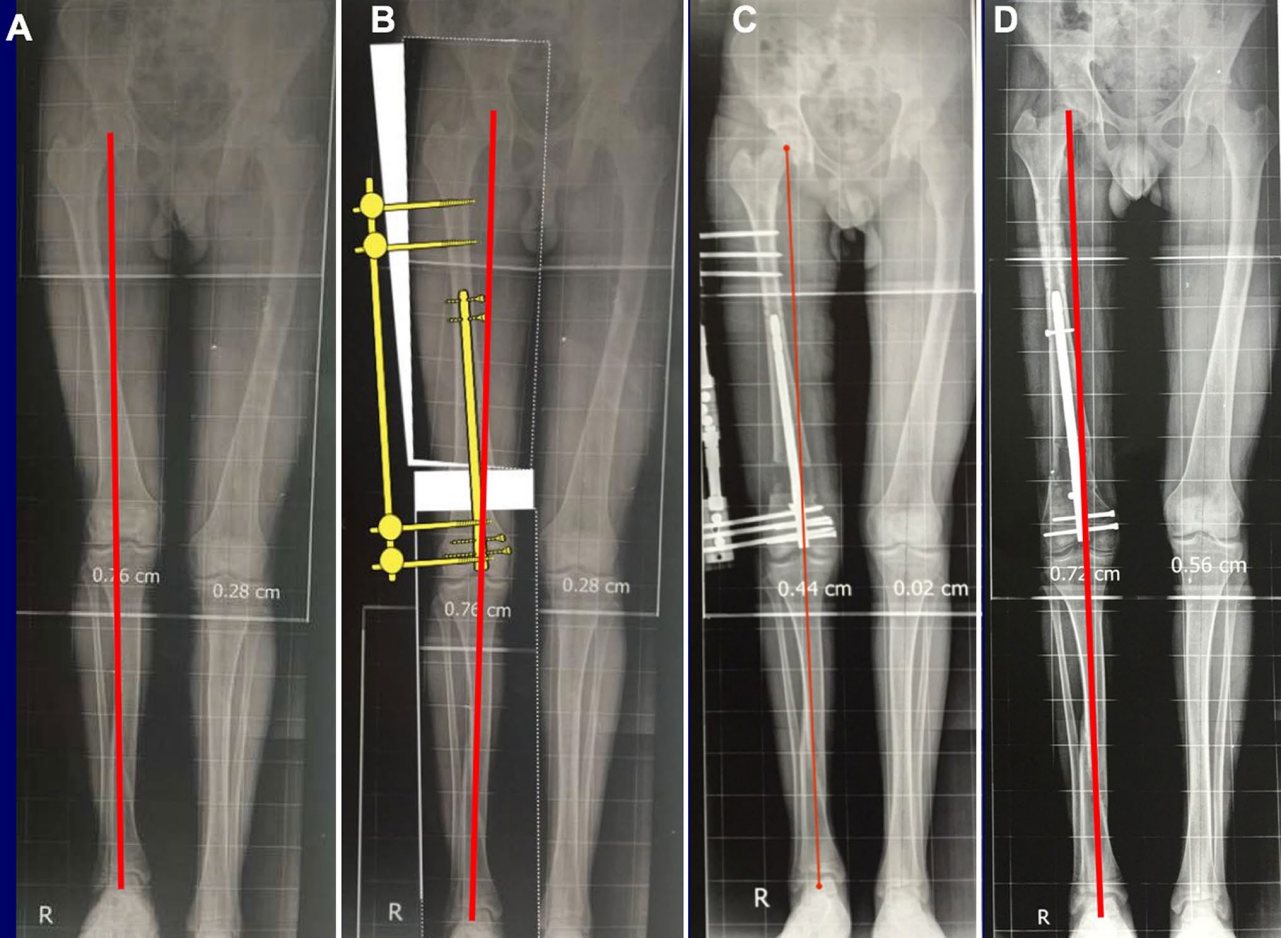
**a** Pre-operative radiograph with a slight lateral MAD, **b** the proposed planning to achieve normal limb alignment after lengthening is completed, **c** the reproduction of the planning and achieving normal alignment at the end of lengthening with no residual MAD, **d** the final radiograph after regenerate consolidation with normal limb alignment and no residual MAD

A second Schanz screw, or three in case lengthening will be done by LON, is inserted proximally well above the nail (Fig. 7c) perpendicular to the shaft and anatomical axis of the upper segment in the frontal plane.

Percutaneous osteotomy is done at the planned site.

If translation at the osteotomy site is needed, it could be done using the proximal and distal Schanz screws as joysticks to manipulate bone fragments or by the help of a small osteotome pushed across the osteotomy site.

Fixator is locked, and limb alignment is checked, e.g., using alignment rod.

Then, a blunt tipped intramedullary guide wire for the nail reamers is inserted along the pre-prepared canal of the lower fragment and along the medullary canal of the upper segment. Reaming is then continued until the planned canal diameter is reached. A retrograde IM nail is then inserted.

The final alignment is checked again; more blocking screws [6] can be added if needed to ensure no loss of position occurs with fixator removal.

If LON is being used, only the lower locking screws of the IM nail are inserted, but if a motorized IM nail is being used, upper and lower locking screws are inserted.

### Illustrative cases

Case number 3 (Fig. 7): 20-year-old male patient who suffered shortening of the right femur due to contralateral side (left femur) overgrowth following childhood trauma, limb length discrepancy (LLD) was 5 cm. (A) shows the pre-operative radiograph with a slight lateral MAD, (B) shows the proposed planning to achieve normal limb alignment after lengthening is completed, (C) shows the reproduction of the planning and achieving normal alignment at the end of lengthening with no residual MAD, (D) shows the final radiograph after regenerate consolidation with normal limb alignment and no residual MAD.

**Fig. 8:**
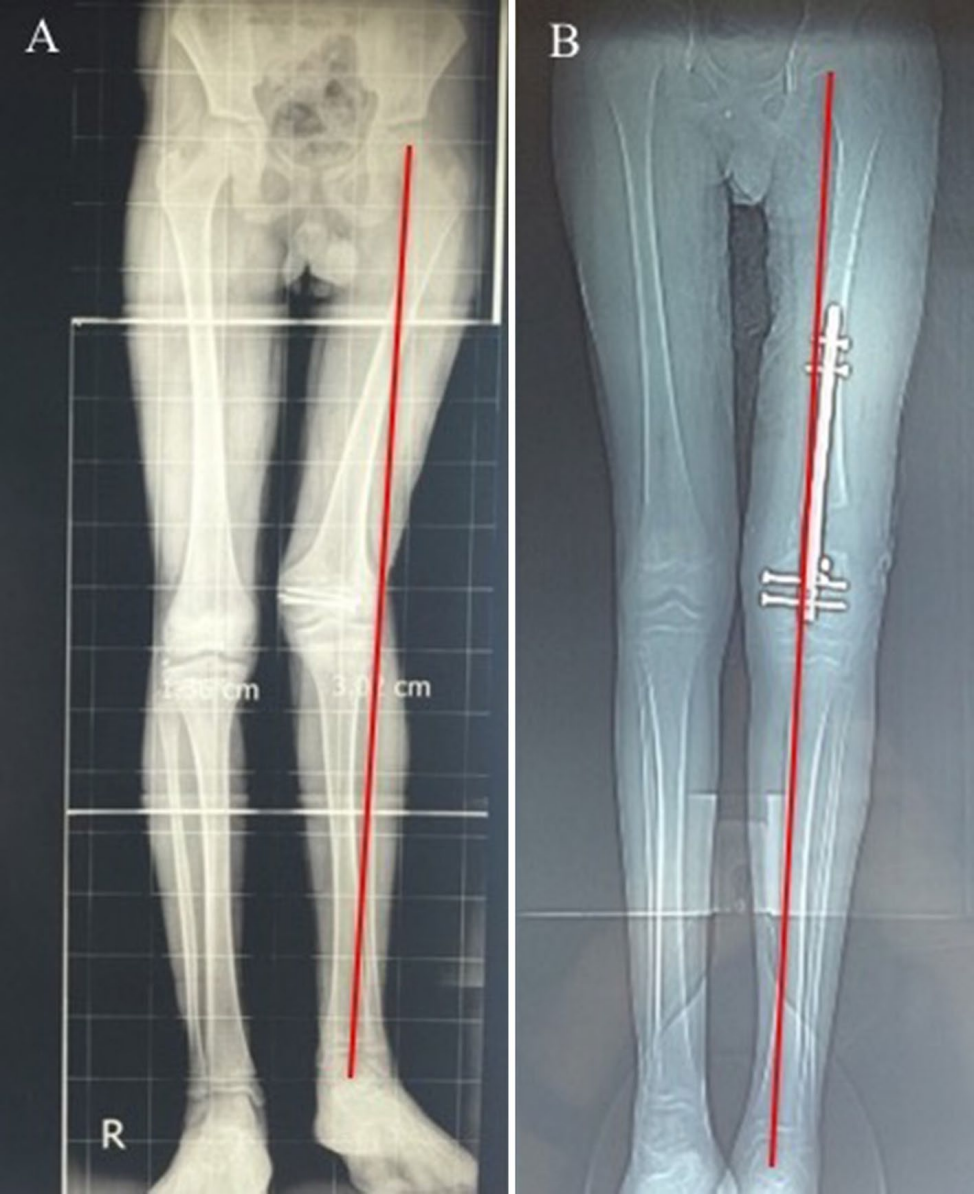
**a** Pre-operative radiograph showing the combined shortening and valgus malalignment of the left femur, **b** the reproduction of the planning and achieving normal alignment at the end of lengthening with no residual MAD

Case number 8 (Fig. 8): 13-year-old female patient suffering LLD of 6 cm following distal femur physeal arrest following trauma, valgus mal-alignment is evident as well. (A) is the pre-operative radiograph showing the combined shortening and valgus mal-alignment of the left femur, (B) shows the reproduction of the planning and achieving normal alignment at the end of lengthening with no residual MAD.

**Fig. 9:**
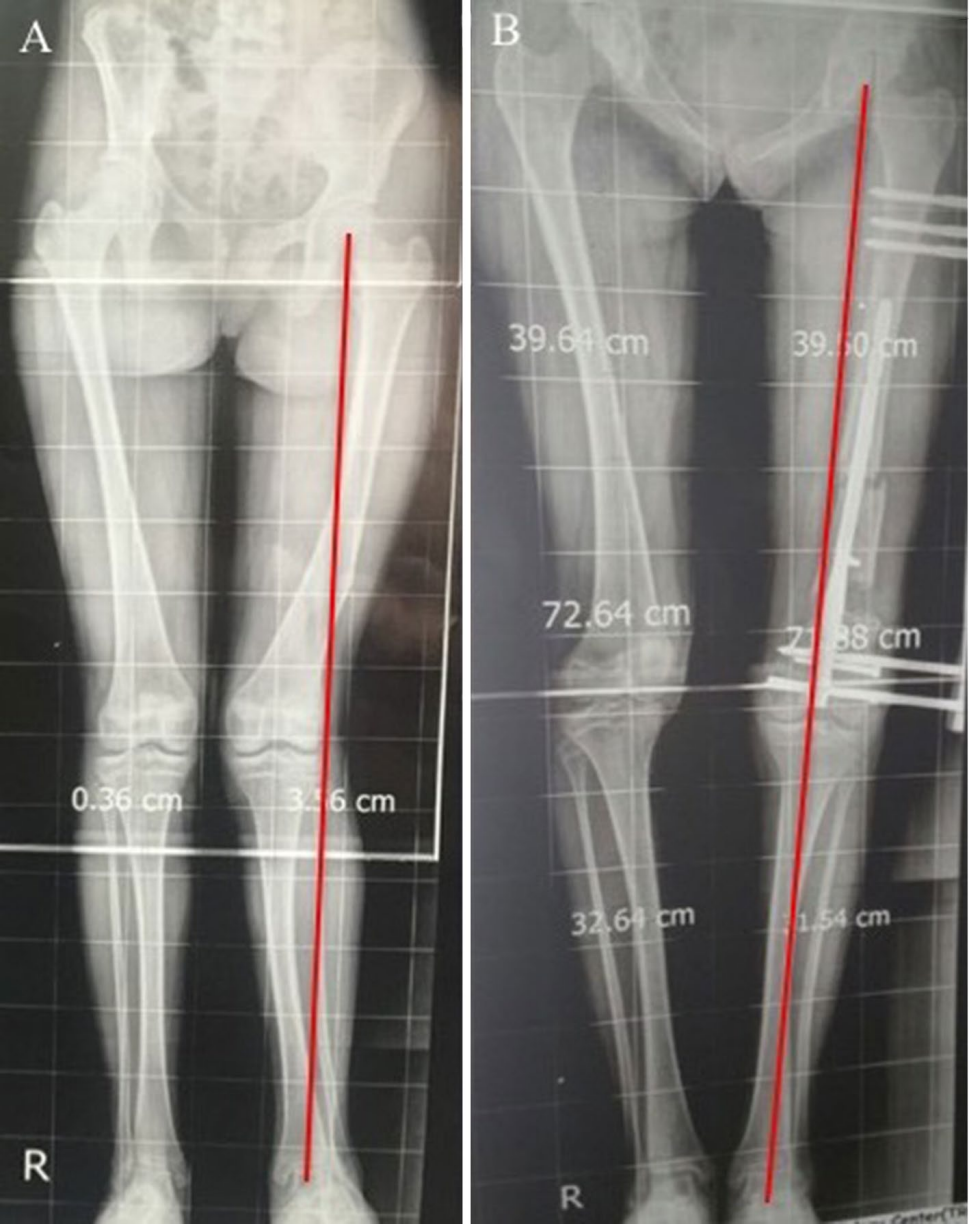
**a** Pre-operative radiograph showing the combined shortening and valgus malalignment of the left femur, **b** the reproduction of the planning and achieving normal alignment at the end of lengthening with no residual MAD

Case number 5 (Fig. 9): 12-year-old male patient suffering LLD of 4 cm following distal femur physeal arrest following trauma, valgus malalignment is evident as well. (A) shows the pre-operative radiograph showing the combined shortening and valgus malalignment of the left femur, (B) shows the reproduction of the planning and achieving normal alignment at the end of lengthening with no residual MAD.

## Results

The desired length was achieved in all patients with good quality of the regenerate. Normal limb alignment was achieved in all patients at the end of lengthening. No patient needed bone grafting, and there were no cases of deep wound infection.

Table 1 shows patients’ results regarding final MAD and aLDFA.

**Table 1:** Patients’ data

Case number	Age in years	Sex	Deformity associated	Deformity apex	Shortening in cm	Pre-operative aLDFA angle in degrees	Final aLDFA angle in degrees	Pre-operative MAD in cm	Final MAD in cm
1	18	M	None	–	4	82	81	0	0
2	14	M	Varus	Metaphyseal	4	90	82	+ 3	0
3	20	F	None	–	5	81	80	0	0
4	15	F	Varus	Metaphyseal	5	92	81	+ 3.4	0
5	14	M	Varus	Metaphyseal	5	90	82	+ 3.6	0
6	12	M	Valgus	Metaphyseal	4	73	80	− 2.2	0
7	16	M	None	–	8	81	80	0	0
8	13	F	Valgus	Metaphyseal	6	75	80	− 1.8	0

*aLDFA* anatomical lateral distal femoral angle, *MAD* mechanical axis deviation

## Discussion

Contrary to lengthening with external fixators, in lengthening with or along IM nails, there is no room for postoperative adjustments. Thus, surgery should be planned and performed accurately.

Lengthening of the femur with or along IM nails happens along its anatomical axis which may create lateral mechanical axis deviation [2]. In the study by Burghardt et al. [2], they showed 1 mm lateral mechanical axis deviation for each 1 cm of lengthening when using a proximal entry nail.

When planning for such lengthening of the femur with IM nails, whether shortening is associated with frontal plane deformity or not, a different planning strategy is required. To finally align the mechanical axis and correct joint orientation angles when lengthening is completed, both the original deformity and that created by lengthening along the anatomical axis must be considered [1]. To the best of the author’s knowledge, only one method in the literature that can be used to plan for femoral lengthening with or along IM nails is the reverse planning method (RPM) described by Baumgart [1].

The author believes that this method could be an alternative to the reverse planning method (RPM) as it utilizes the concepts of the classic CORA method for deformity analysis and planning with which many surgeons are more familiar. The new method does not require drawing bone fragments on paper/graph paper or the use of more costly digital planning platforms. Lines can be drawn on regular long radiographs or using inexpensive digital planning platforms for example Bone Ninja^®^ [7] mobile application. Mathematical formula used in this method can be done using any smartphone calculator application or could be integrated into the Multiplier [8] mobile application.

Integration of this planning method in digital planning systems that use radiographs (which are becoming more common in orthopedic practice) like TraumaCad^®^ [9] would make its use easier for the clinicians.

The Resolution Axis Method (RAM) uses a *resolution* anatomical axis for retrograde IM nail passage and subsequent lengthening. This will prevent any mechanical axis deviation at the end of lengthening. There is no room to alter the nail passage with antegrade nailing as this has to follow the mid-diaphyseal line; hence, retrograde nailing is used. This allows a change of the IM nail passage in the wider lower metaphyseal portion of the femur as detailed in this method.

Variable studies have reported similar results and complications when comparing antegrade to retrograde femoral nailing. Retrograde femoral nailing does not seem to negatively impact knee function and is not associated with increased rate of knee sepsis [10–13].

As with any planning method, the exact reproduction of the pre-operative plan during surgery is required to achieve perfect final alignment. Mechanical axis change predicted by pre-operative planning is therefore accounted for in the final outcome. Insertion of the nail along the *resolution* anatomical axis based on the AMA calculation is of extreme importance.

## Conclusion

The Resolution Axis Method (RAM) is a new and alternative method that the author believes could provide a solution for planning when lengthening the femur along the anatomical axis using IM nails, whether a deformity is present or not.

## Electronic supplementary material

Below is the link to the electronic supplementary material.

Supplementary material 1 (PDF 29248 kb)
