# Supplementary material for: The Resolution Axis Method (RAM) for lengthening of the femur with or without associated frontal plane deformity (a new method)
Source: Strategies Trauma Limb Reconstr. 2018 May 24;13(2):109–18. doi: 10.1007/s11751-018-0312-3 (PMC6042223; doi:10.1007/s11751-018-0312-3)

The Resolution Axis Method (RAM) for Lengthening of The Femur with or without associated frontal plane deformity (A New Method)

Sherif Galal Hassan MD, PhD (Orthopedics)

- *Assistant Professor of Pediatric Orthopedics , Cairo University, Egypt*
- *Fellow of Pediatric Orthopedics, Children's Hospital of Pittsburgh*

Department of Orthopaedics & Traumatology
Kasr Al-Ainy School of Medicine
Cairo University

No Disclosures

Introduction

Lengthening with IM nails (e.g. PRECICE, Fitbone nails) or lengthening with fixators over IM nails (LON) is becoming popular.

The Problem

1# Lengthening of the femur along the anatomic axis causes lateral mechanical axis deviation MAD i.e. Valgus effect

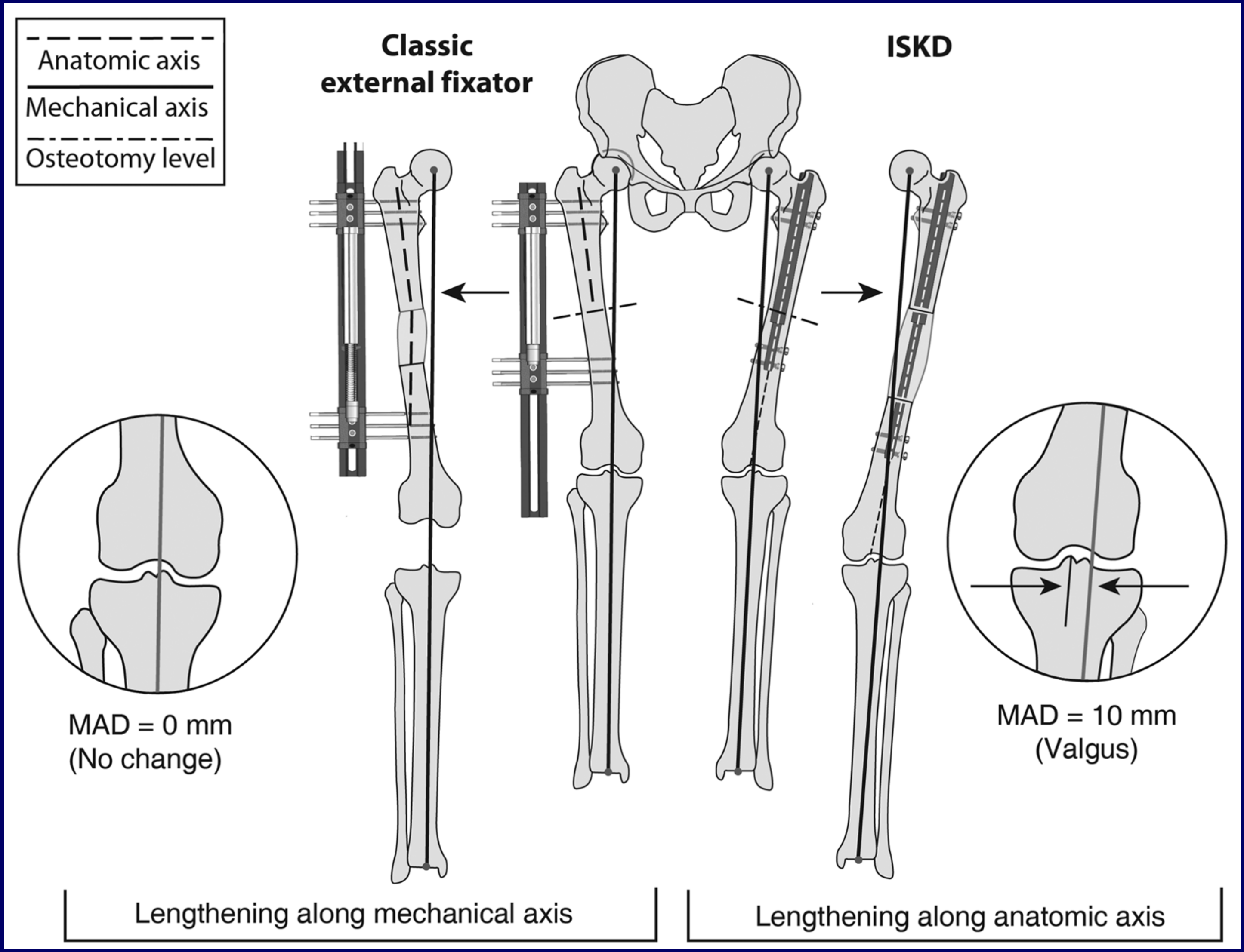

The effect on mechanical axis deviation of femoral lengthening with an intramedullary telescopic nail, Herzenberg et.al (JBJS Br. vol. 94-B, No. 9, September 2012)

The Problem

2# No room for alignment adjustments later

The Problem

3# Some cases has combined femoral shortening & deformity

We have to consider the existing deformity in addition to the deformity created by lengthening the femur along its anatomical axis (valgus effect)

The Question

How to plan for femoral lengthening with or along IM nails so as to end up with straight alignment at the completion of lengthening?

(putting into account the valgus effect created by such lengthening in addition to any existing deformity at the index procedure)

The Classic CORA deformity analysis method

- Each segment of the deformity would be represented by an axis line
- The anatomic axis of the upper femur would be the mid-diaphyseal line, the anatomic axis for the lower femur would be drawn referenced to the mechanical axis by 7º.
- The CORA (deformity apex) would be where the 2 axes lines intersect.
- Plan your osteotomy around the site of the CORA following osteotomy rules.

The CORA method shortcoming

1. It doesn’t put into account the deformity created by lengthening the femur with IM nails.

2. Not applicable in cases where the femur is only short but not deformed.

The new Proposal

This method is very similar to the classic CORA deformity analysis method, the only difference is:

When drawing the anatomic axis of the lower femoral fragment, instead of drawing it referenced to the mechanical axis at 7º, we use a smaller angle.

Solution

- Ditch the "classic" anatomic axis & use a "resolution" axis that's not 7º off the mechanical axis…..
- It's actually LESS than 7º off the mechanical axis

- To achieve a straight limb alignment when lengthening is completed, the center if the femoral head must be located along the upward extension of the mechanical axis of the lower femoral segment
- Hence the length of the femoral neck doesn’t change during lengthening.
- Thus the piriformis fossa (the upper limit of the anatomical axis) would assume a more medial position.

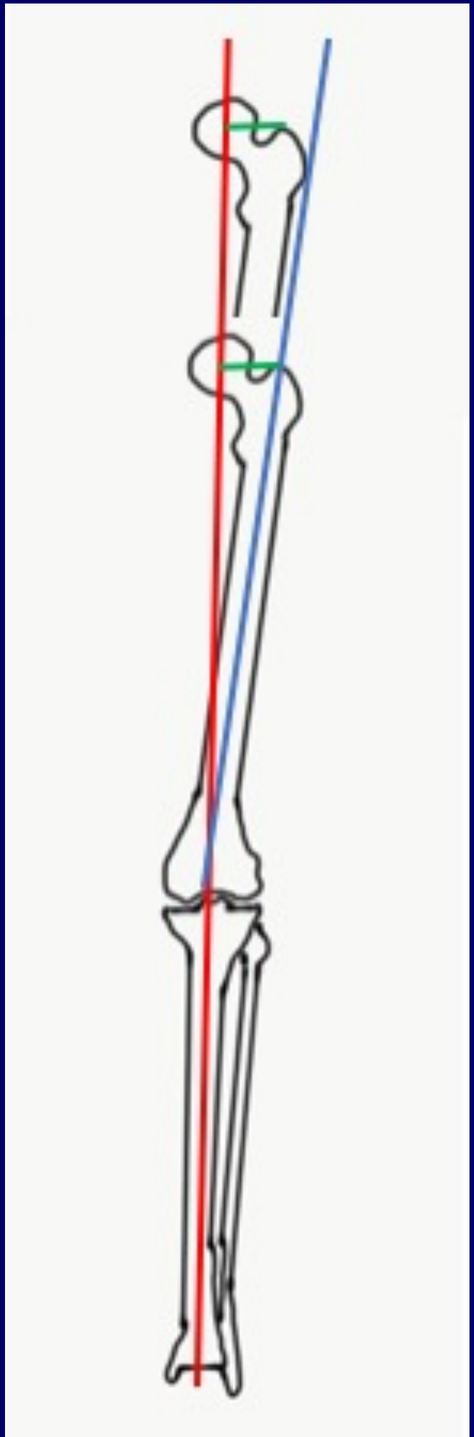

- To achieve a straight limb alignment when lengthening is completed, the center if the femoral head must be located along the upward extension of the mechanical axis of the lower femoral segment
- Hence the length of the femoral neck doesn't change during lengthening.
- Thus the piriformis fossa (the upper limit of the anatomical axis) would assume a more medial position.

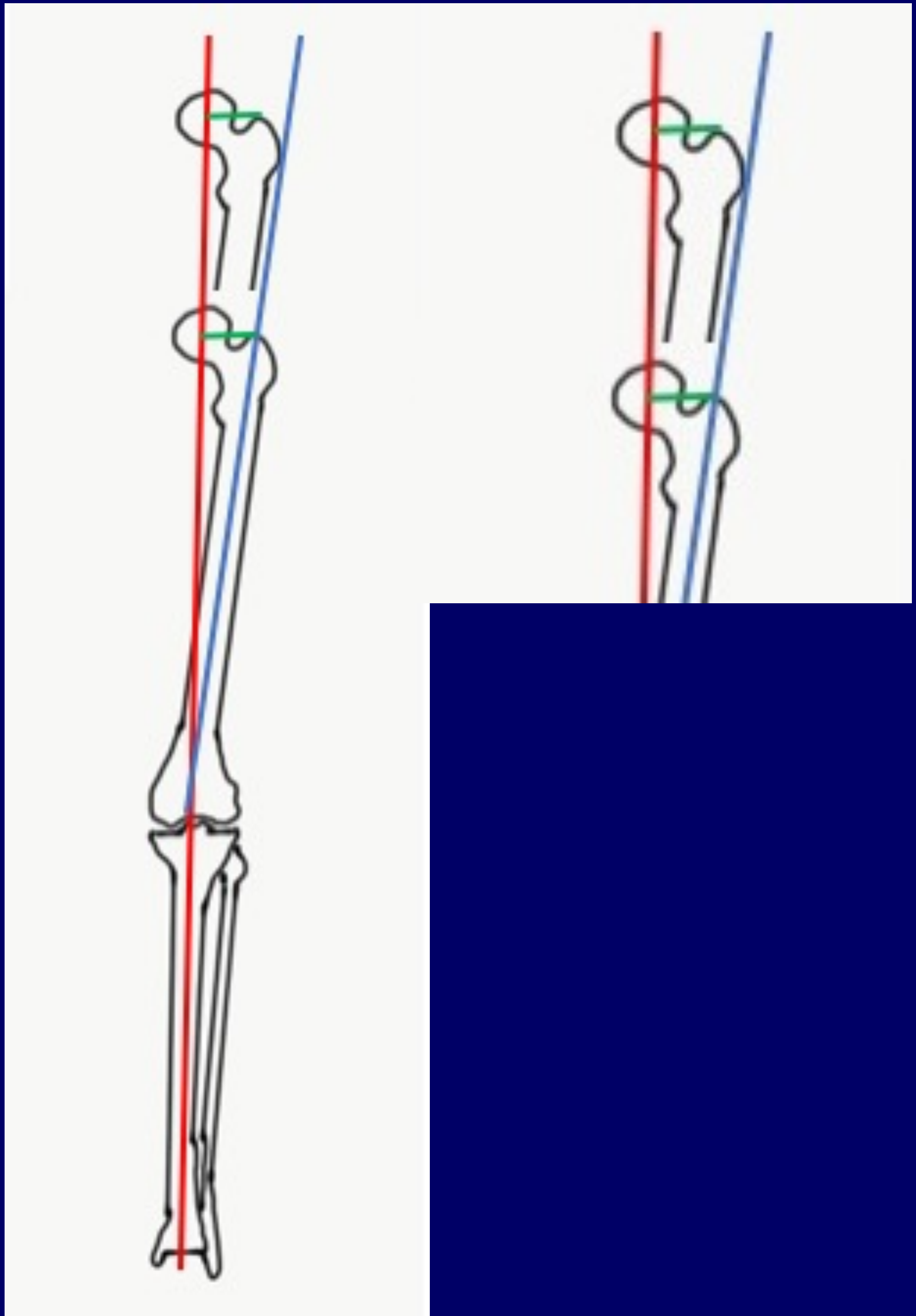

- This is opposite to what happens to the center of the femoral head which moves to a more lateral position (causing lateral MAD, i.e. valgus effect) when lengthening is done along the “classic” anatomical axis.

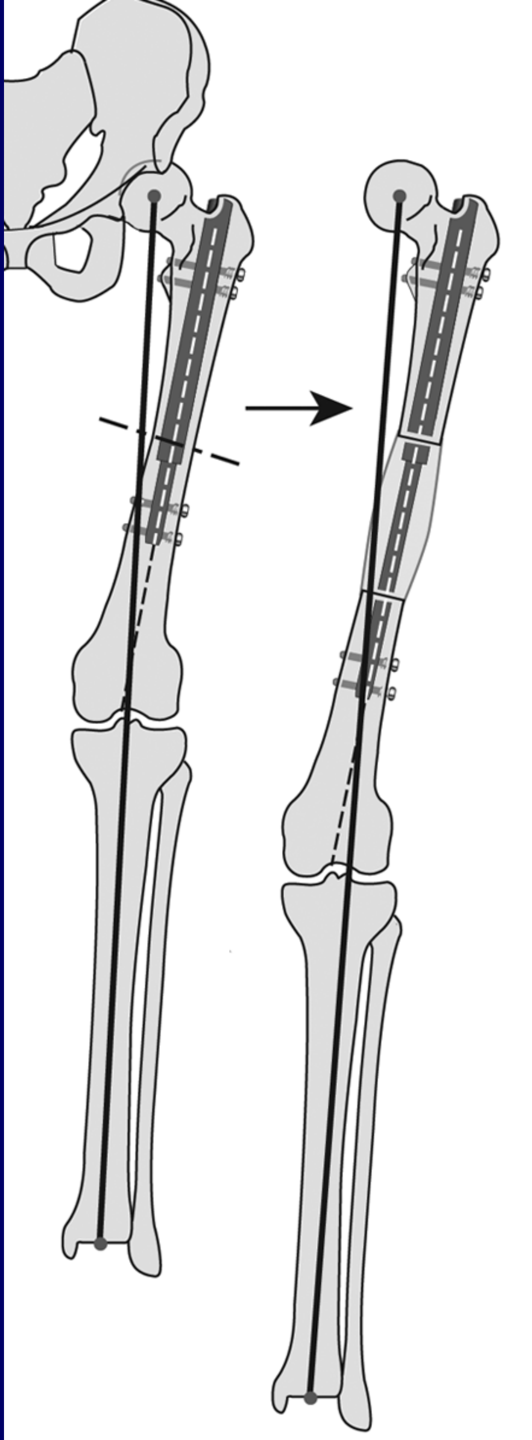

In the pre-lengthening situation the length of the femoral neck spans the distance between the "Classic" anatomical & the mechanical axis (the angle enclosed between both axes is 7° on average).

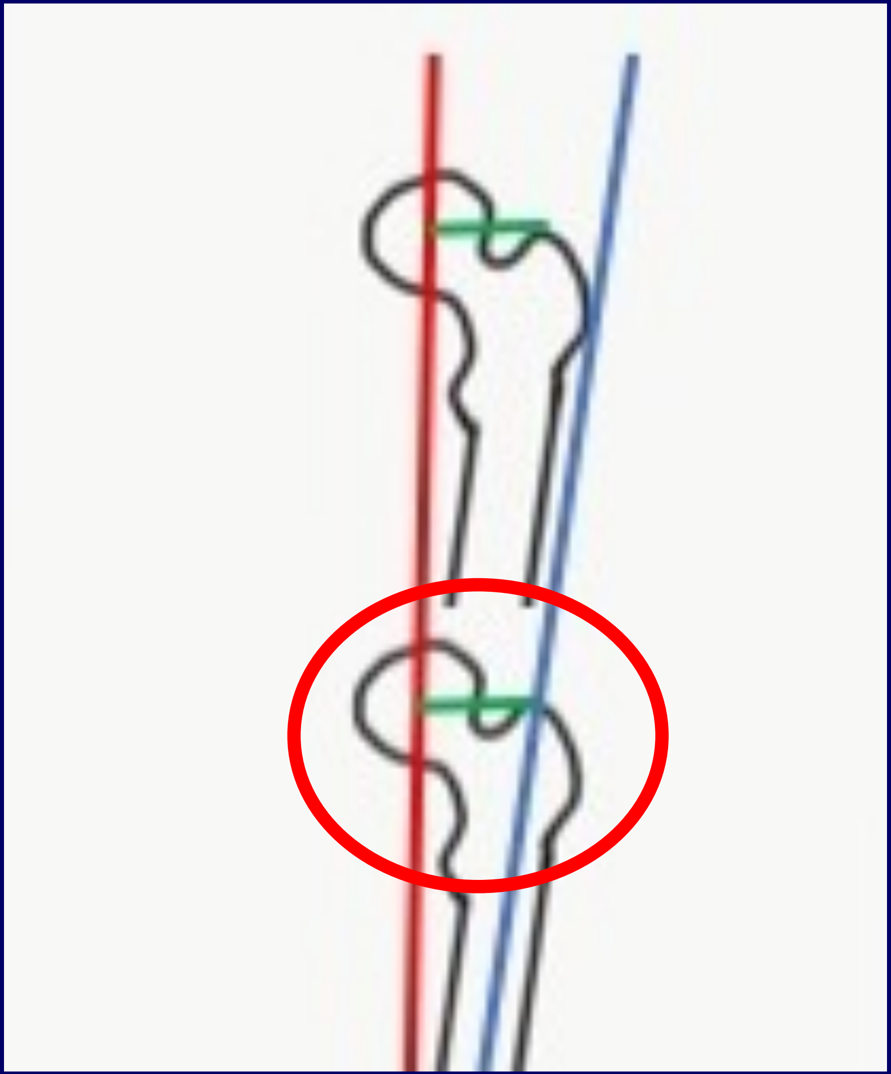

Thus in post-lengthening situation the length of the femoral neck won’t span the distance between the upward extensions of both the mechanical & the “classic" anatomical axis of the lower femoral segment.

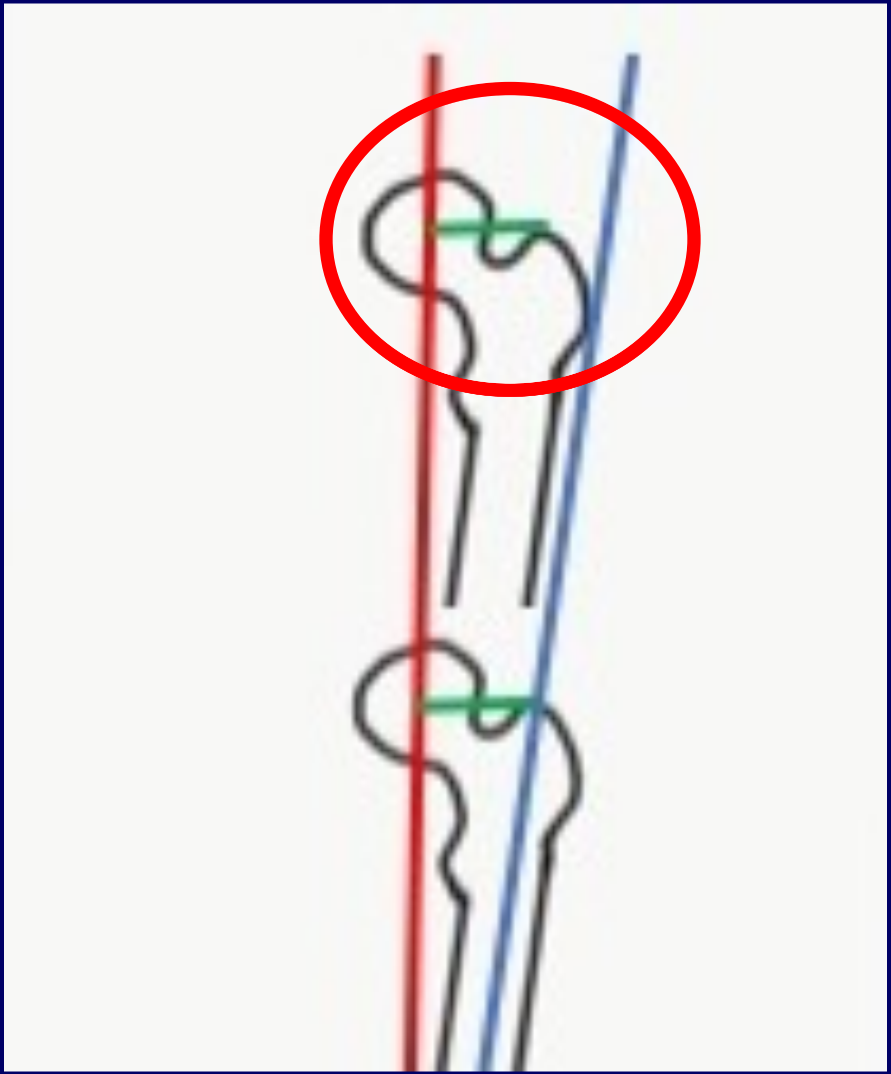

- Thus we can conclude that when lengthening the femur with IM nails we should use an axis that's referenced to the mechanical axis by an angle that's LESS than 7º.
- The more you lengthen, the smaller that angle would be.

$$\mathbf{Sin}\ \alpha` = \mathbf{c/\ c`\ X\ Sin}\alpha$$

- In this equation *c*, *c*` & α are known, so we can calculate sin α` & by using the inverse trigonometric function of " arcsin " on any calculator we can accurately estimate the (new) AMA angle.

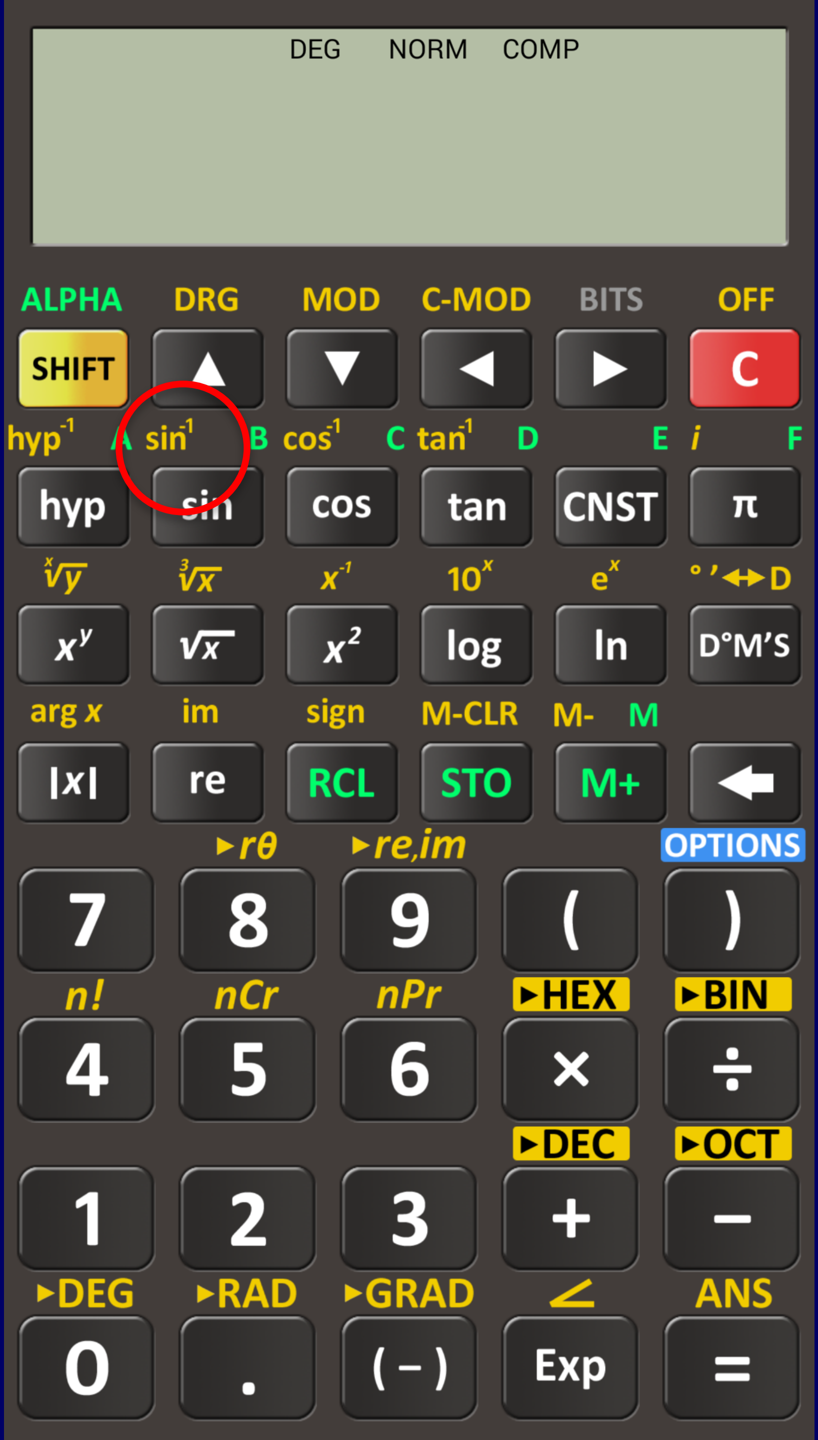
DEG NORM COMP
ALPHA DRG MOD C-MOD BITS OFF
SHIFT C
hyp⁻¹ A sin⁻¹ B cos⁻¹ C tan⁻¹ D E i F
hyp sin cos tan CNST π
x⁻¹ 10ˣ eˣ °'↔D
xʸ x² log ln D°M'S
arg x im sign M-CLR M- M
|x| re RCL STO M+
►rθ ►re,im OPTIONS
7 8 9 ( )
n! nCr nPr ►HEX ►BIN
4 5 6 × ÷
►DEC ►OCT
1 2 3 + −
►DEG ►RAD ►GRAD ANS
0 . (−) Exp =

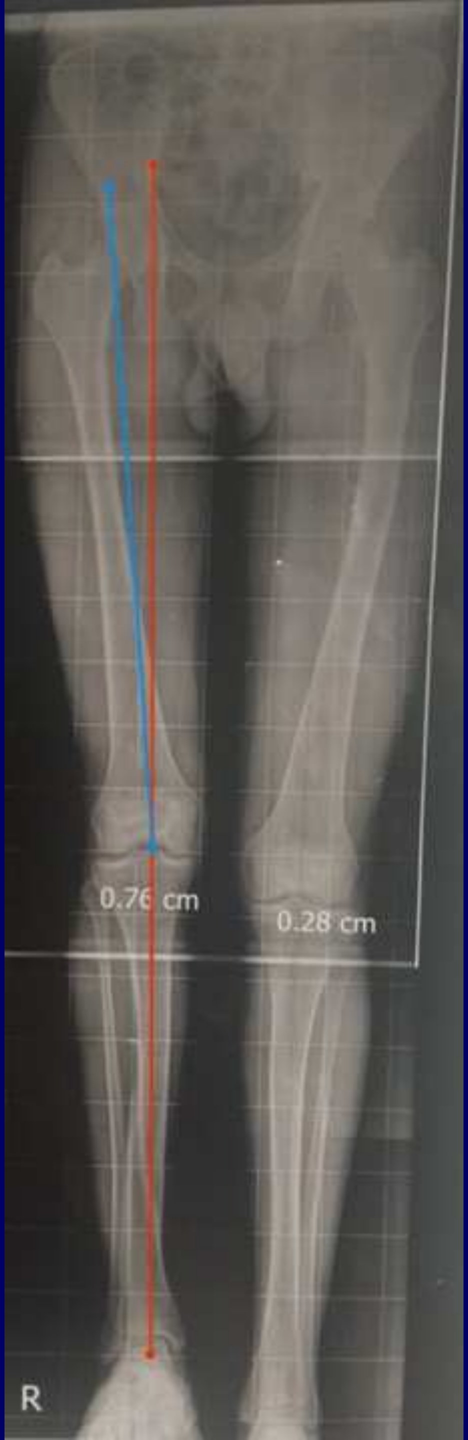

0.76 cm
0.28 cm
R

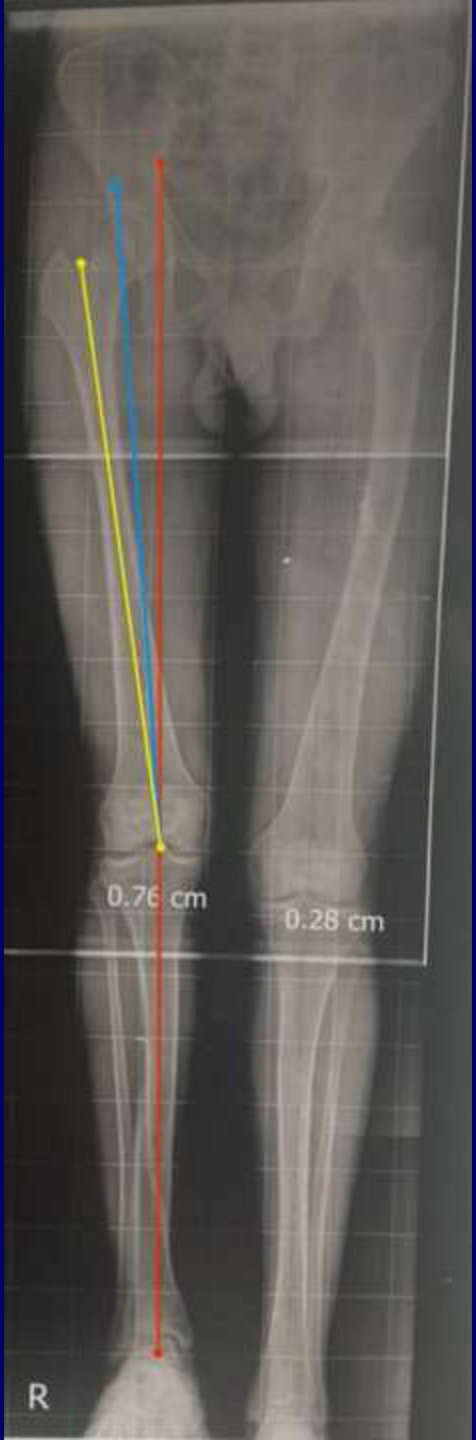

0.76 cm
0.28 cm
R

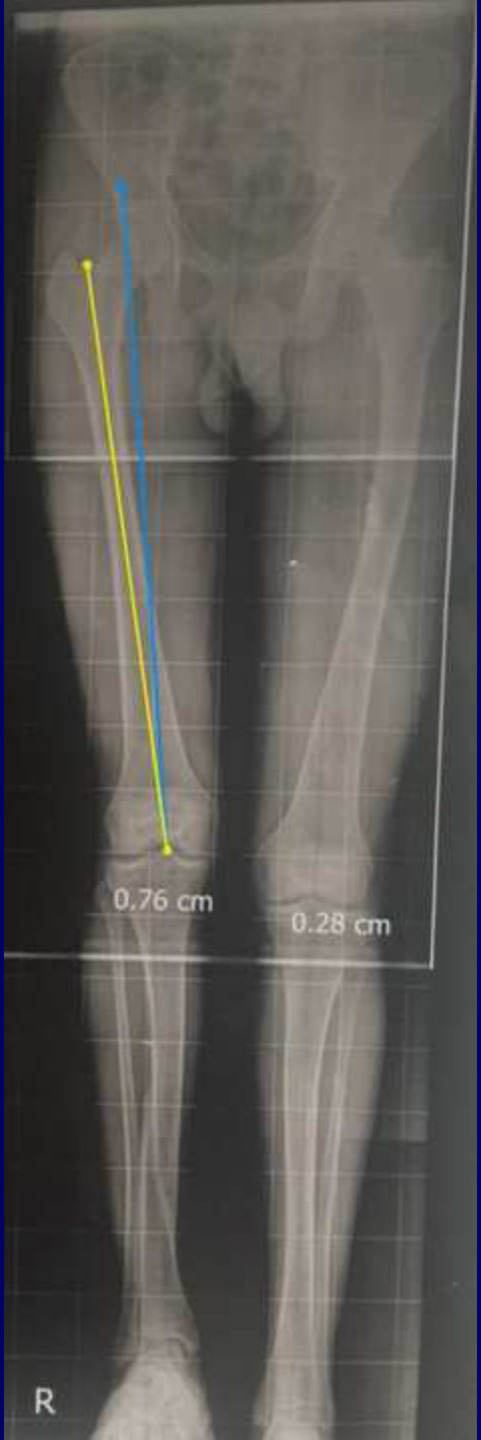

0.76 cm
0.28 cm
R

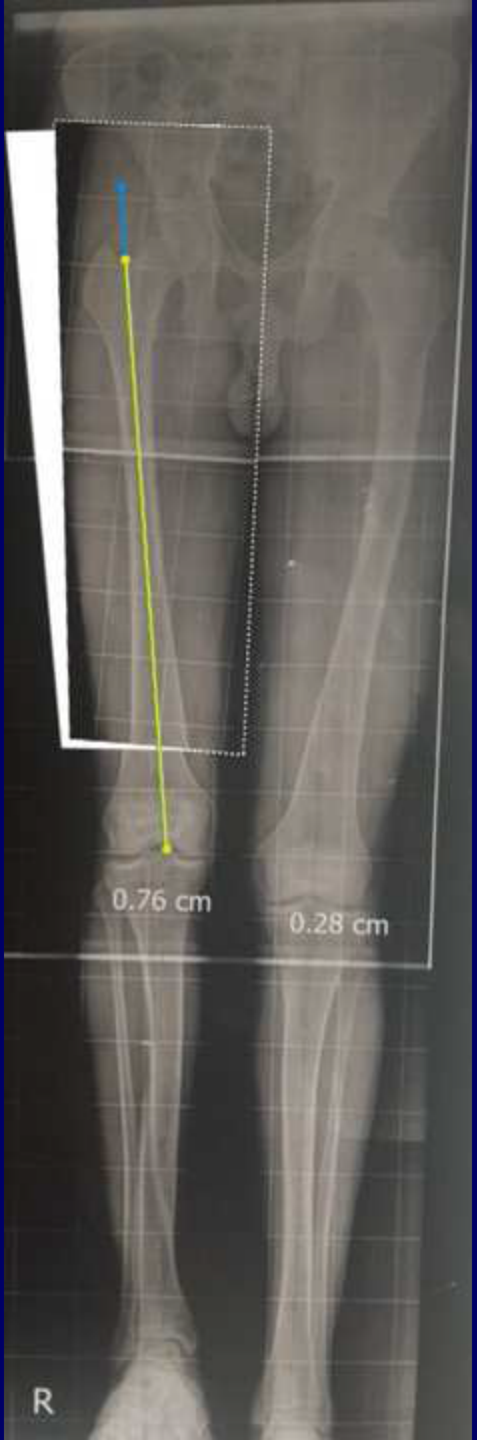
0.76 cm
0.28 cm
R

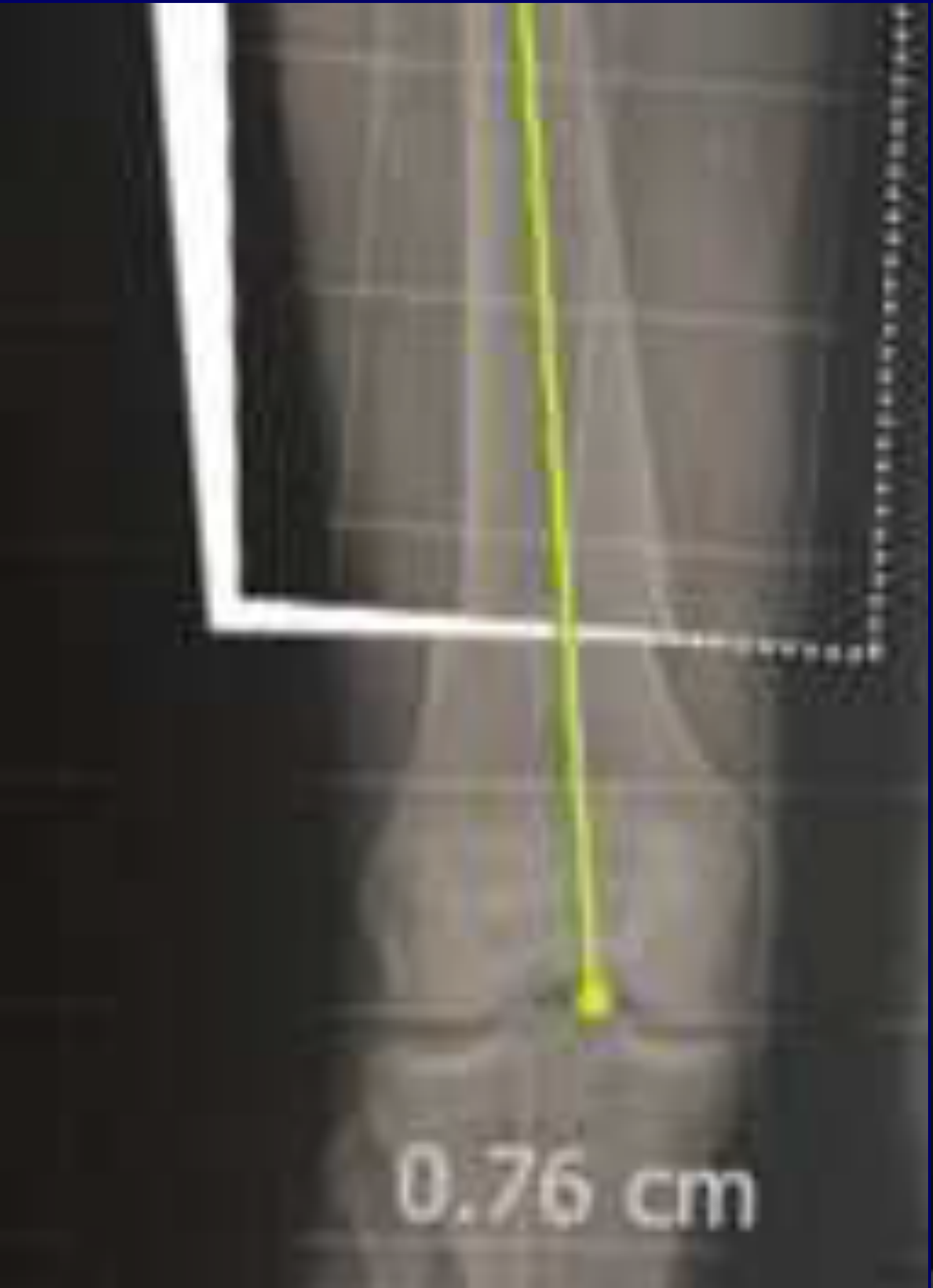
0.76 cm

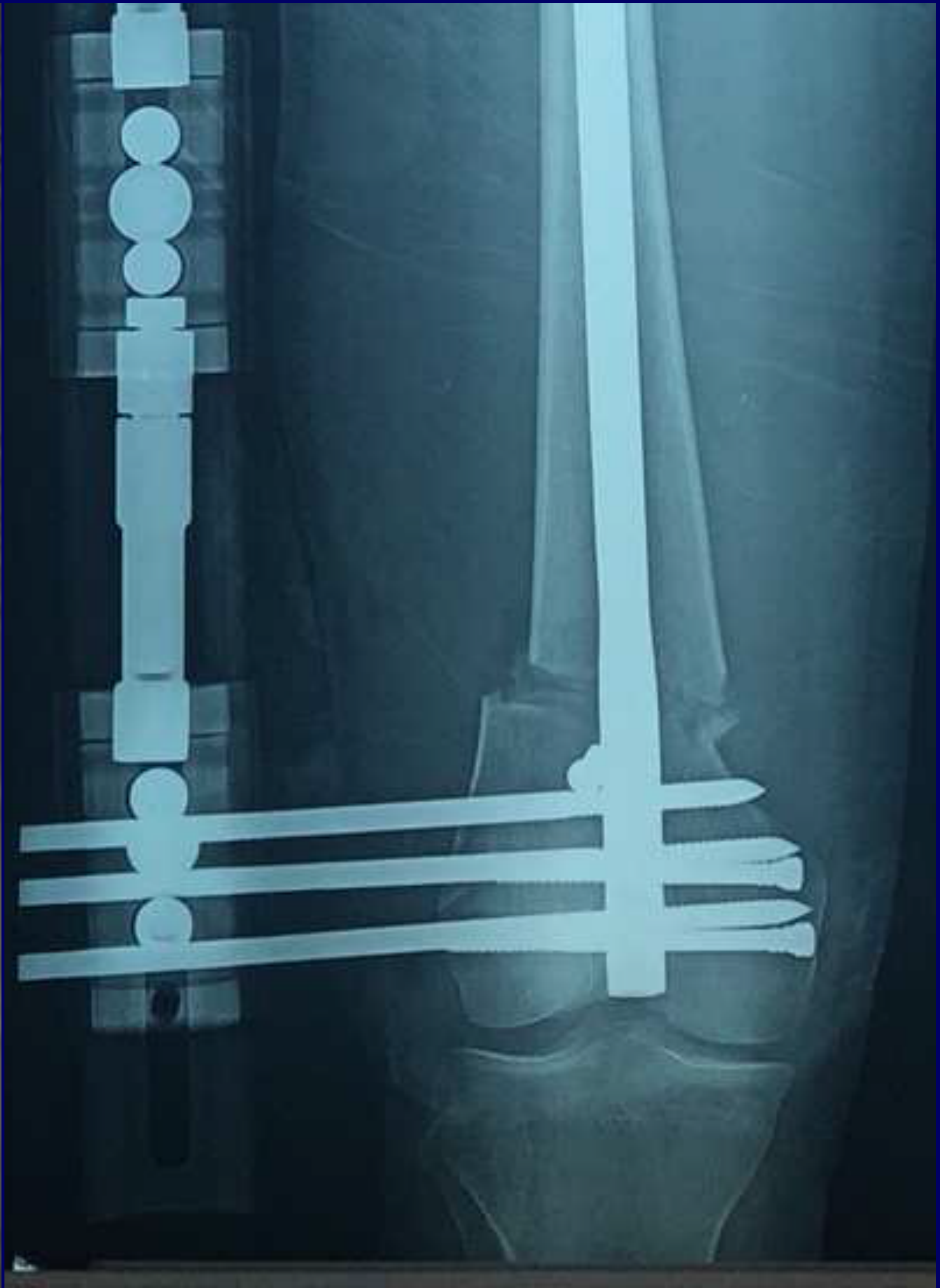

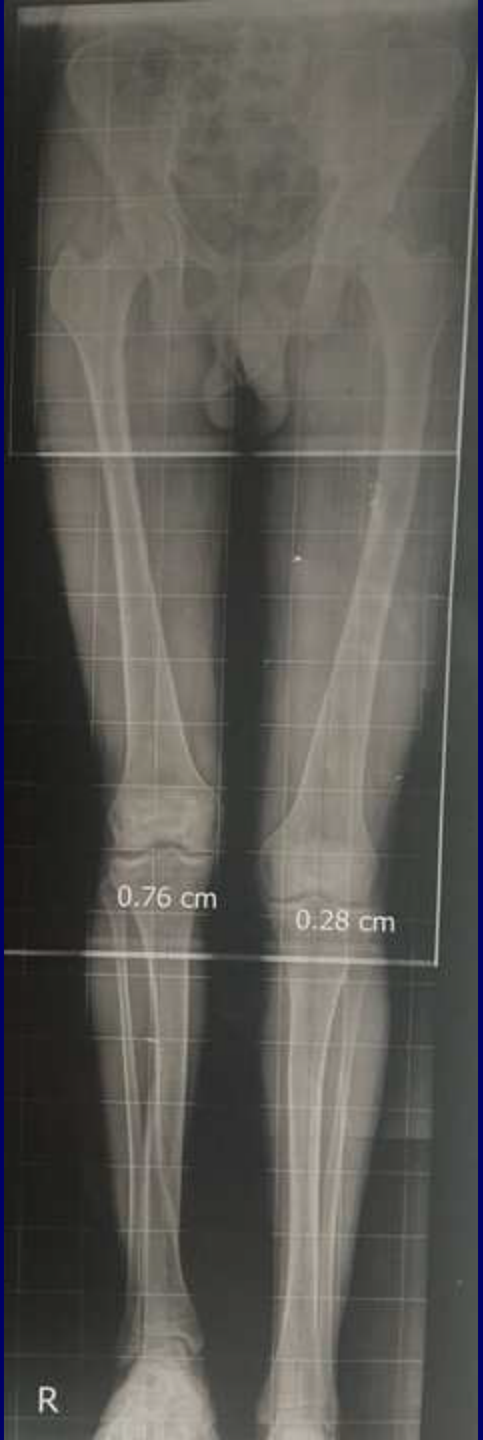
0.76 cm
0.28 cm
R

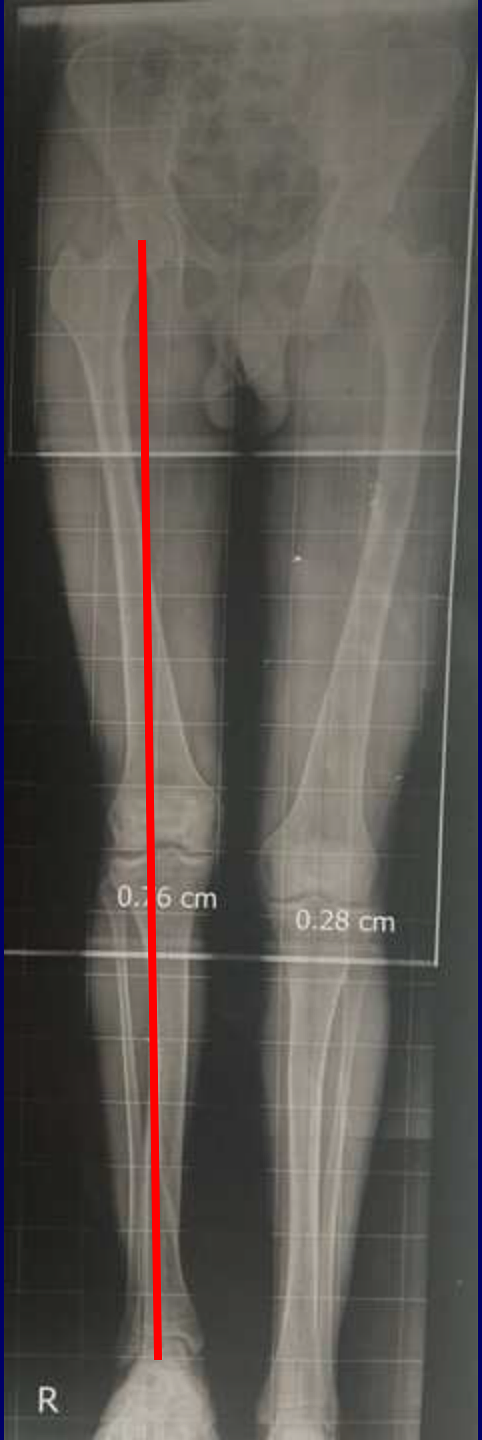
0.28 cm
R

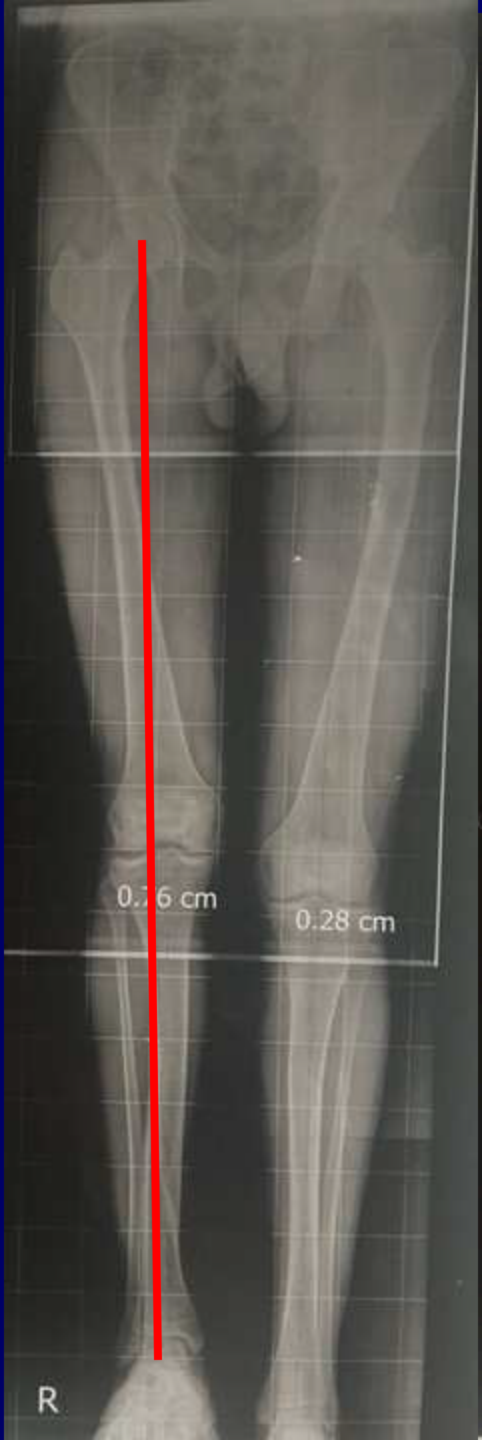
0.28 cm
R
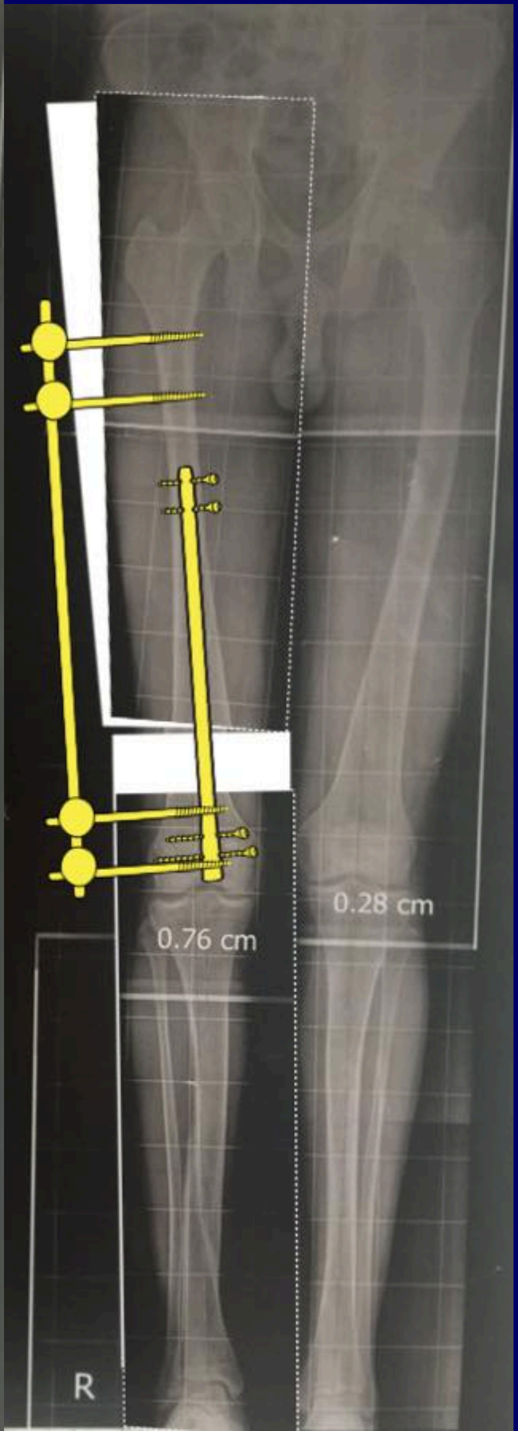
0.76 cm
0.28 cm
R

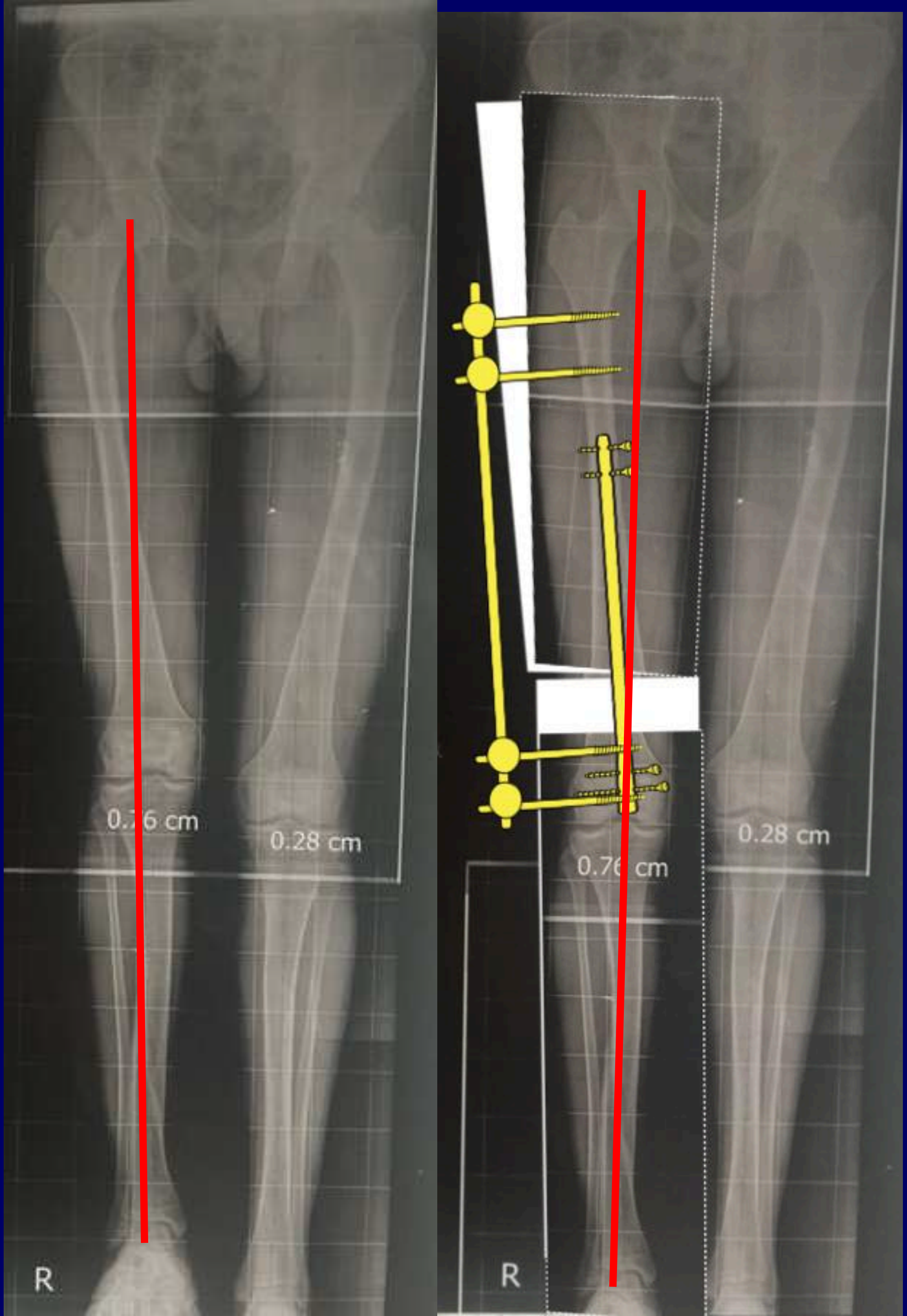
0.28 cm
R
0.76 cm
0.28 cm
R

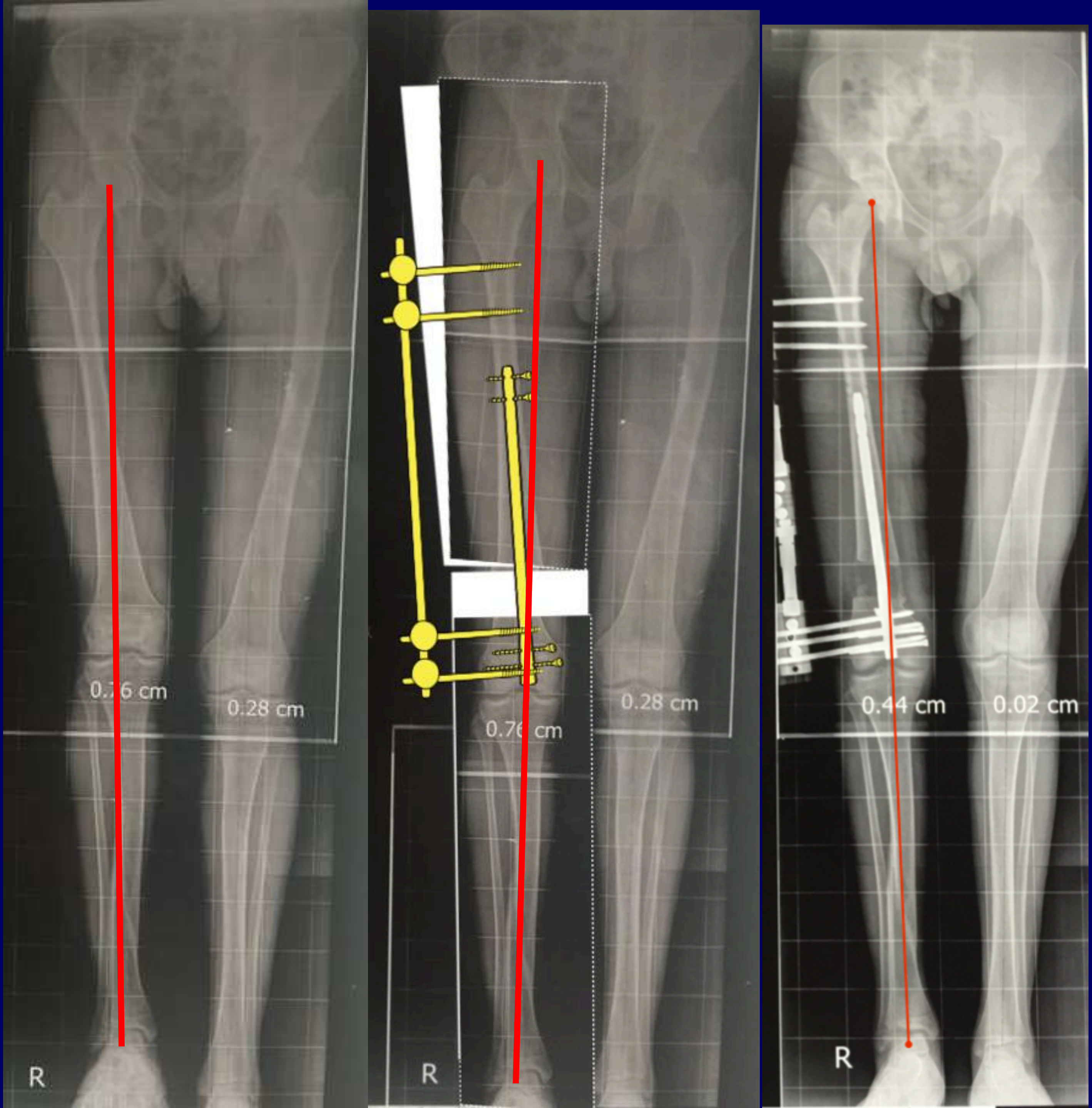
0.28 cm
R
0.28 cm
R
0.44 cm
0.02 cm
R

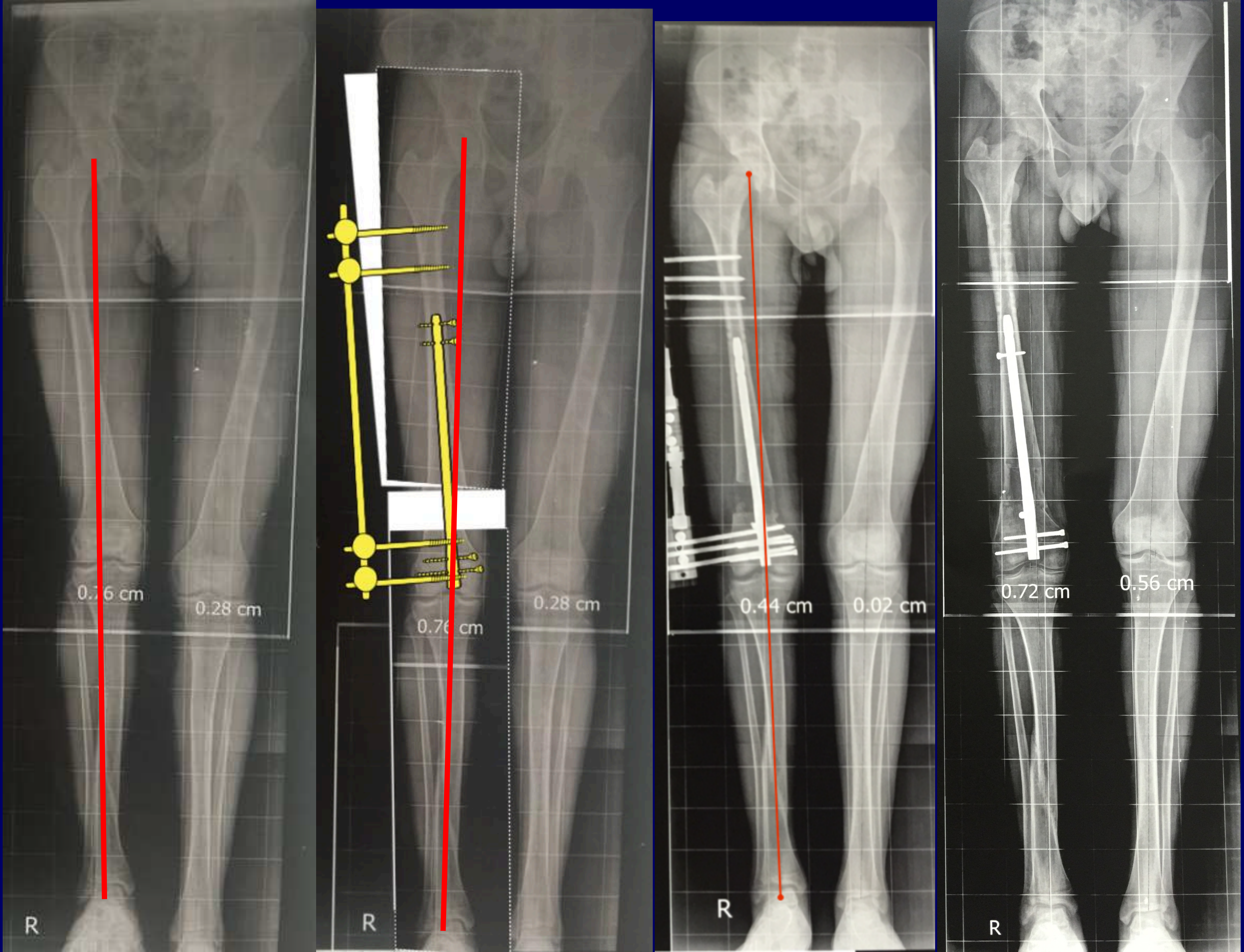

R
0.28 cm
R
0.28 cm
R
0.44 cm
0.02 cm
0.72 cm
0.56 cm
R

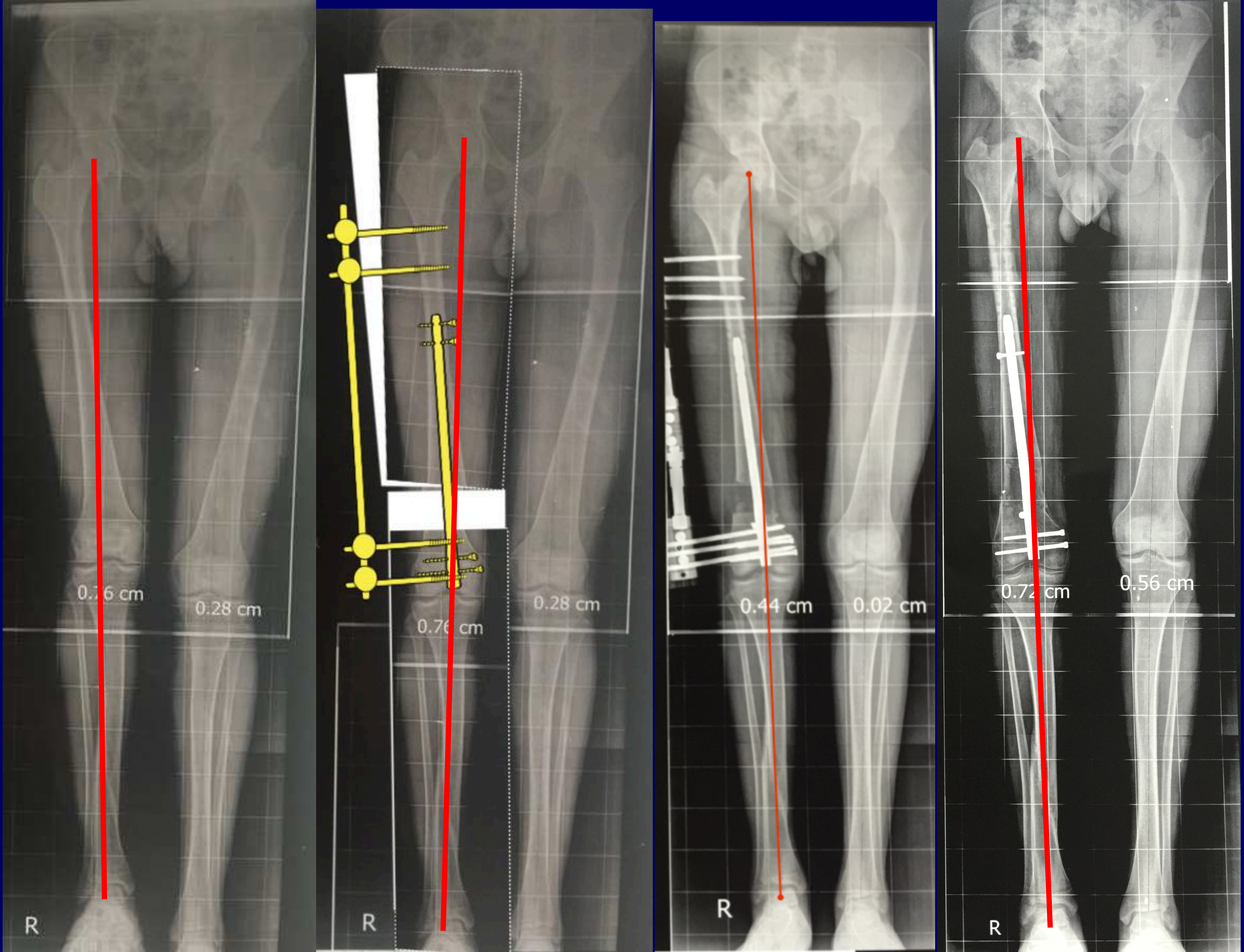

0.28 cm
R
0.28 cm
R
0.44 cm
0.02 cm
R
0.56 cm
R

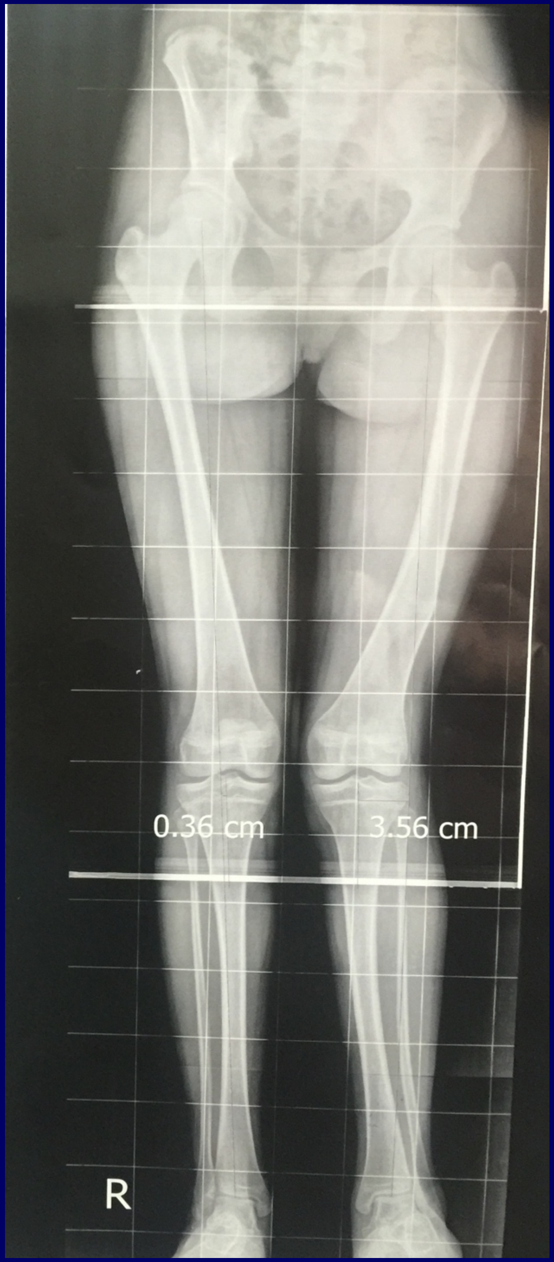
0.36 cm
3.56 cm
R

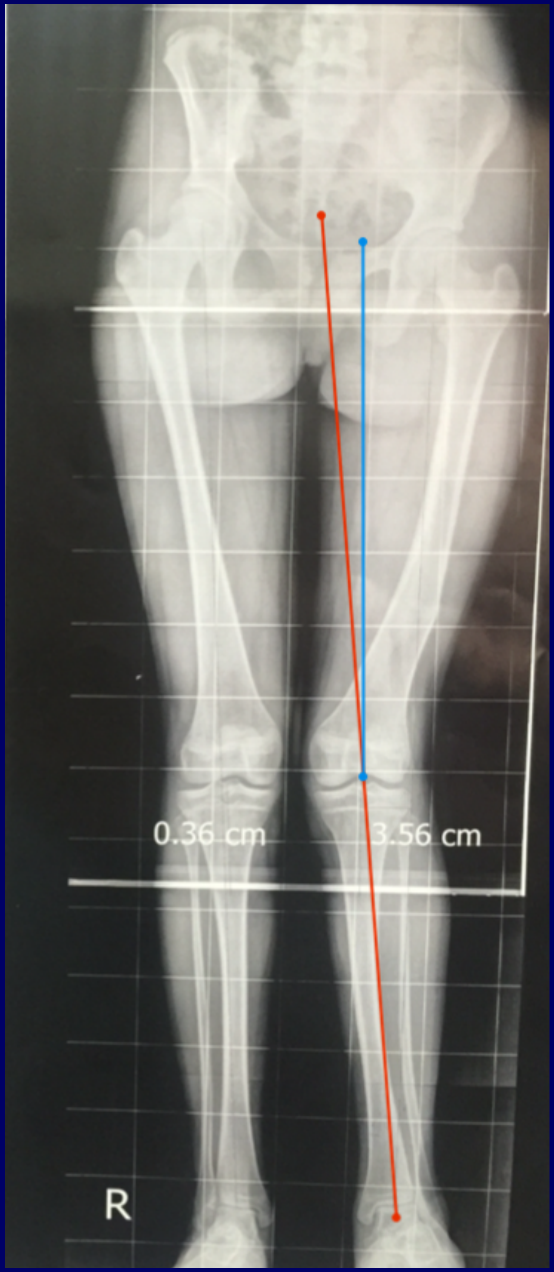
0.36 cm
3.56 cm
R

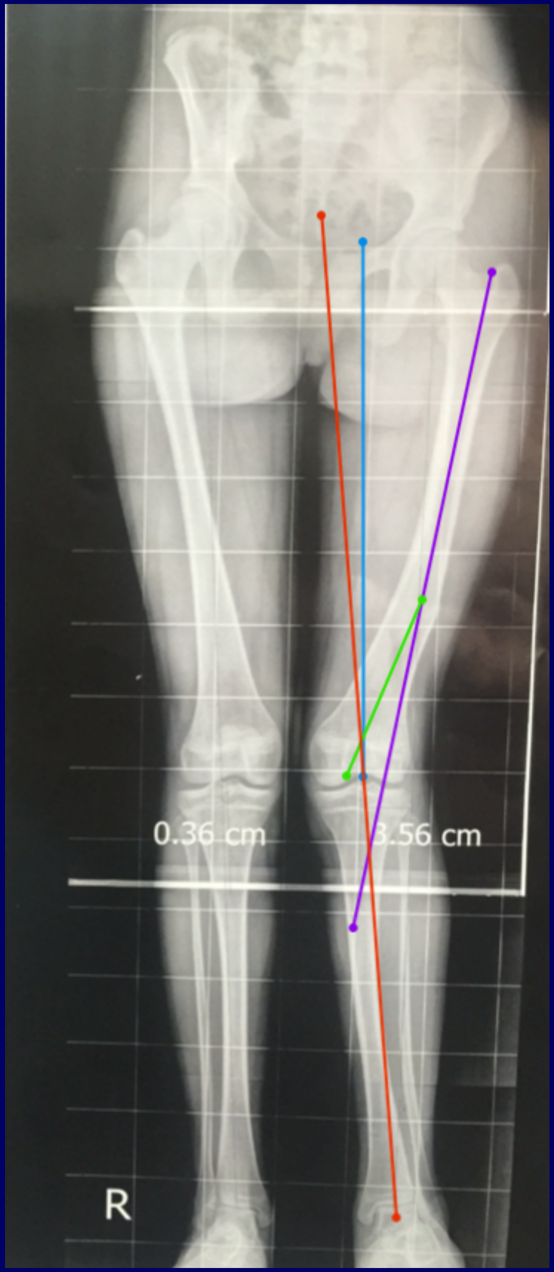
0.36 cm
3.56 cm
R

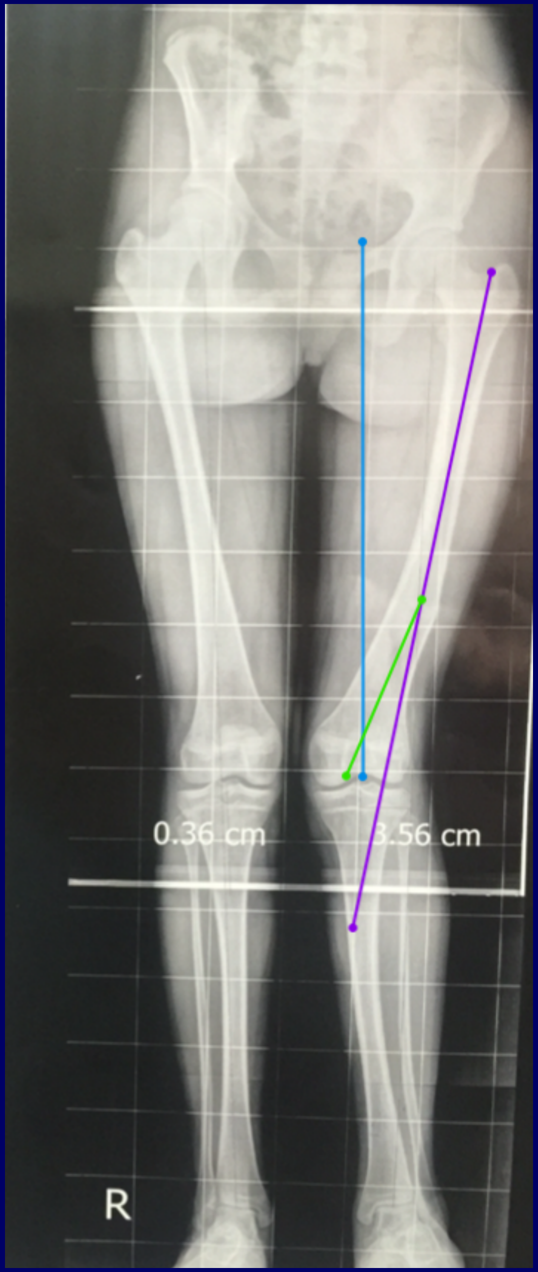
0.36 cm
3.56 cm
R

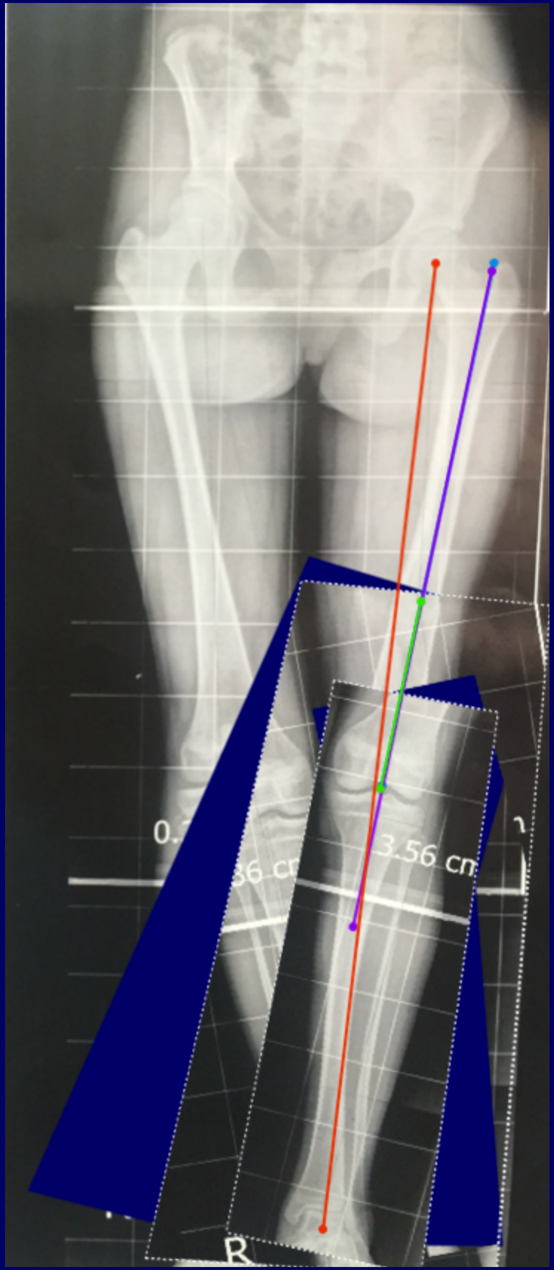
R

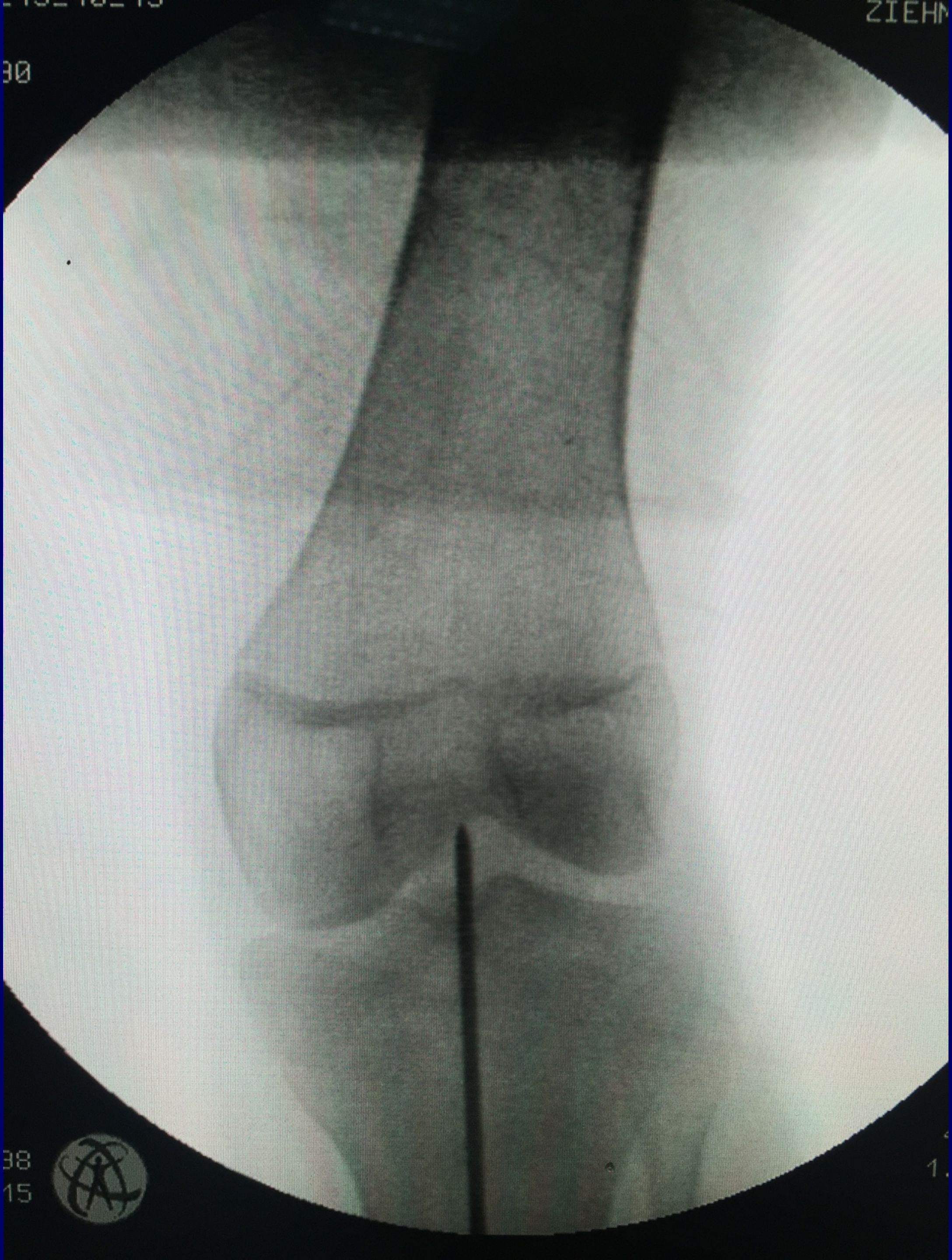

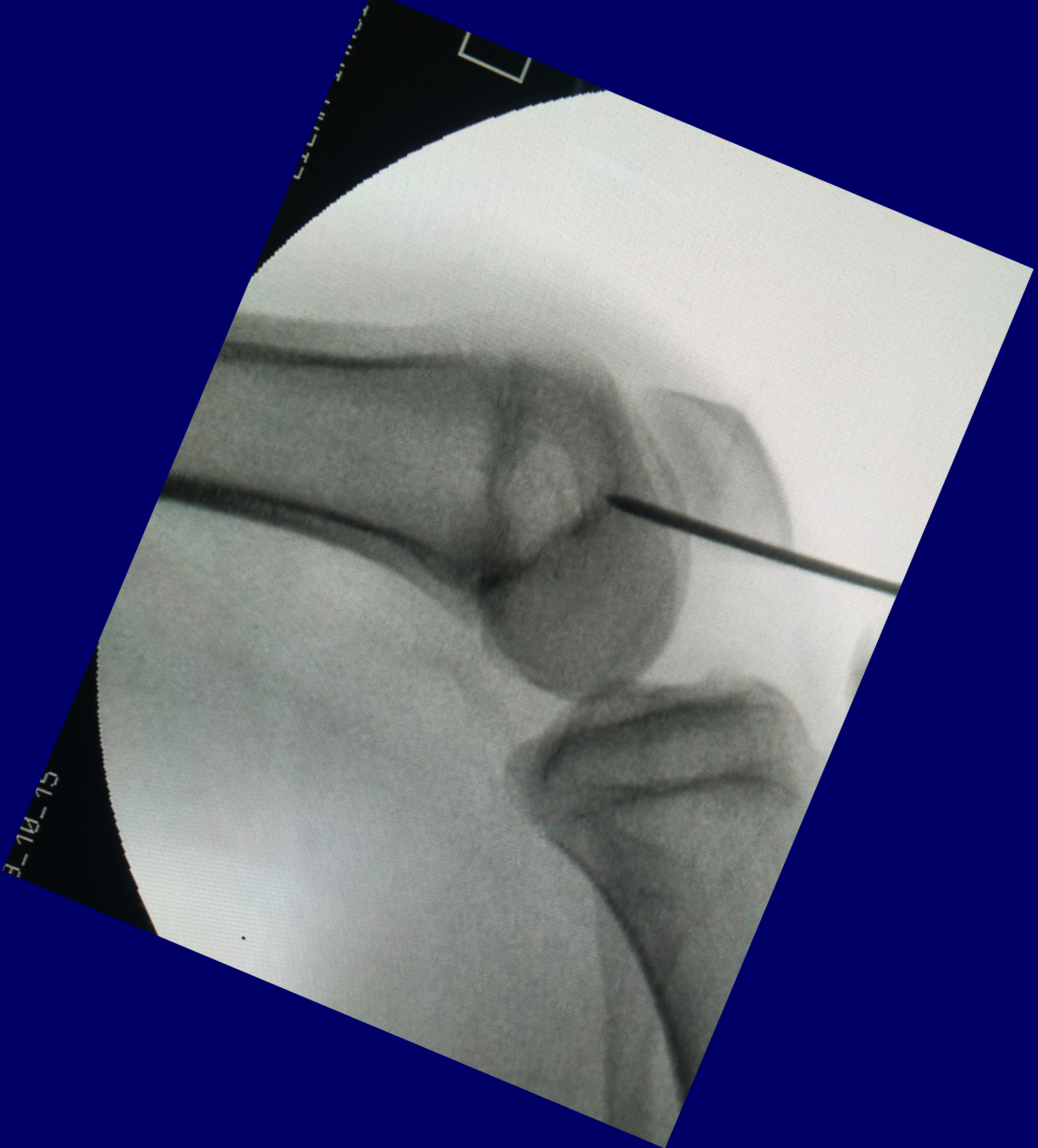

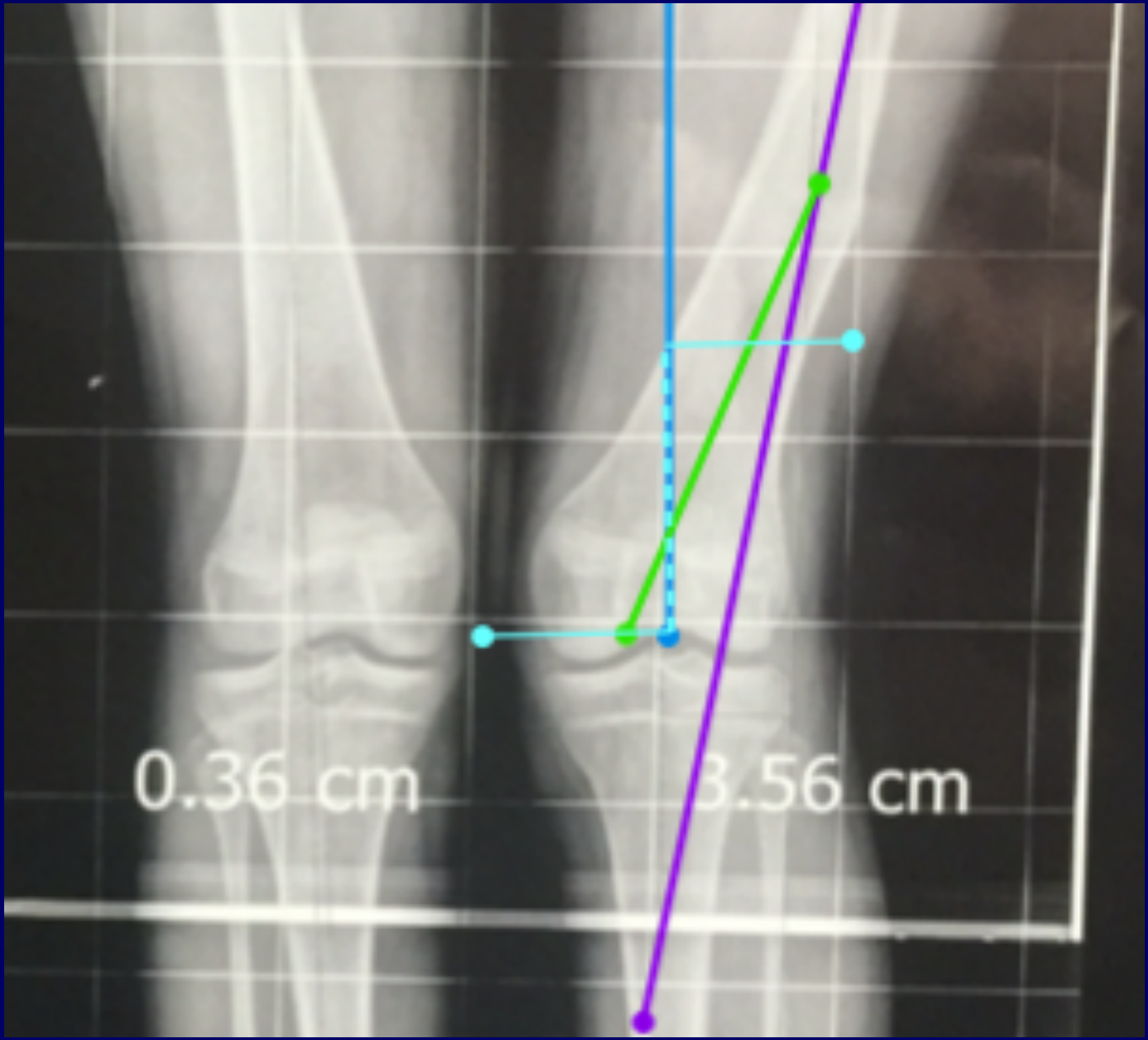
0.36 cm
3.56 cm

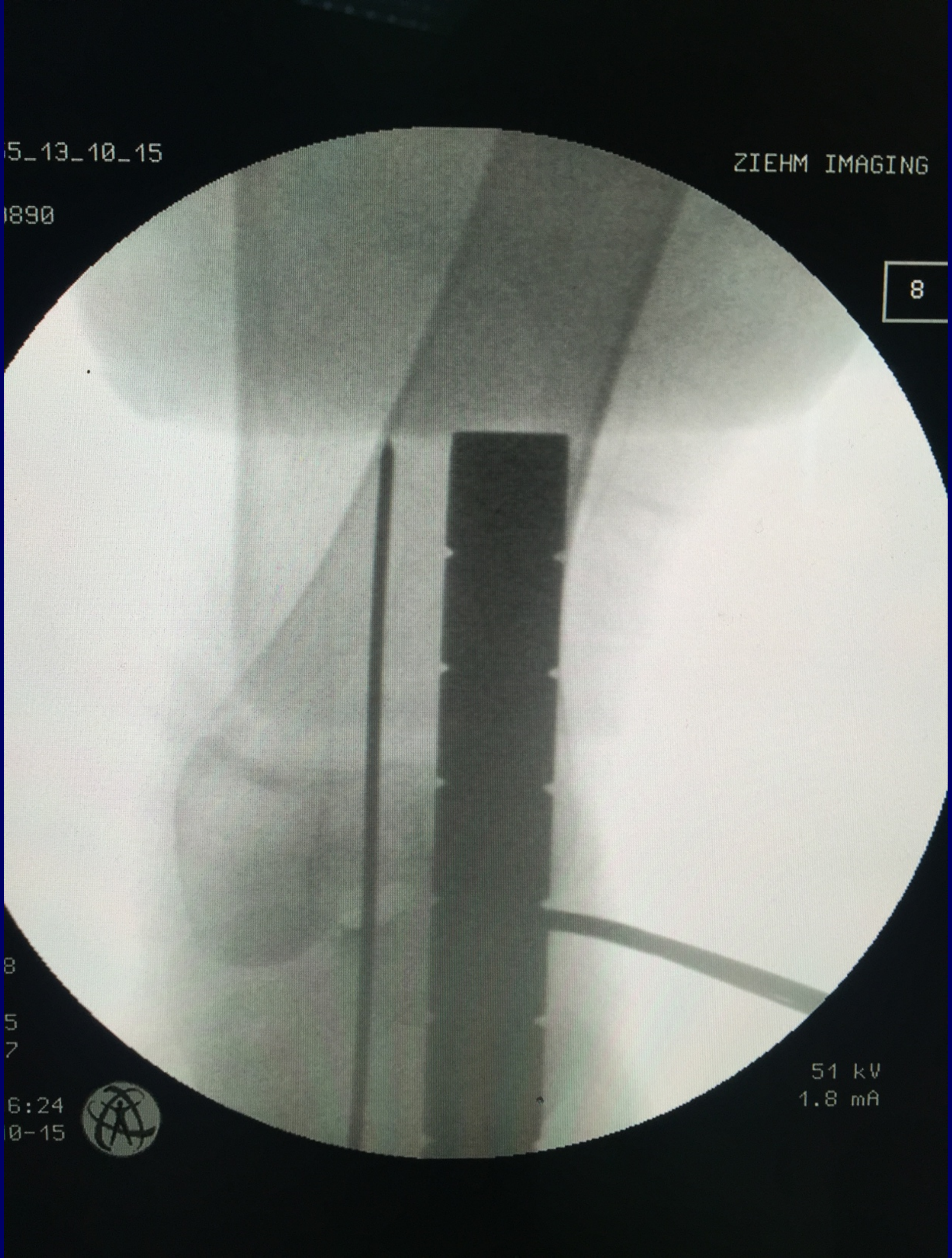
ZIEHM IMAGING
8
51 kV
1.8 mA

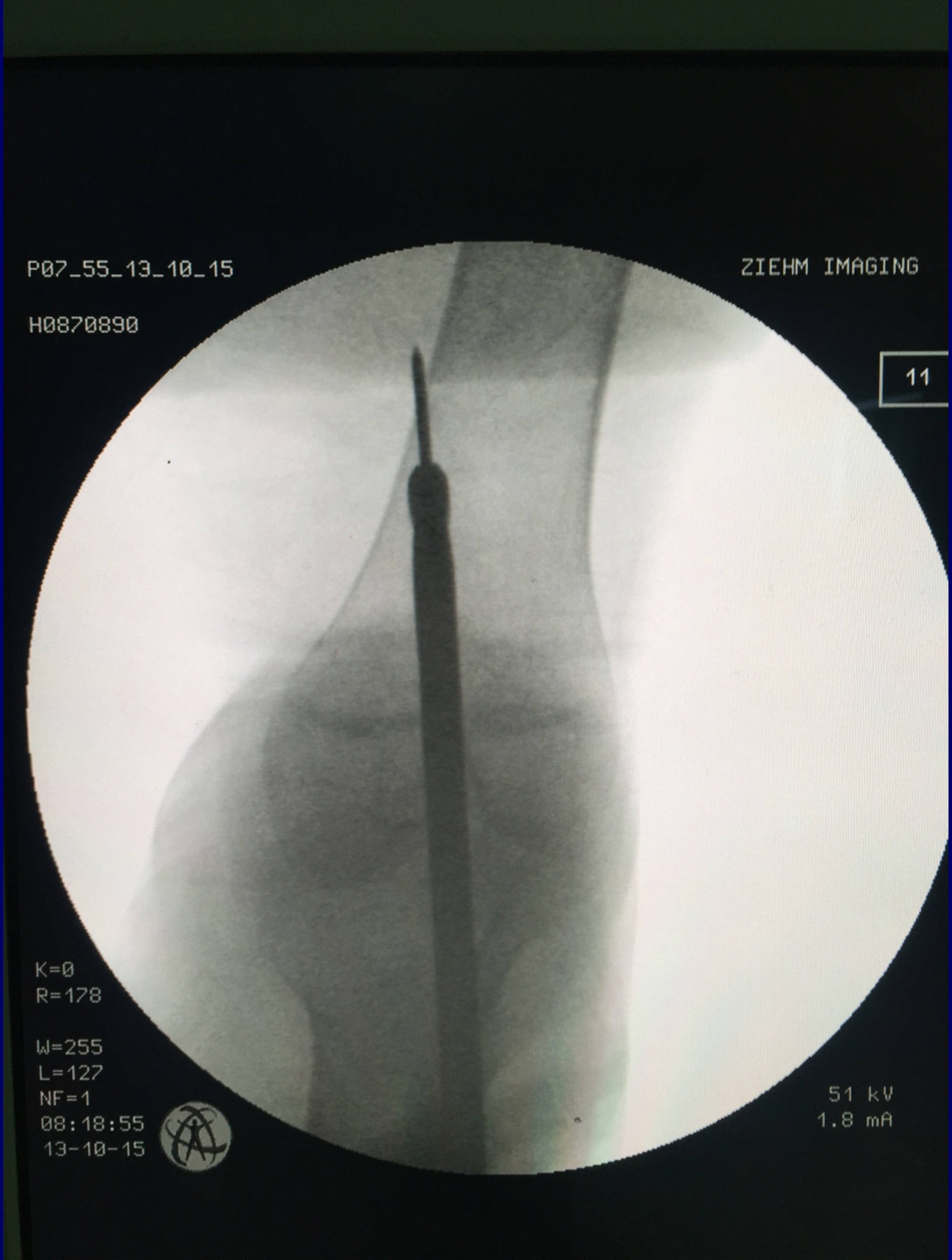
P07_55_13_10_15
ZIEHM IMAGING
H0870890
11
K=0
R=178
W=255
L=127
NF=1
08:18:55
13-10-15
51 kV
1.8 mA

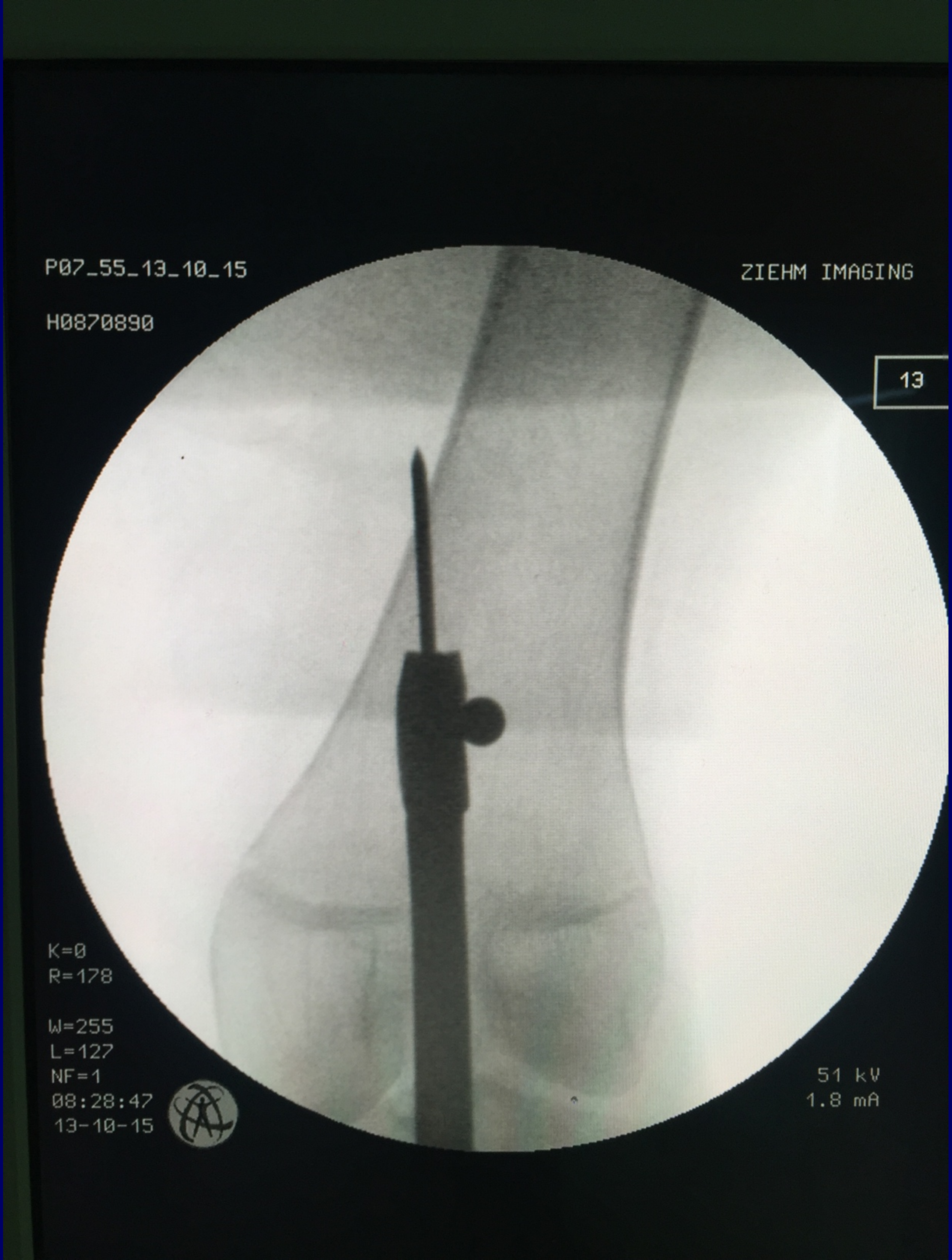
P07_55_13_10_15
H0870890
ZIEHM IMAGING
13
K=0
R=178
W=255
L=127
NF=1
08:28:47
13-10-15
51 kV
1.8 mA

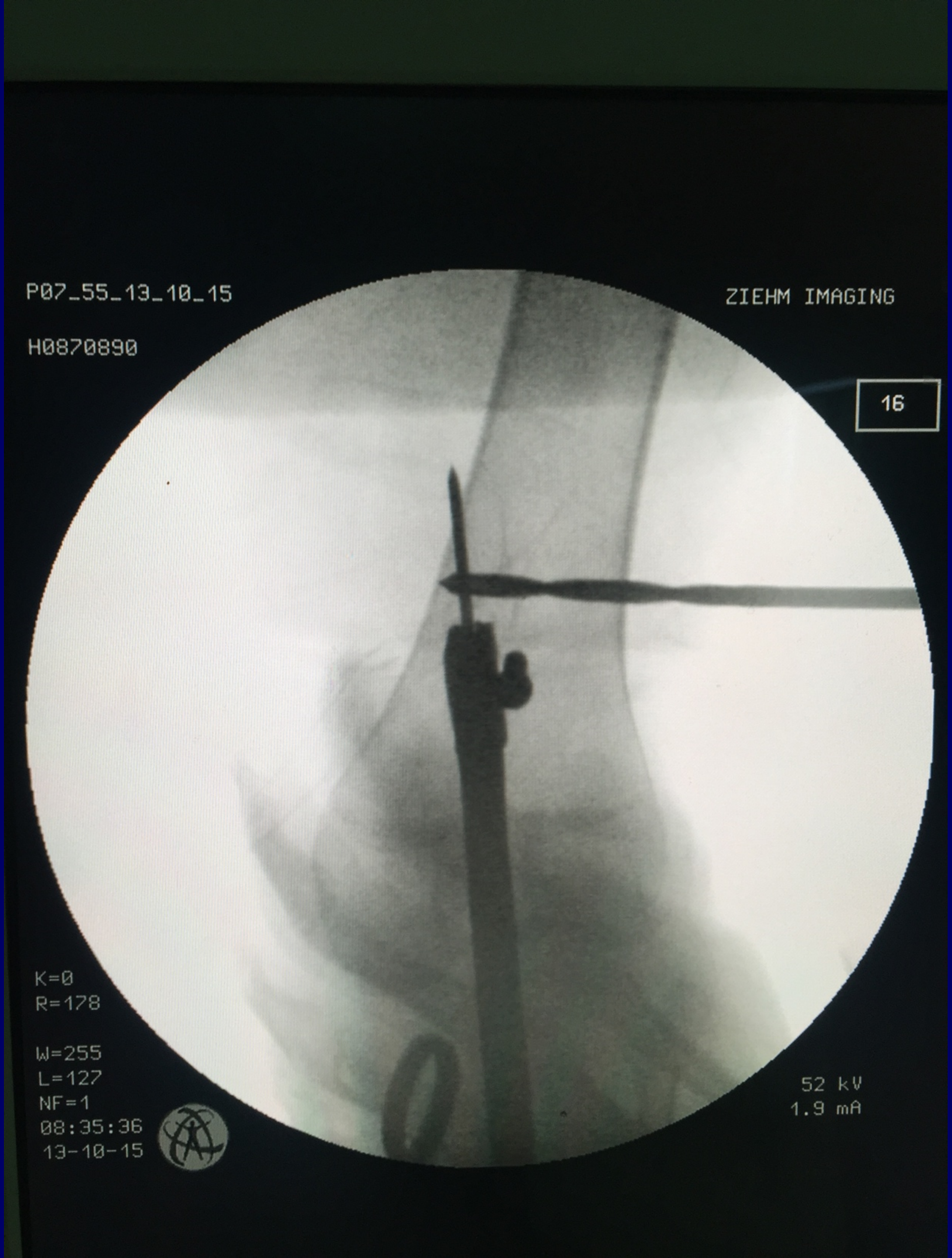
P07_55_13_10_15
H0870890
ZIEHM IMAGING
16
K=0
R=178
W=255
L=127
NF=1
08:35:36
13-10-15
52 kV
1.9 mA

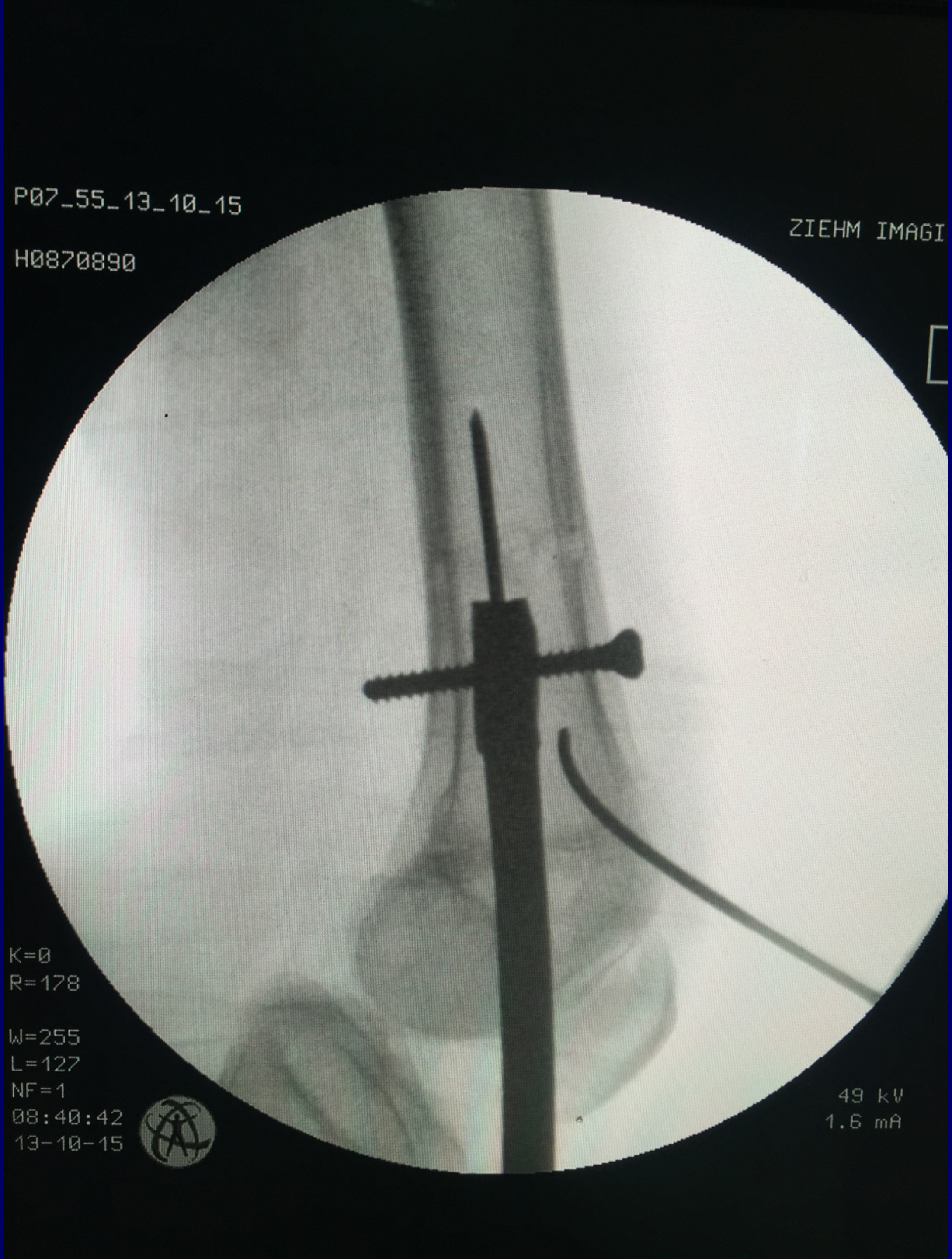
P07_55_13_10_15
H0870890
ZIEHM IMAGI
K=0
R=178
W=255
L=127
NF=1
08:40:42
13-10-15
49 kV
1.6 mA

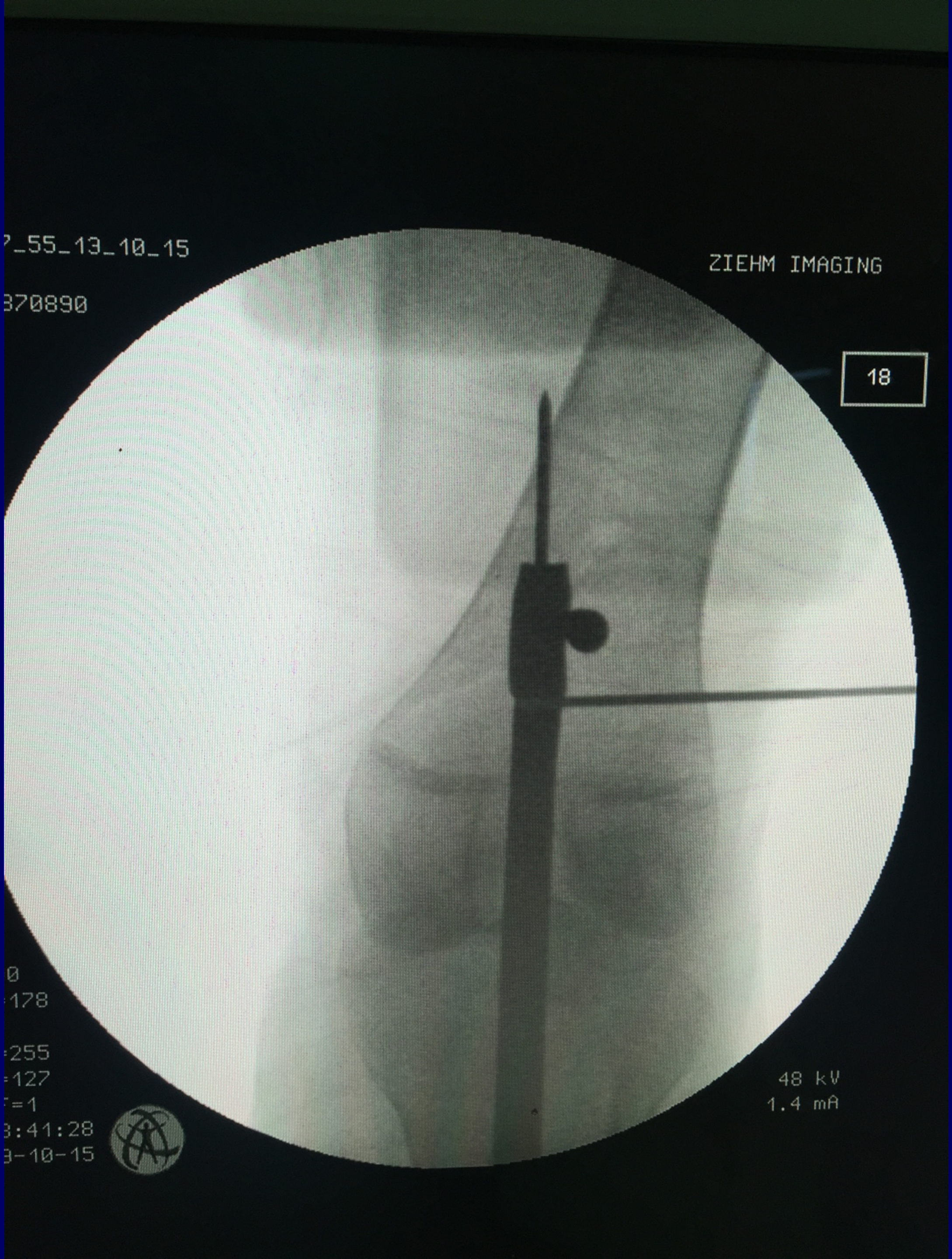
ZIEHM IMAGING
18
48 kV
1.4 mA

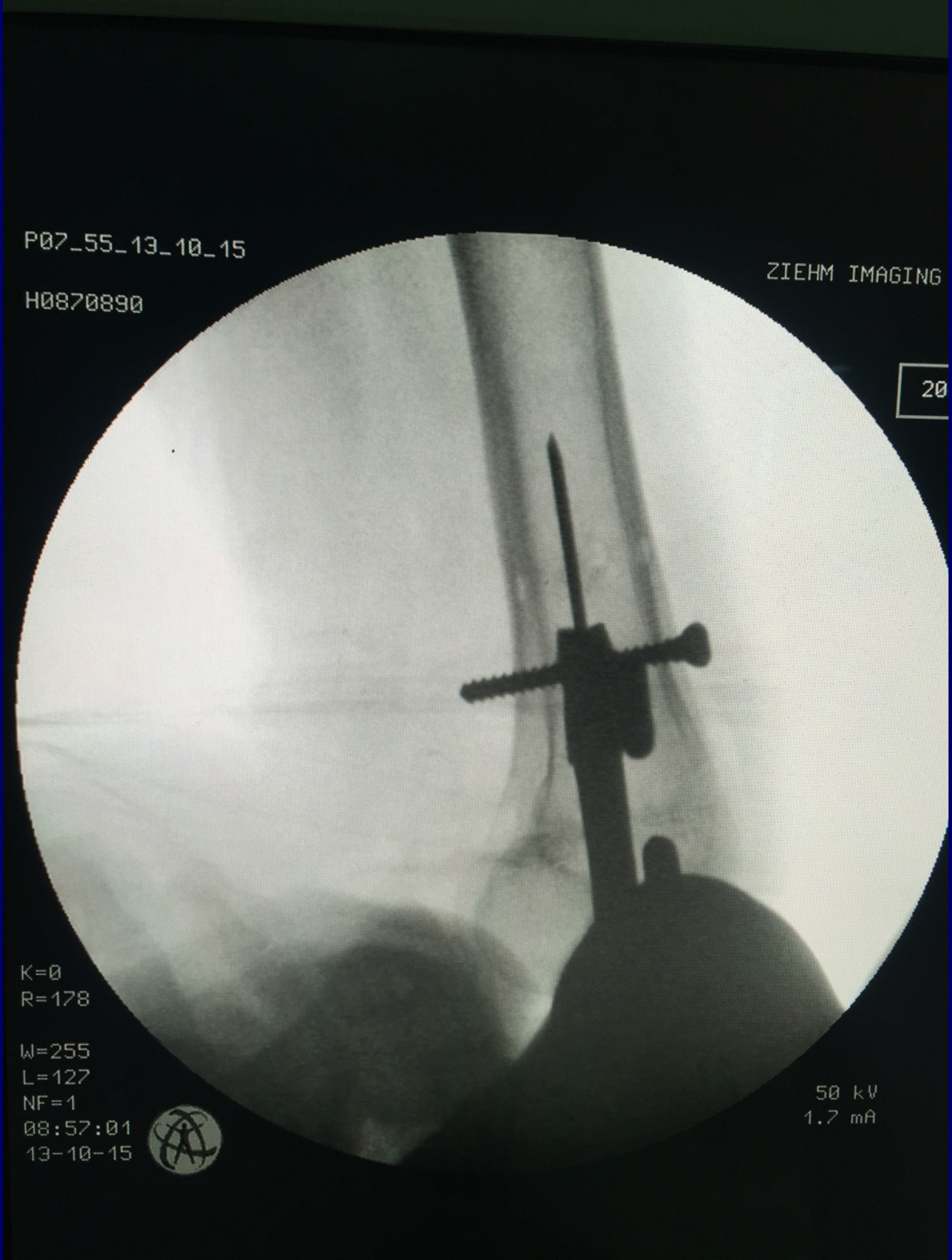
P07_55_13_10_15
H0870890
ZIEHM IMAGING
20
K=0
R=178
W=255
L=127
NF=1
08:57:01
13-10-15
50 kV
1.7 mA

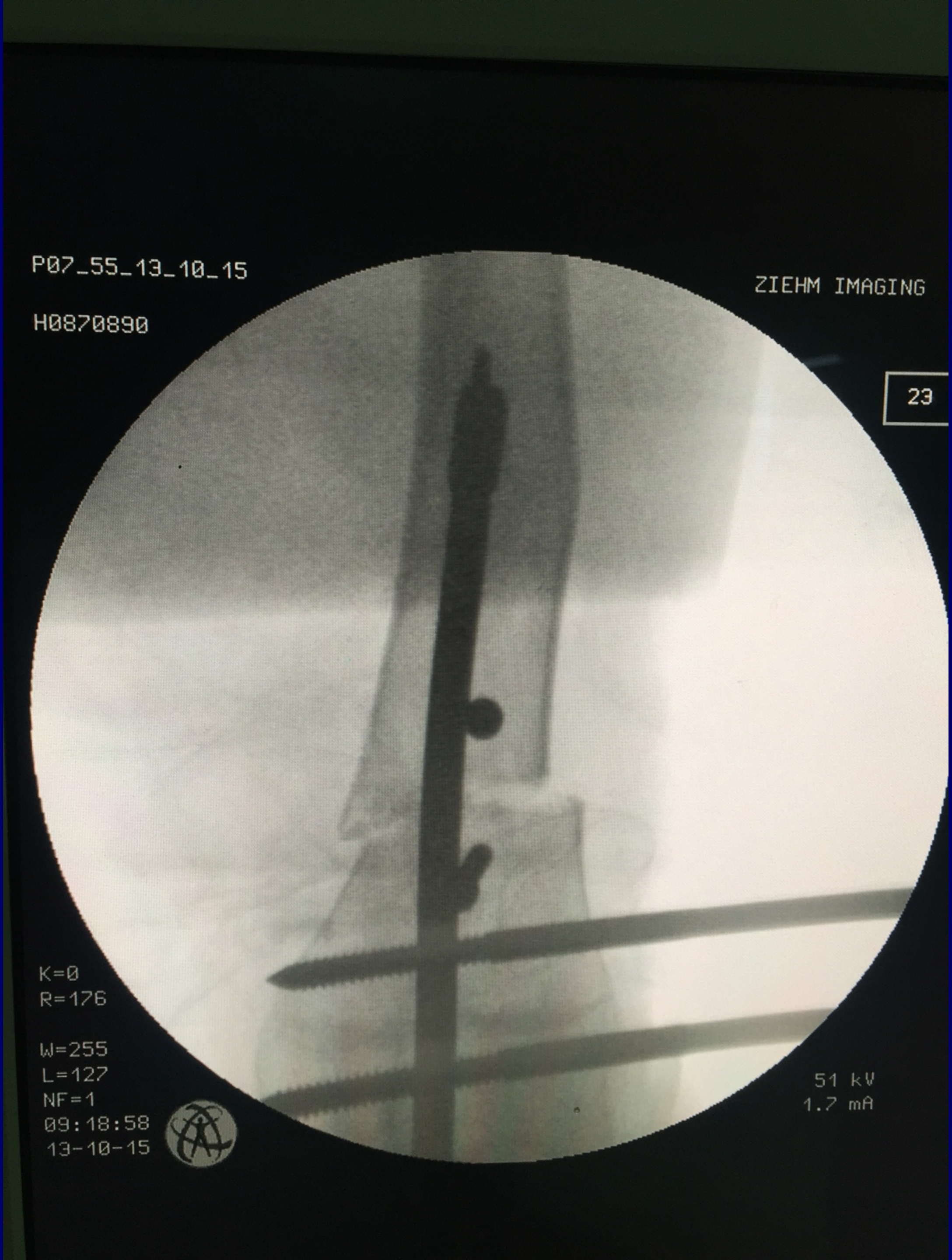

P07_55_13_10_15
H0870890
ZIEHM IMAGING
23
K=0
R=176
W=255
L=127
NF=1
09:18:58
13-10-15
51 kV
1.7 mA

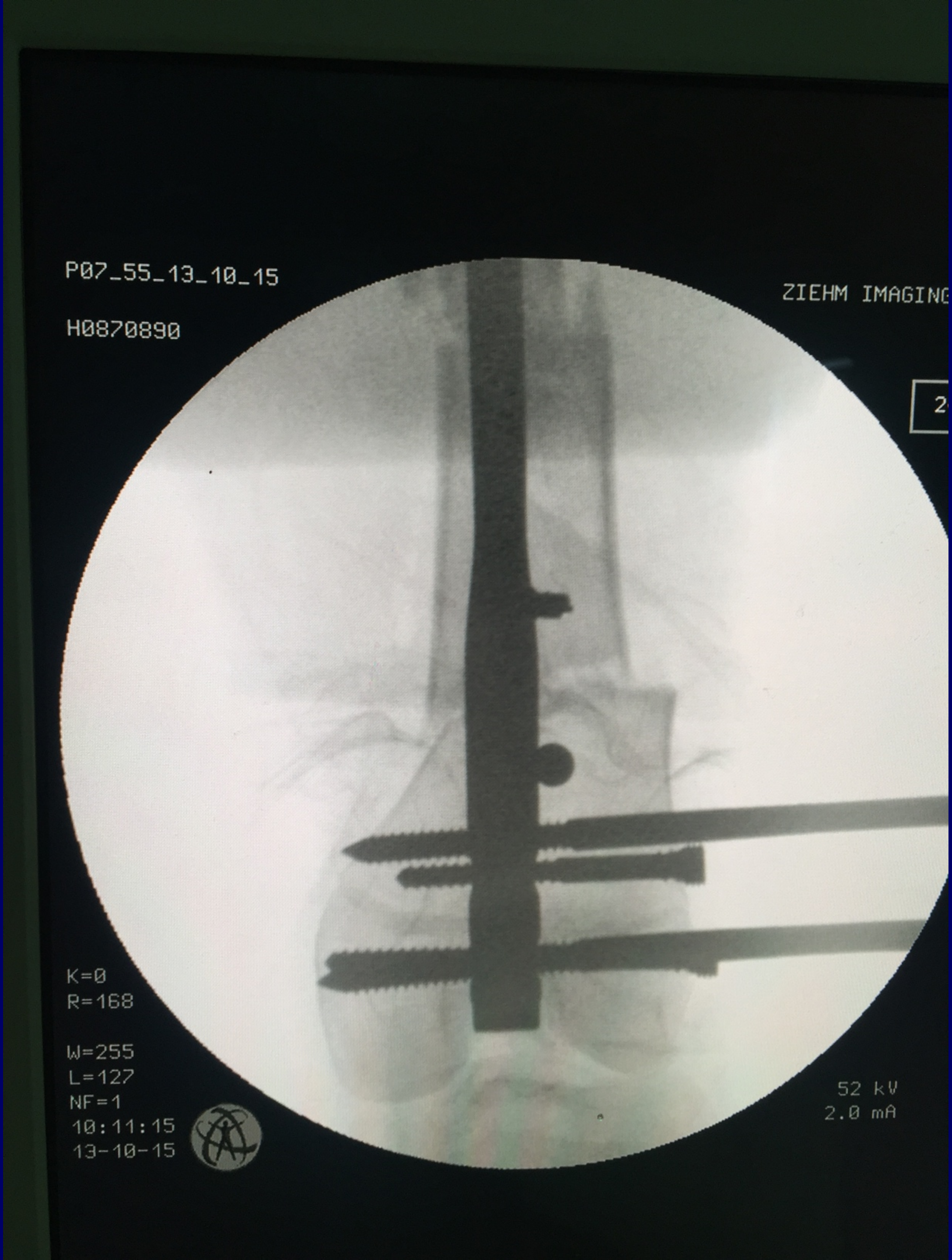

P07_55_13_10_15
H0870890
ZIEHM IMAGING
K=0
R=168
W=255
L=127
NF=1
10:11:15
13-10-15
52 kV
2.0 mA

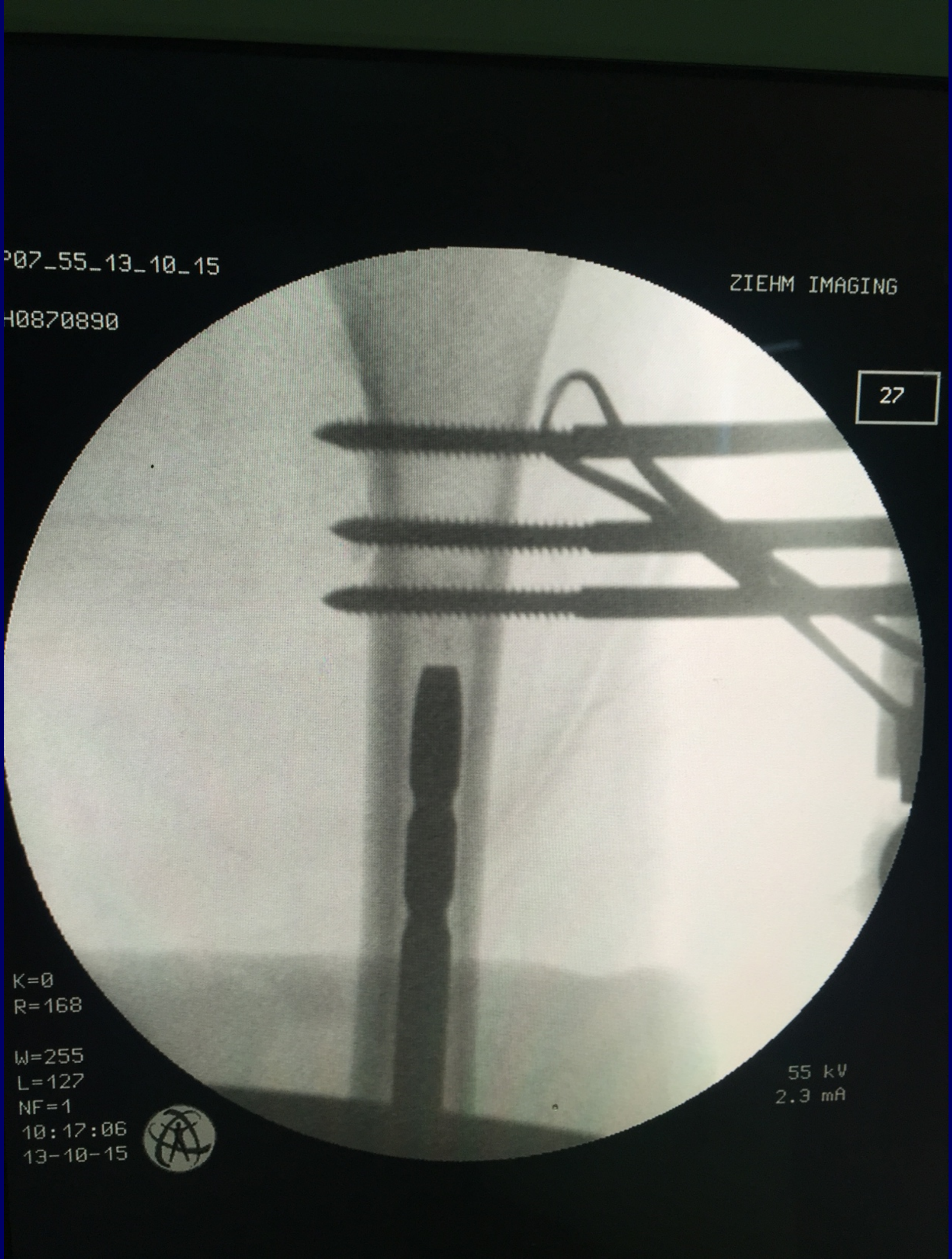

P07_55_13_10_15
H0870890
ZIEHM IMAGING
27
K=0
R=168
W=255
L=127
NF=1
10:17:06
13-10-15
55 kV
2.3 mA

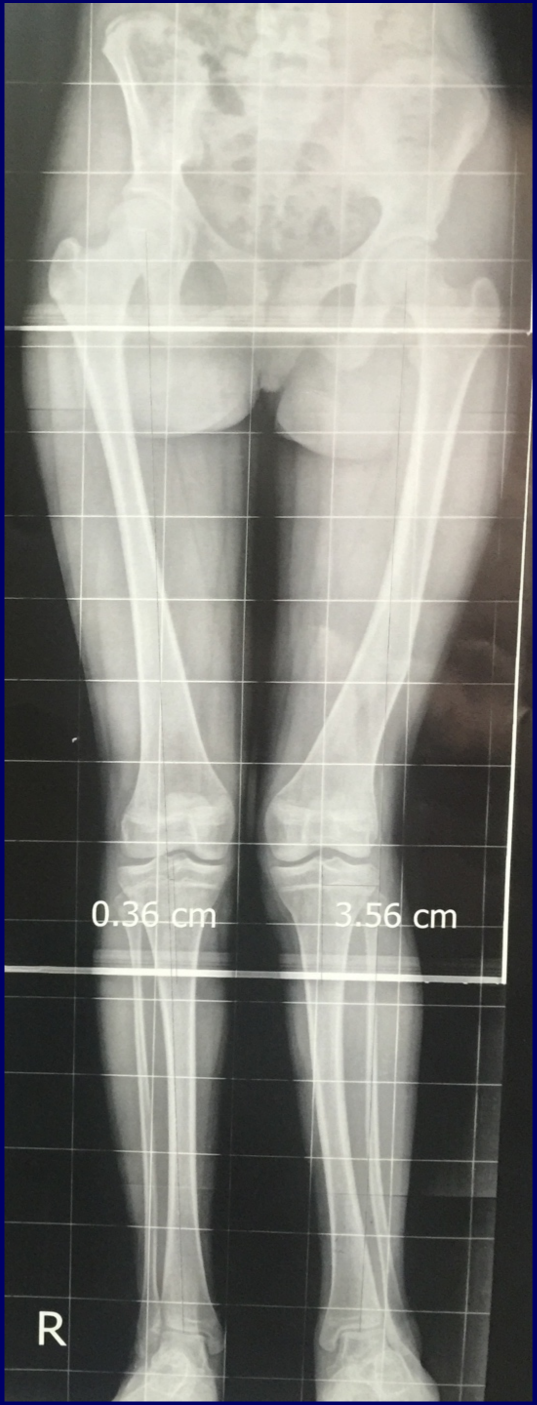
0.36 cm
3.56 cm
R

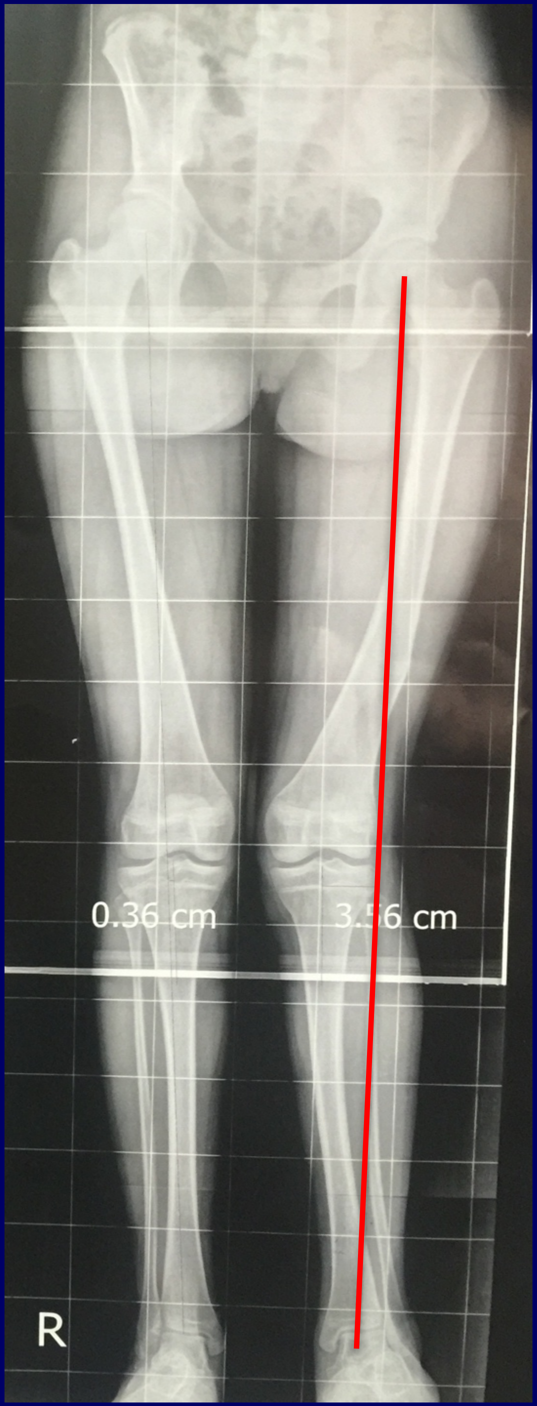
0.36 cm
3.56 cm
R

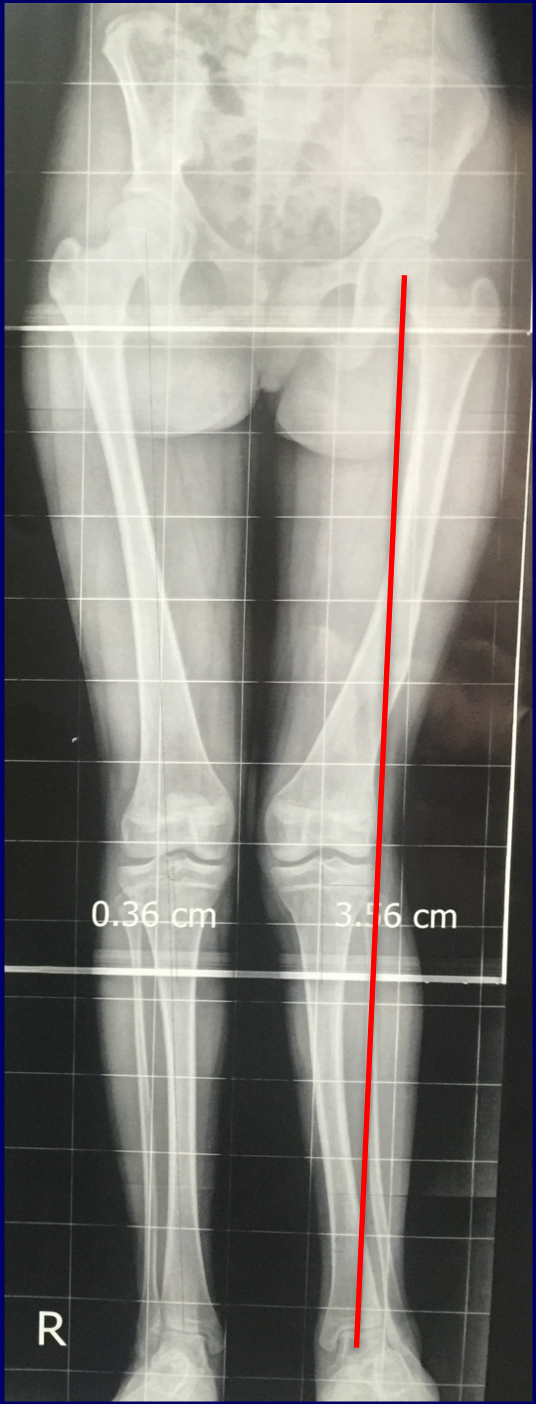
0.36 cm
3.56 cm
R

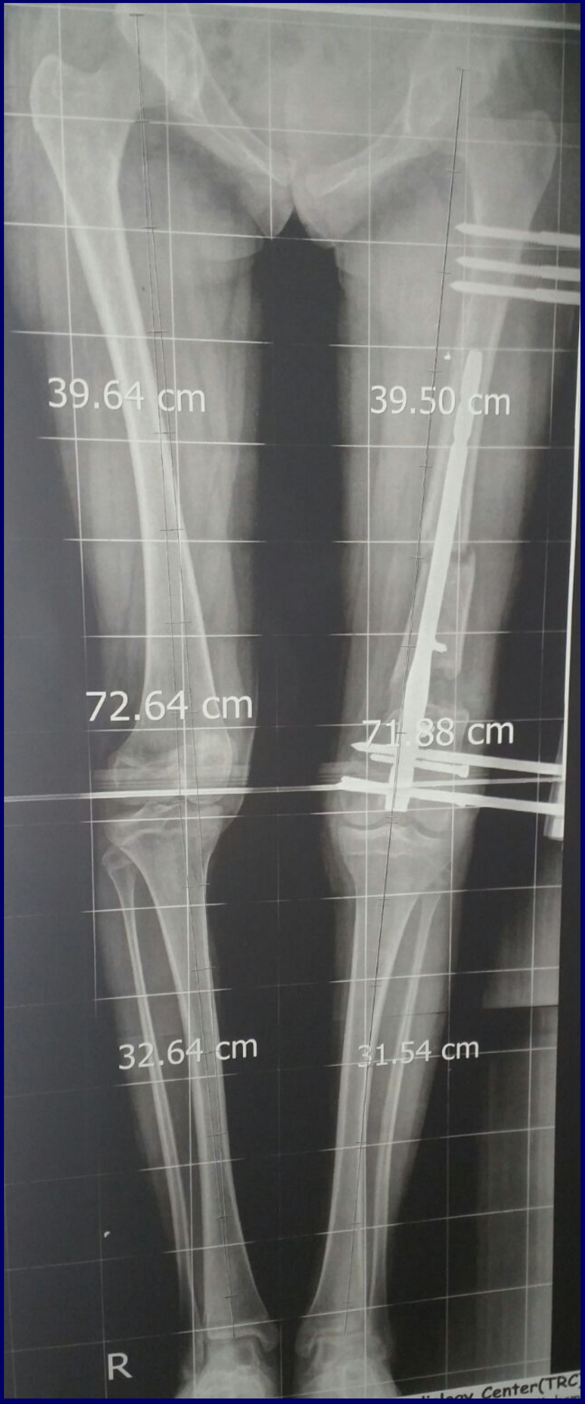
39.64 cm
39.50 cm
72.64 cm
71.88 cm
32.64 cm
31.54 cm
R

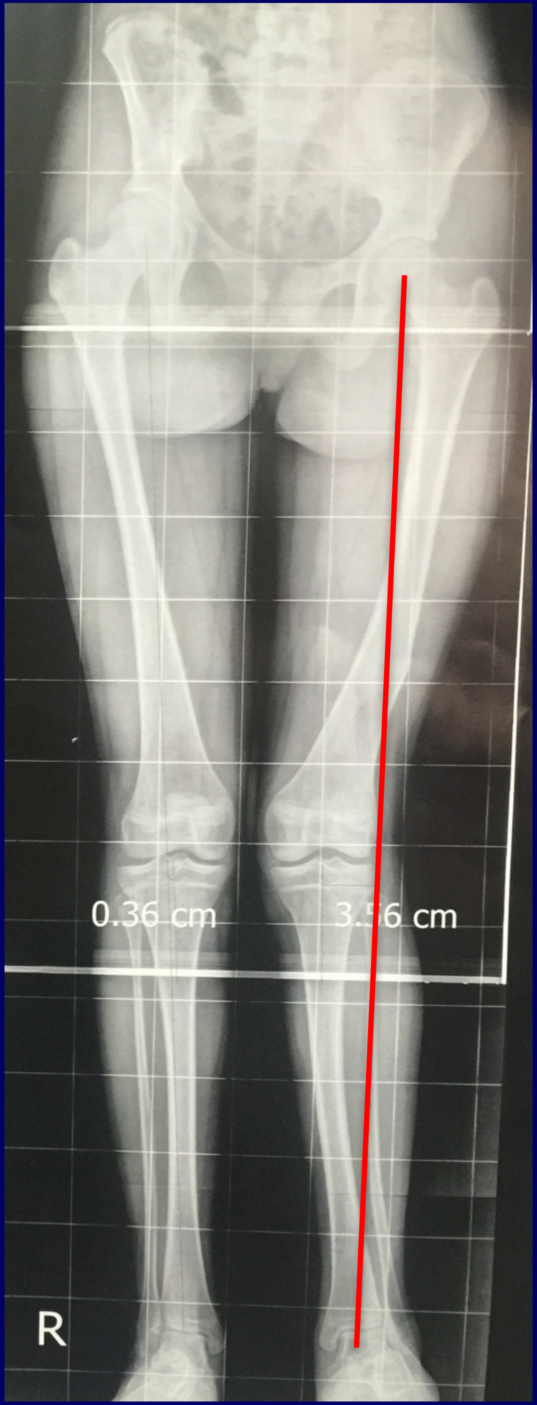
0.36 cm
R

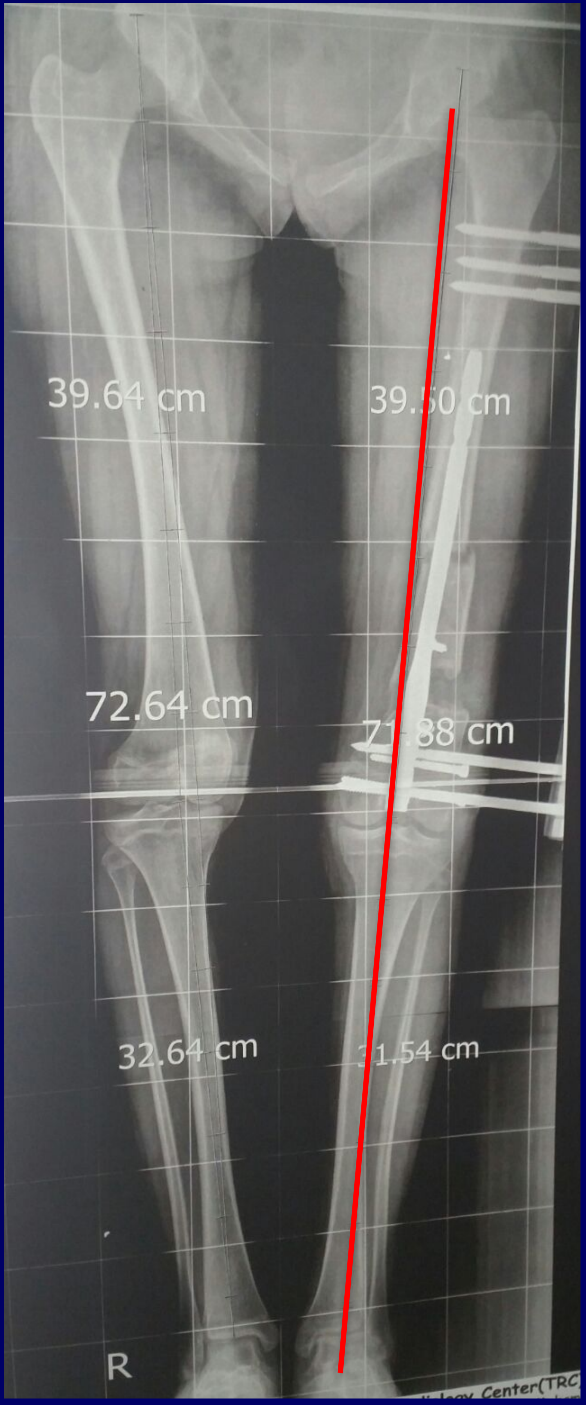
39.64 cm
72.64 cm
32.64 cm
R

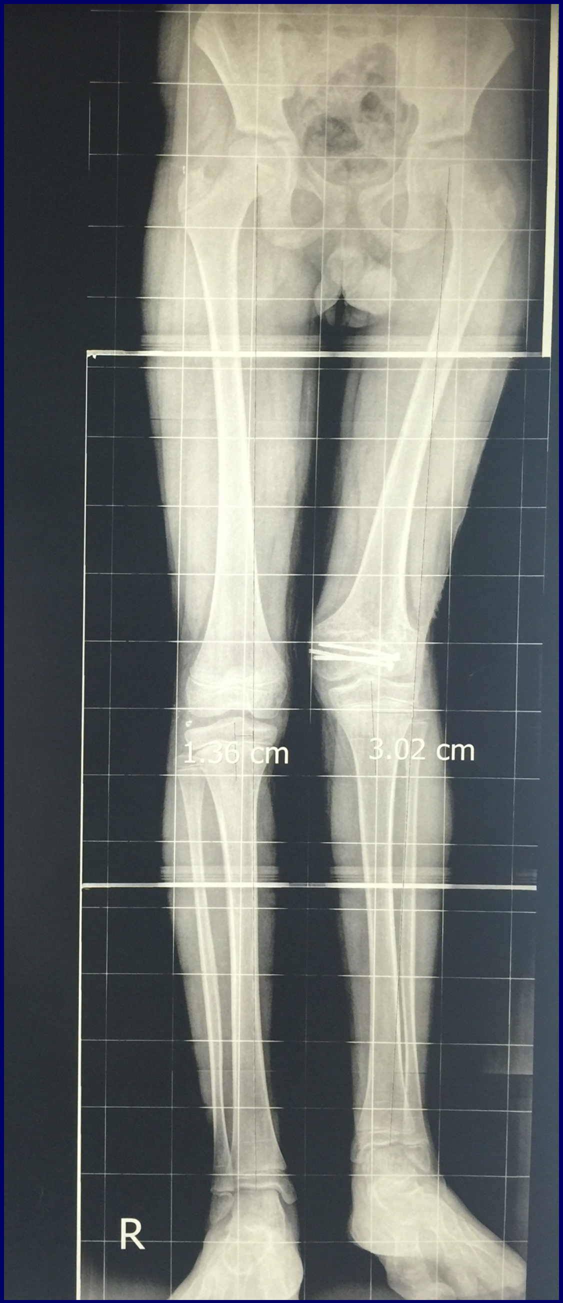
1.36 cm
3.02 cm
R

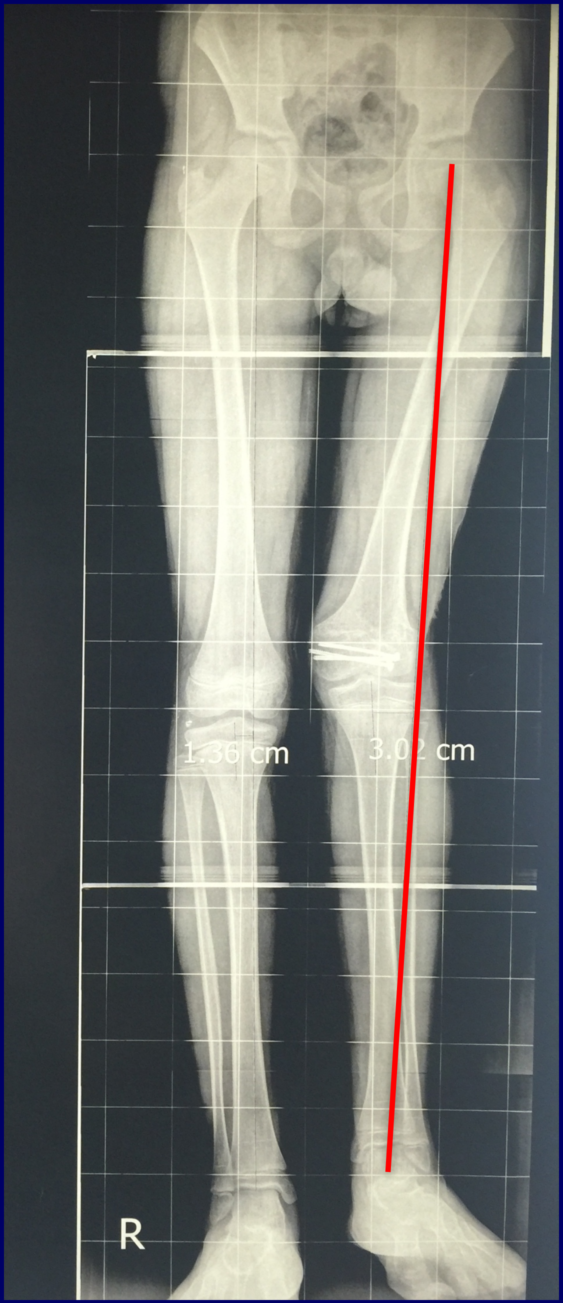
1.36 cm
3.02 cm
R

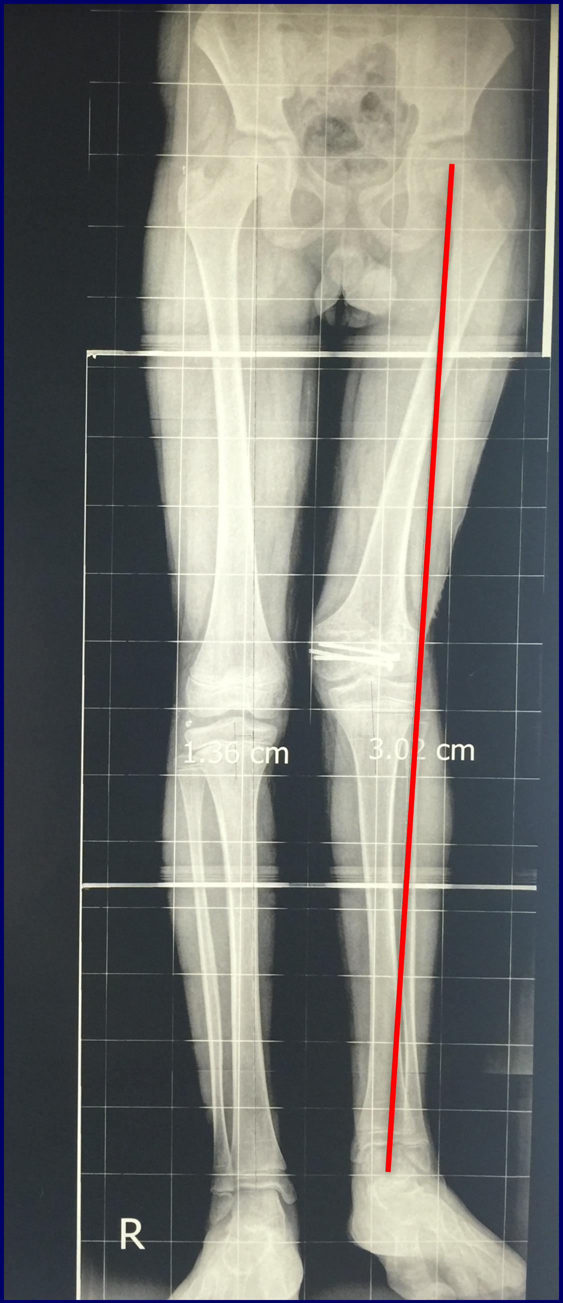
1.36 cm
3.02 cm
R

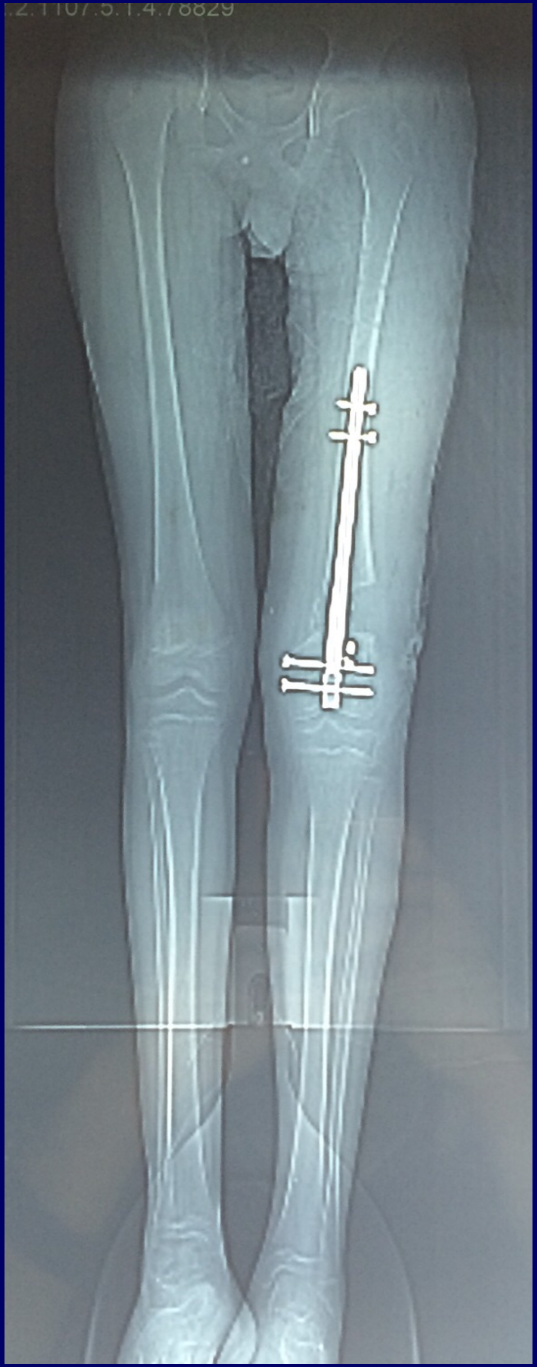

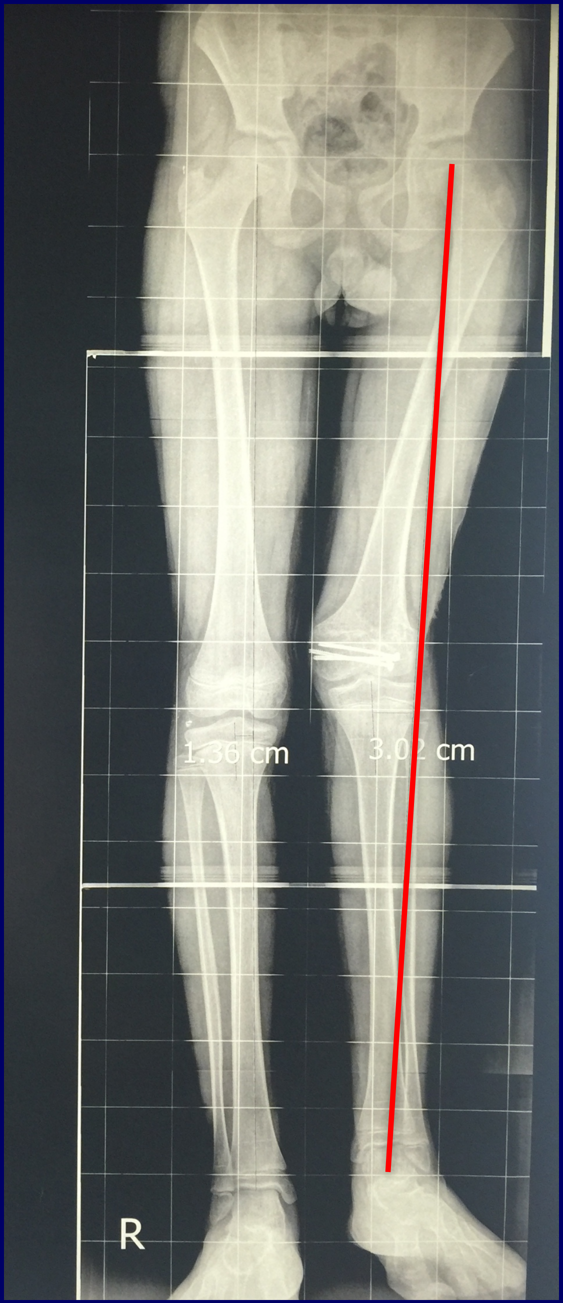
1.36 cm
3.02 cm
R

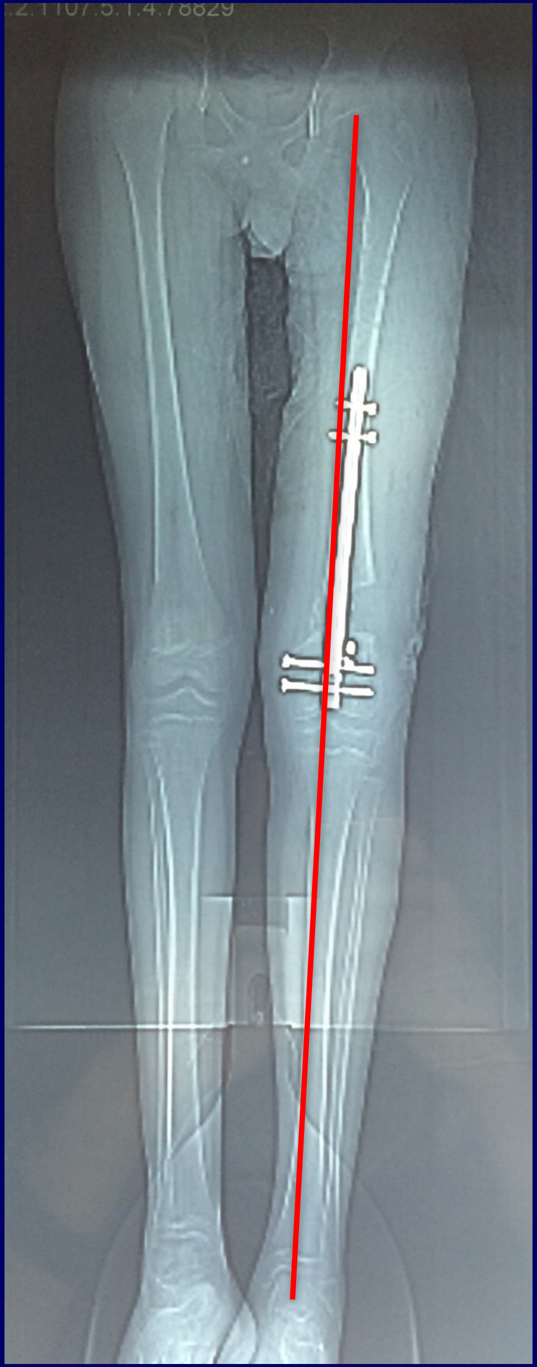
.2.1107.5.1.4.78829

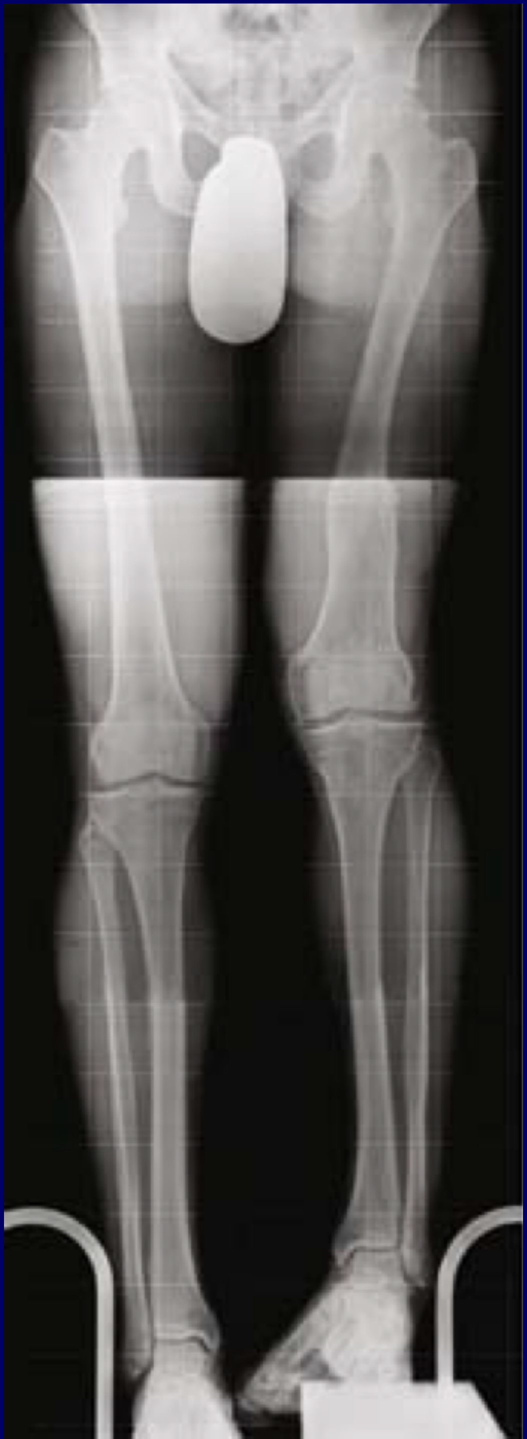

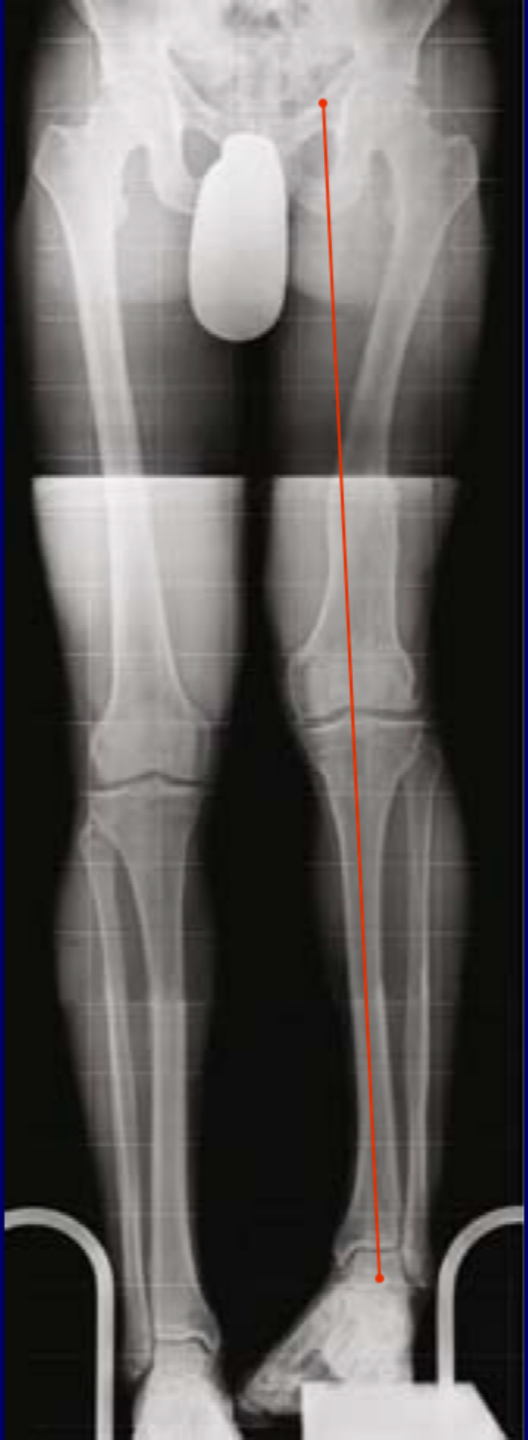

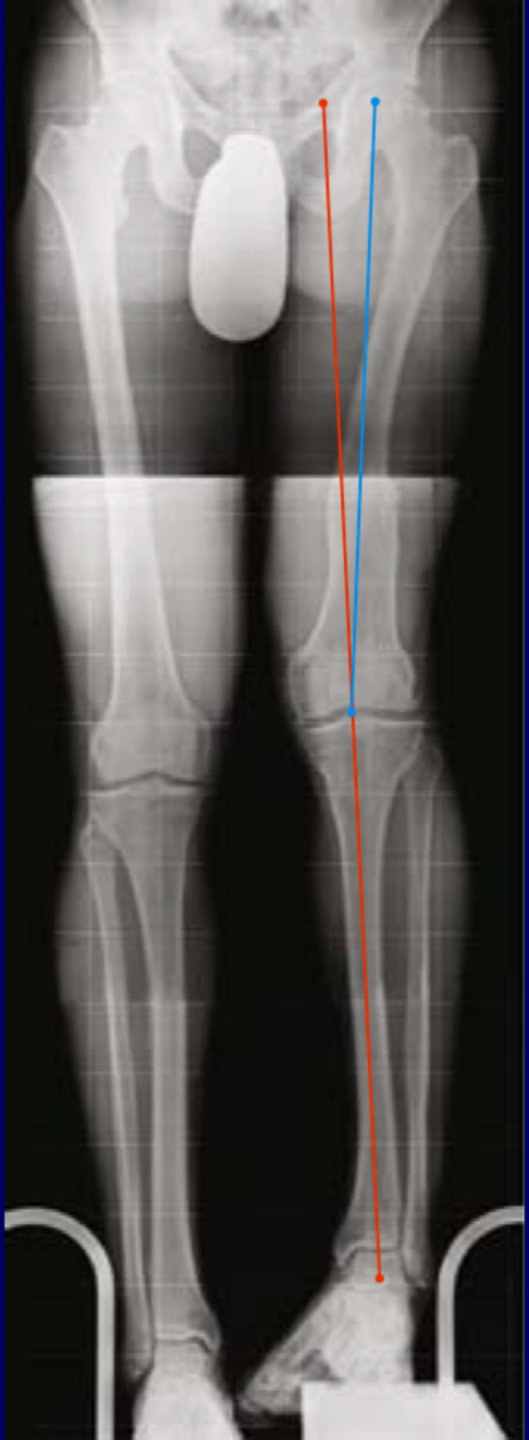

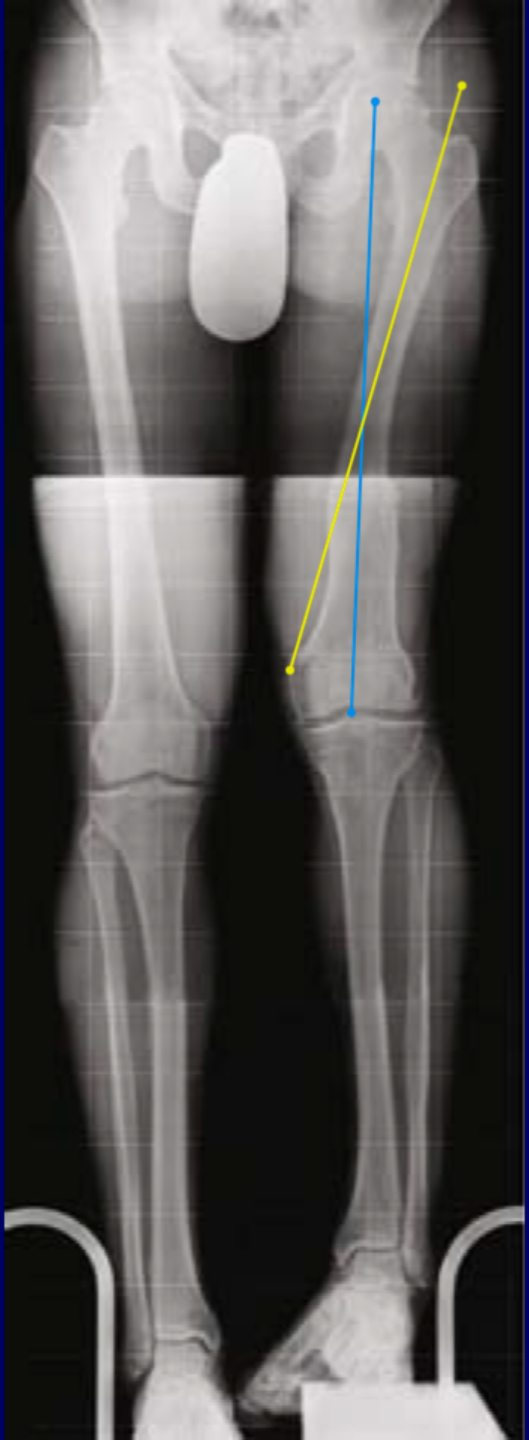

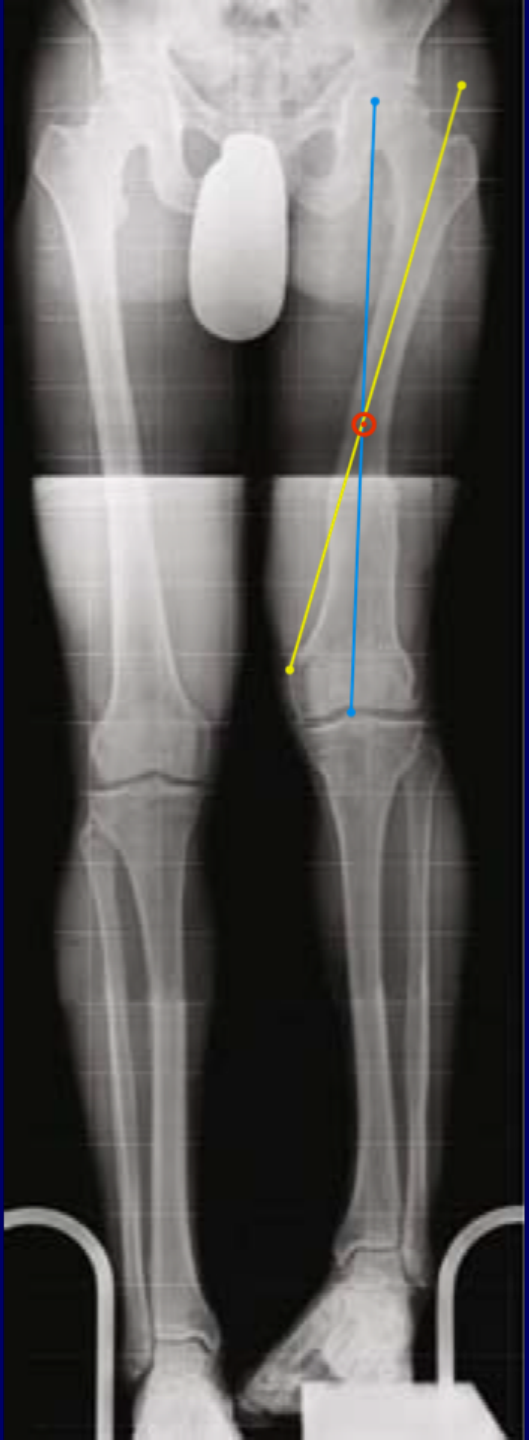

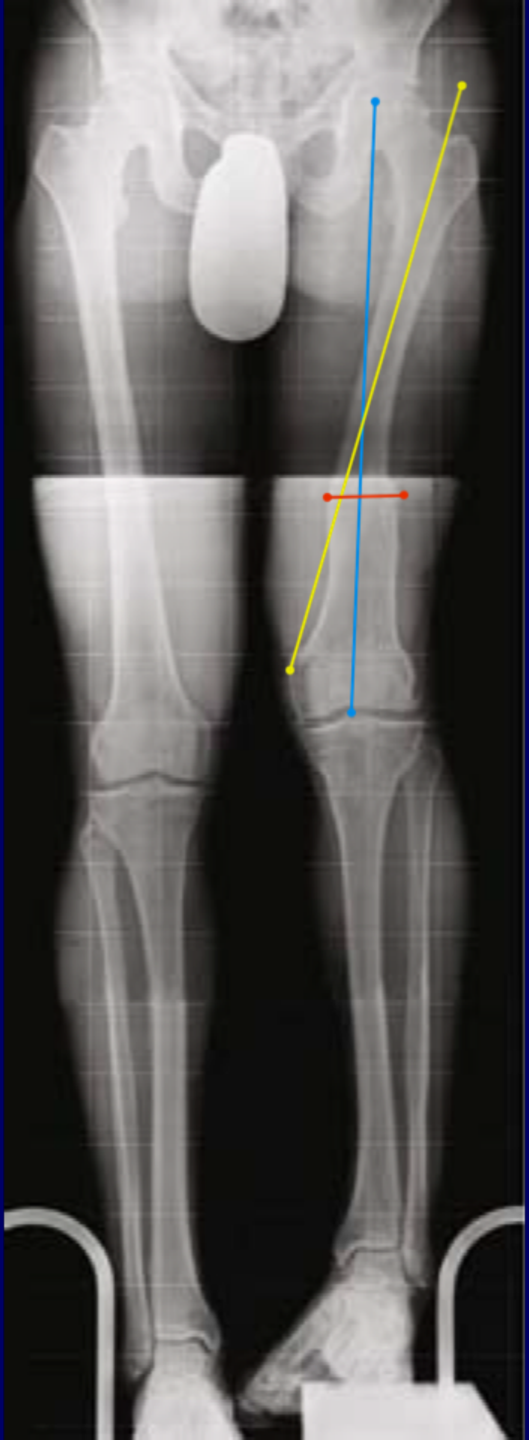

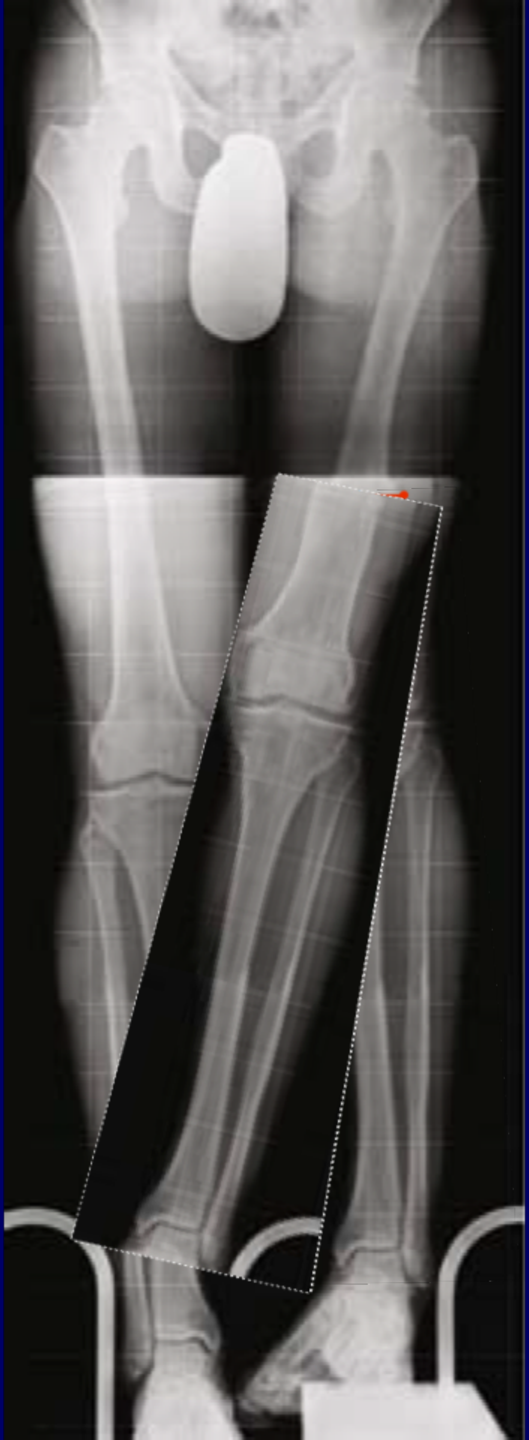

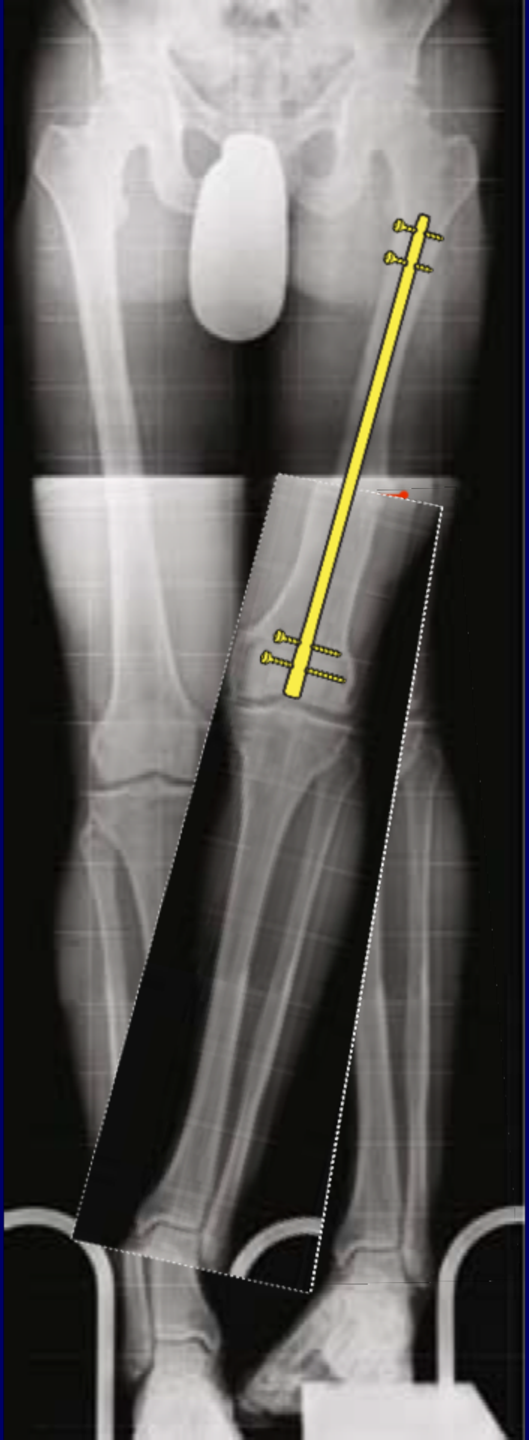

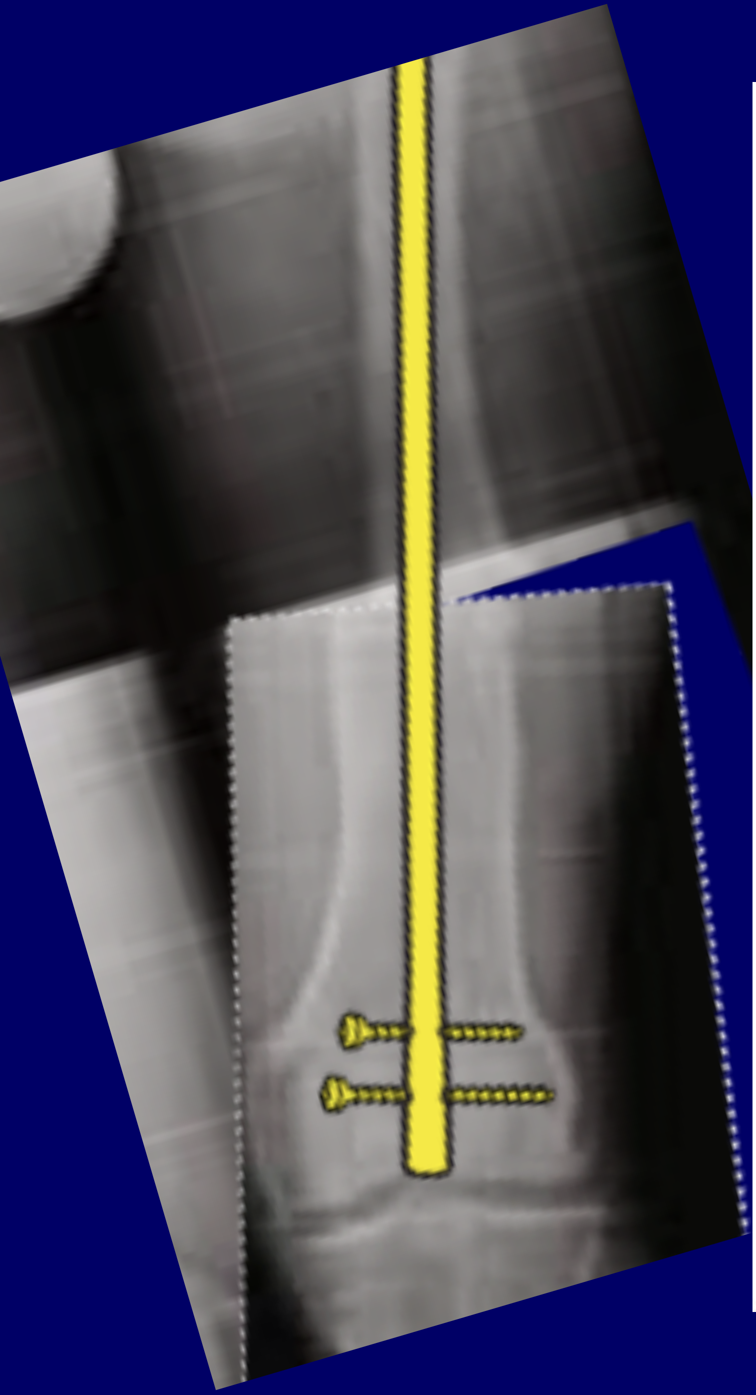

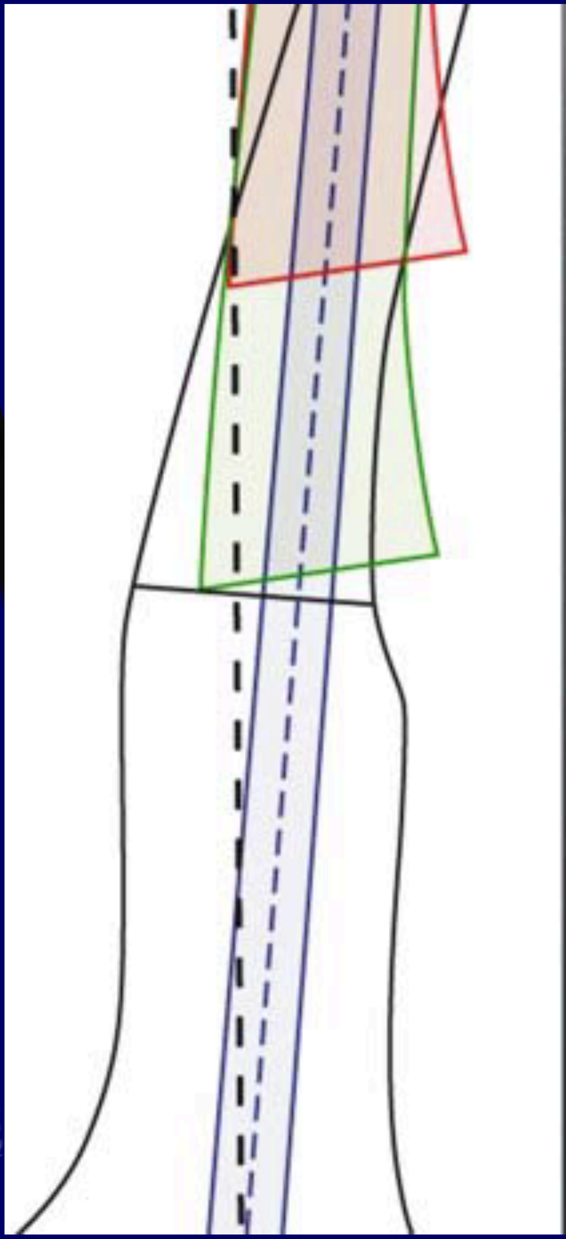

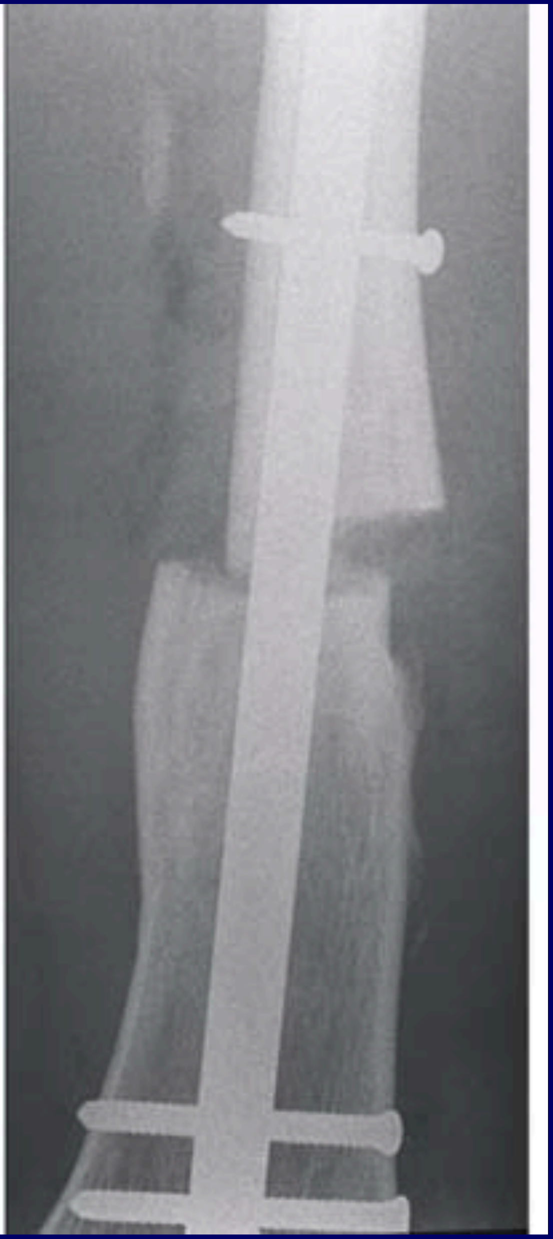

Case Number	Age in years	Sex	Deformity Associated	Deformity Apex	Shortening in cm	Pre-operative aLDFA angle in degrees	Final aLDFA angle in degrees	Pre-operative MAD in cm	Final MAD in cm
1	18	M	None	-	4	82	81	0	0
2	14	M	Varus	Metaphyseal	4	90	82	+3	0
3	20	F	None	-	5	81	80	0	0
4	15	F	Varus	Metaphyseal	5	92	81	+3.4	0
5	14	M	Varus	Metaphyseal	5	90	82	+3.6	0
6	12	M	Valgus	Metaphyseal	4	73	80	-2.2	0
7	16	M	None	-	8	81	80	0	0
8	13	F	Valgus	Metaphyseal	6	75	80	-1.8	0

- In the pre-lengthening condition we can draw a triangle ABC where

- Point A represent the nail entry point at the center of the knee
- Point B represent the center of the femoral head
- Point C represent the piriformis fossa.

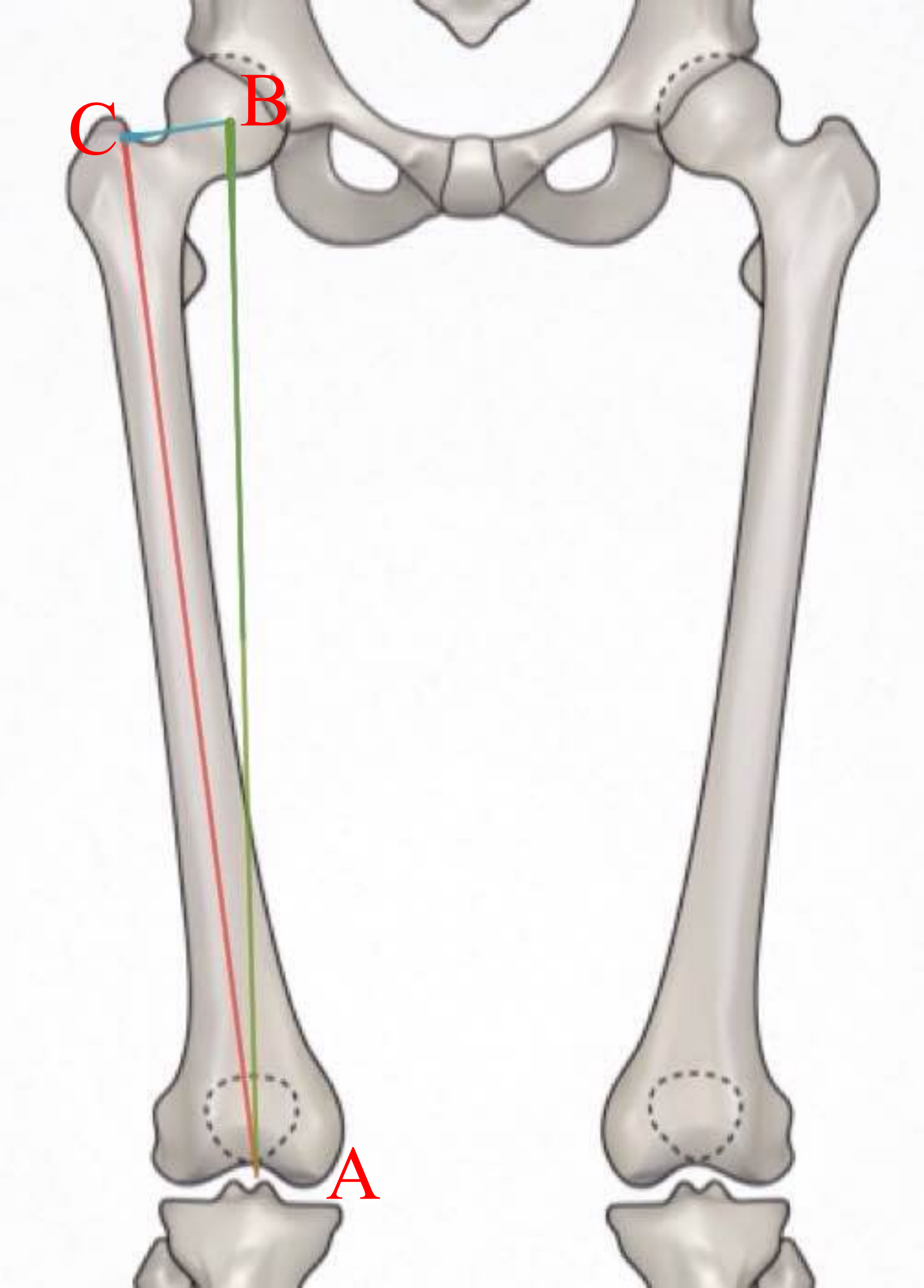

- AB line represents the femoral mechanical axis & we'll refer to its length as "c"
- BC line is the femoral neck & we'll refer to it's length as "a".
- We'll refer to the Femoral Mechanical Anatomical (FMA) angel as alpha "α" (the angle between AB & AC lines)
- We'll refer to the angle between the mid-diaphyseal line of the proximal femoral fragment (AB line) & the line joining the center of the femoral head to the piriformis fossa (BC line) as gamma "γ"

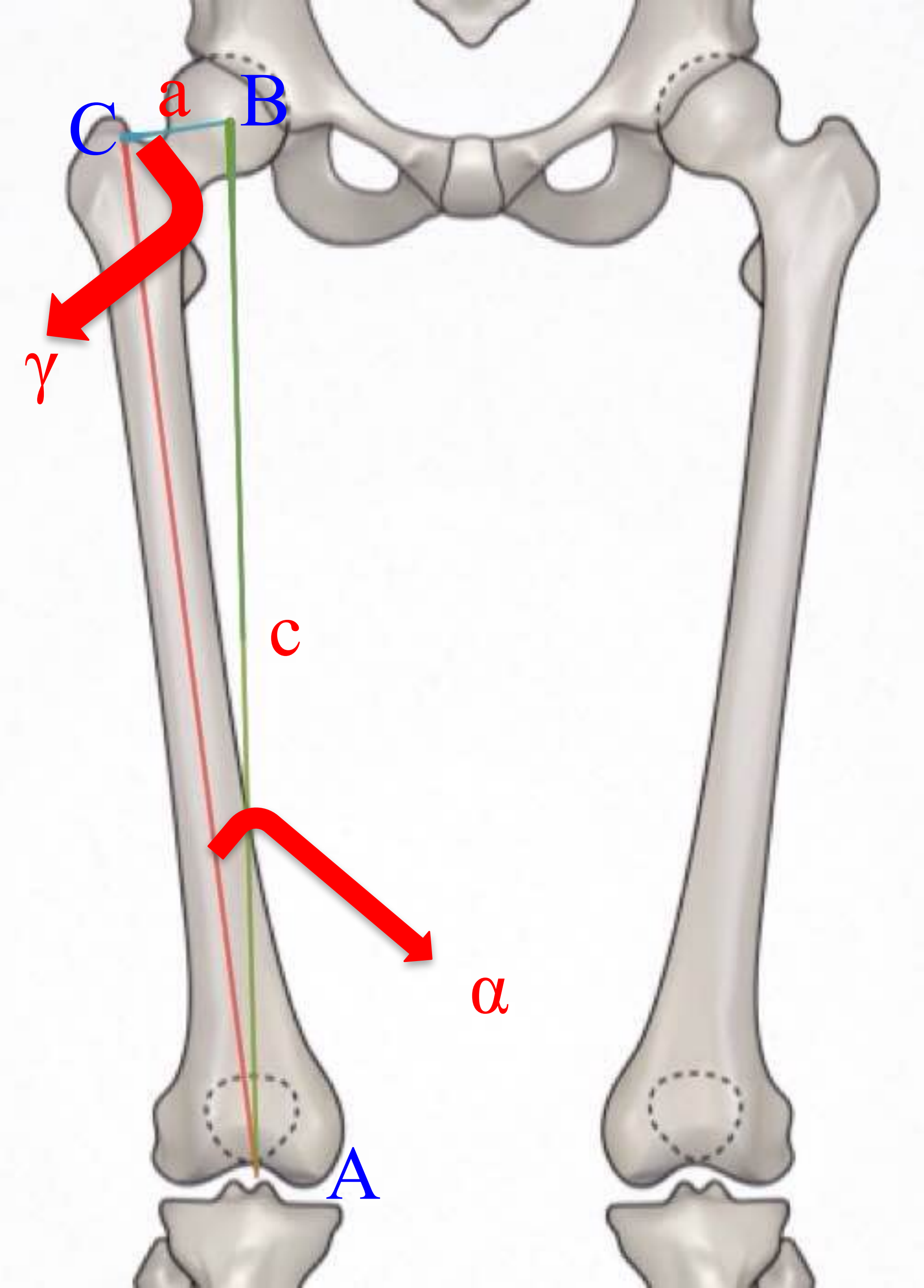

- In the post-lengthening condition we’ll use A, B, C to refer to the same points as before
- we’ll refer to the (new) length of the femoral mechanical axis as “c`”
- the femoral neck length will remain (unchanged) as “a”
- we’ll refer to the (new) Femoral Mechanical Anatomical (FMA) angel as alpha` “α`”
- angle “γ” will remain (unchanged) because the head center, piriformis & the proximal femoral fragment retain their anatomical relationship
- In this triangle we only need to measure the distance “c`” (c`= c + amount of lengthening).

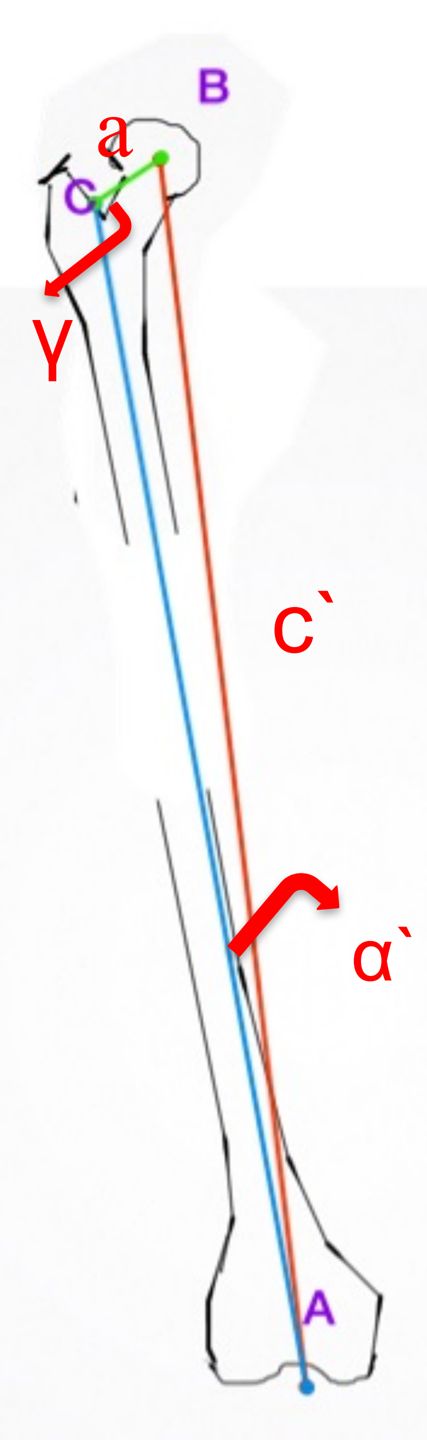

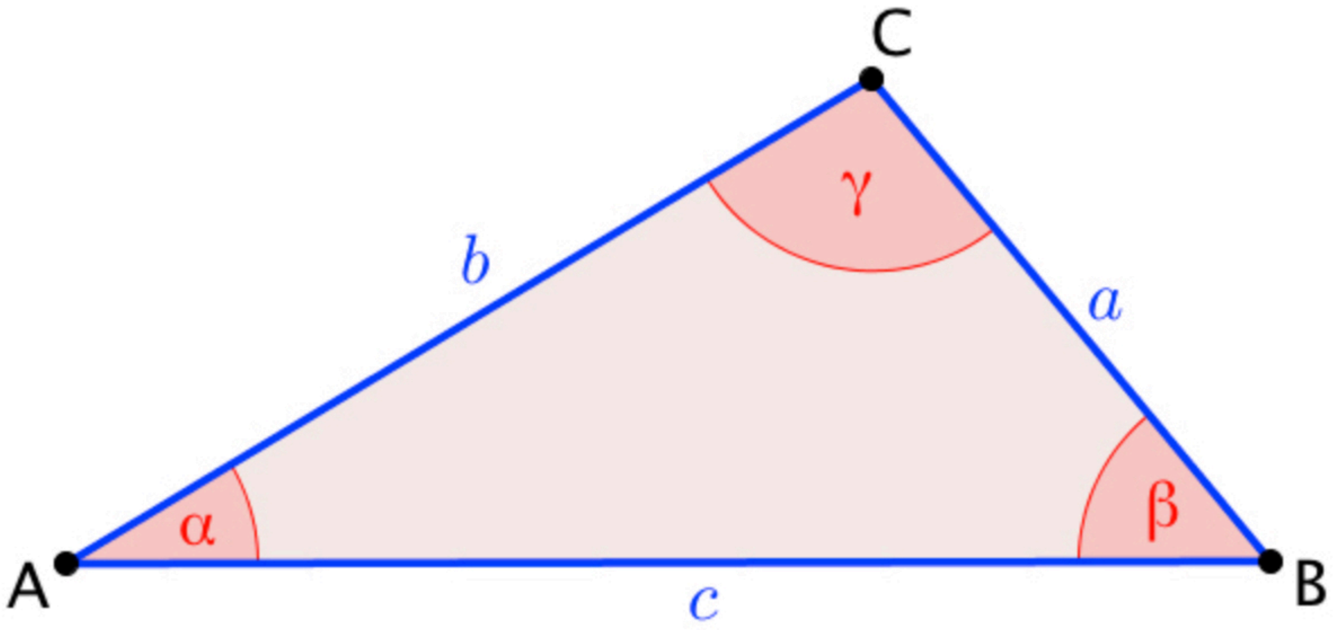

$$\frac{a}{sin\alpha} = \frac{b}{sin\beta} = \frac{c}{sin\gamma}$$

Law of Sines

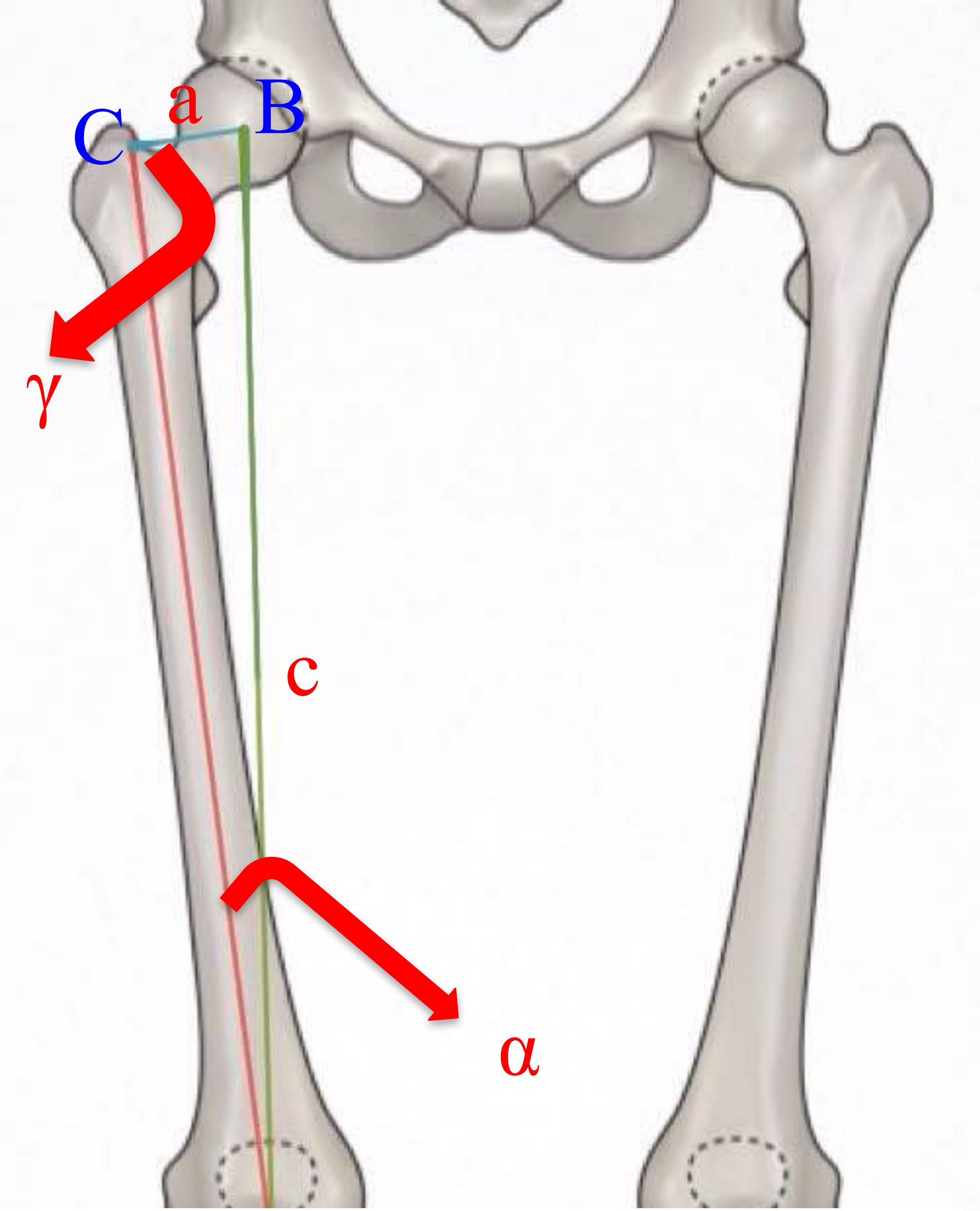

For pre-lengthening triangle: $\frac{\sin\alpha}{a} = \frac{\sin\gamma}{c}$ ➔ $a * \sin\gamma = c * \sin\alpha$

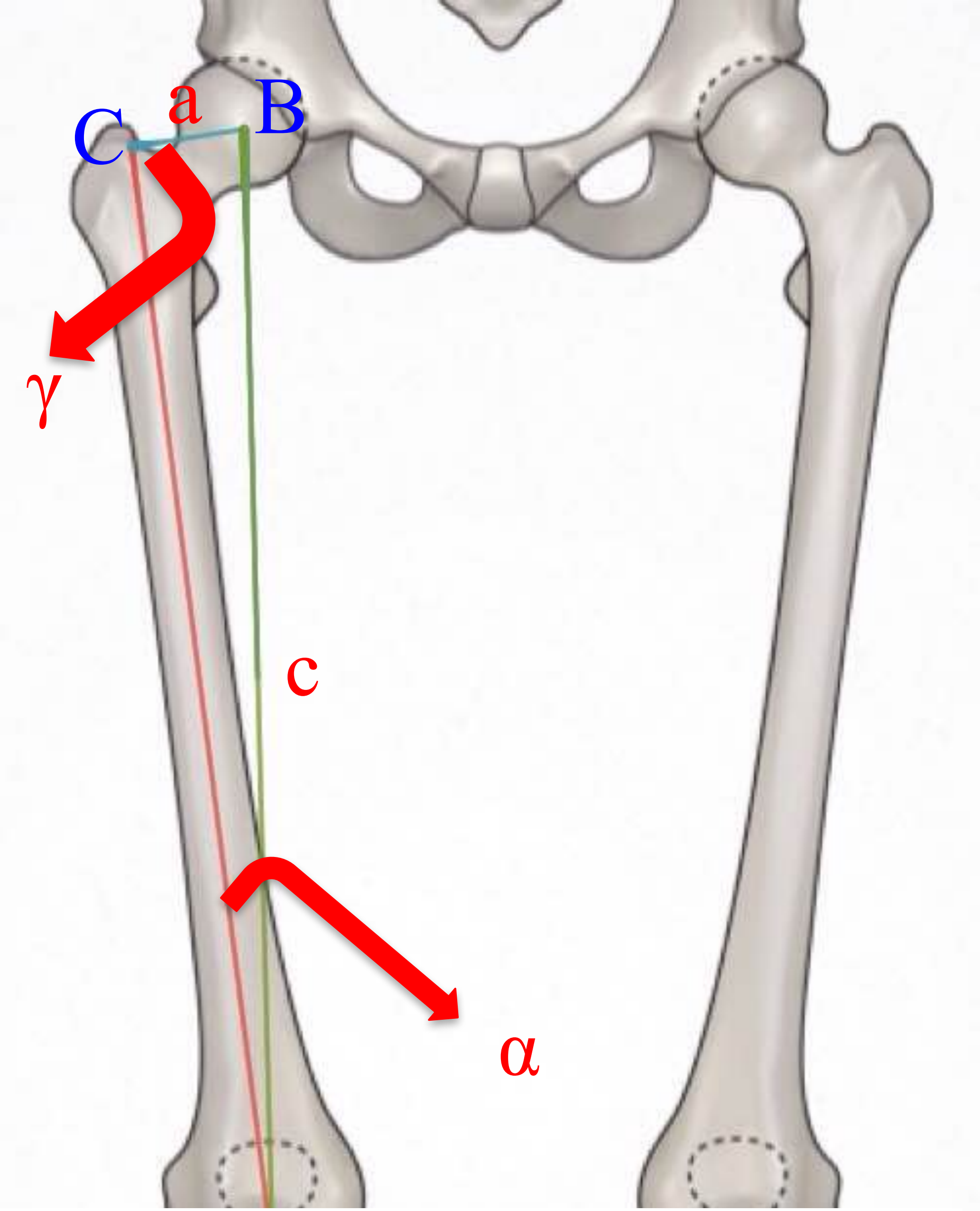

For pre-lengthening triangle: $\frac{\sin\alpha}{a} = \frac{\sin\gamma}{c}$ ➔ $a * \sin\gamma = c * \sin\alpha$

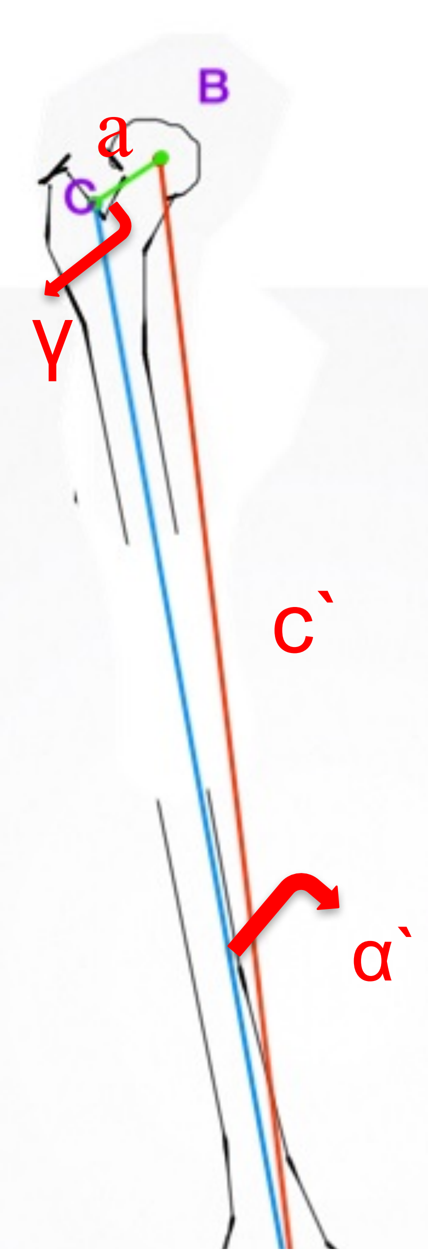

For post-lengthening triangle: $\frac{\sin\alpha`}{a} = \frac{\sin\gamma}{c`} \rightarrow a * \sin\gamma = c` * \sin\alpha`$

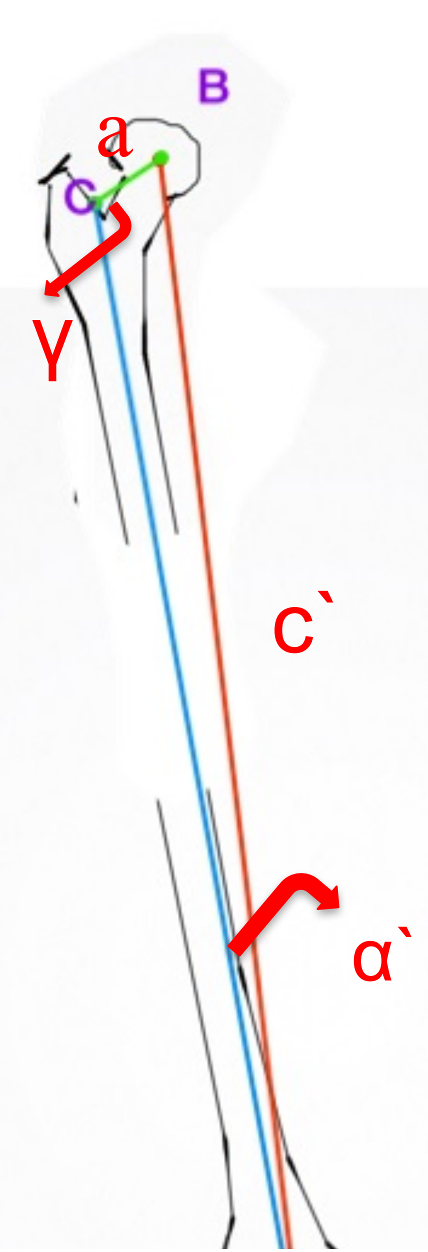

For post-lengthening triangle: $\frac{\sin\alpha`}{a} = \frac{\sin\gamma}{c`}$ ➔ $a * \sin\gamma = c` * \sin\alpha`$

For pre-lengthening triangle: $\frac{\sin\alpha}{a} = \frac{\sin\gamma}{c}$ ➔ $a * \sin\gamma = c * \sin\alpha$

For post-lengthening triangle: $\frac{\sin\alpha`}{a} = \frac{\sin\gamma}{c`}$ ➔ $a * \sin\gamma = c` * \sin\alpha`$

For pre-lengthening triangle: $\frac{\sin\alpha}{a} = \frac{\sin\gamma}{c}$ ➔ $a * \sin\gamma = c * \sin\alpha$

For post-lengthening triangle: $\frac{\sin\alpha`}{a} = \frac{\sin\gamma}{c`}$ ➔ $a * \sin\gamma = c` * \sin\alpha`$

For pre-lengthening triangle: $\frac{\sin\alpha}{a} = \frac{\sin\gamma}{c}$ ➔ $a * \sin\gamma = c * \sin\alpha$

For post-lengthening triangle: $\frac{\sin\alpha`}{a} = \frac{\sin\gamma}{c`}$ ➔ $a * \sin\gamma = c` * \sin\alpha`$

Thus $c * \sin\alpha = c` * \sin\alpha`$ ➔ **Sin α` = c/ c` X Sinα**

$$\mathbf{Sin}\ \alpha` = \mathbf{c/\ c`\ X\ Sin}\alpha$$

- In this equation *c*, *c*` & α are known, so we can calculate sin α` & by using the inverse trigonometric function of “ arcsin “ on any calculator we can accurately estimate the (new) AMA angle.

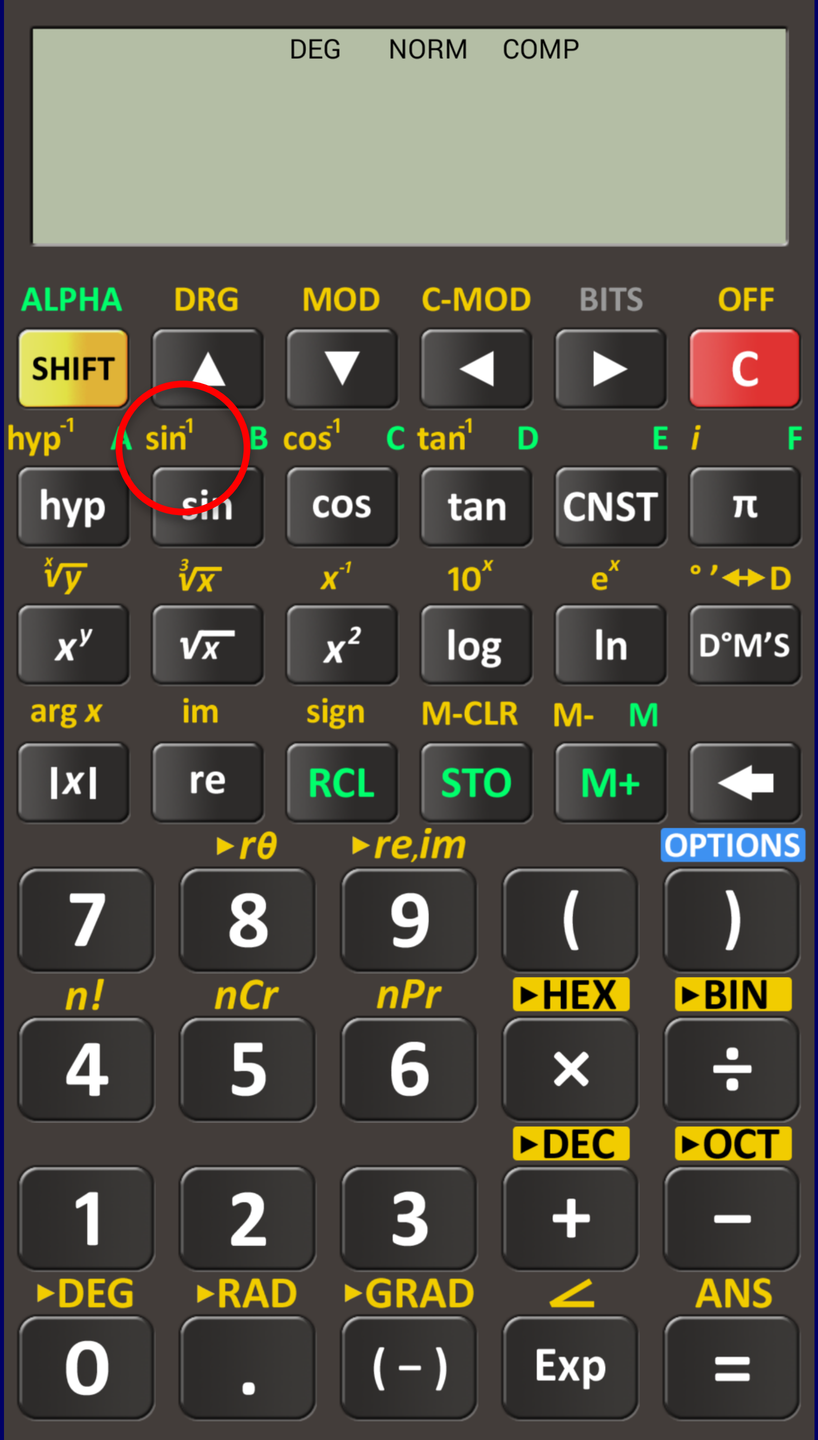
DEG NORM COMP
ALPHA DRG MOD C-MOD BITS OFF
SHIFT C
hyp-1 A sin-1 B cos-1 C tan-1 D E i F
hyp sin cos tan CNST π
x√y 3√x x-1 10x ex °'↔D
xy √x x2 log ln D°M'S
arg x im sign M-CLR M- M
|x| re RCL STO M+
►rθ ►re,im OPTIONS
7 8 9 ( )
n! nCr nPr ►HEX ►BIN
4 5 6 × ÷
►DEC ►OCT
1 2 3 + −
►DEG ►RAD ►GRAD ∠ ANS
0 . (−) Exp =

Take Home Message

This method is very similar to the classic CORA deformity analysis method, the only difference is:

When drawing the anatomic axis of the lower femoral fragment, instead of drawing it referenced to the mechanical axis at 7º, we use a smaller angle (which we got from the equation)

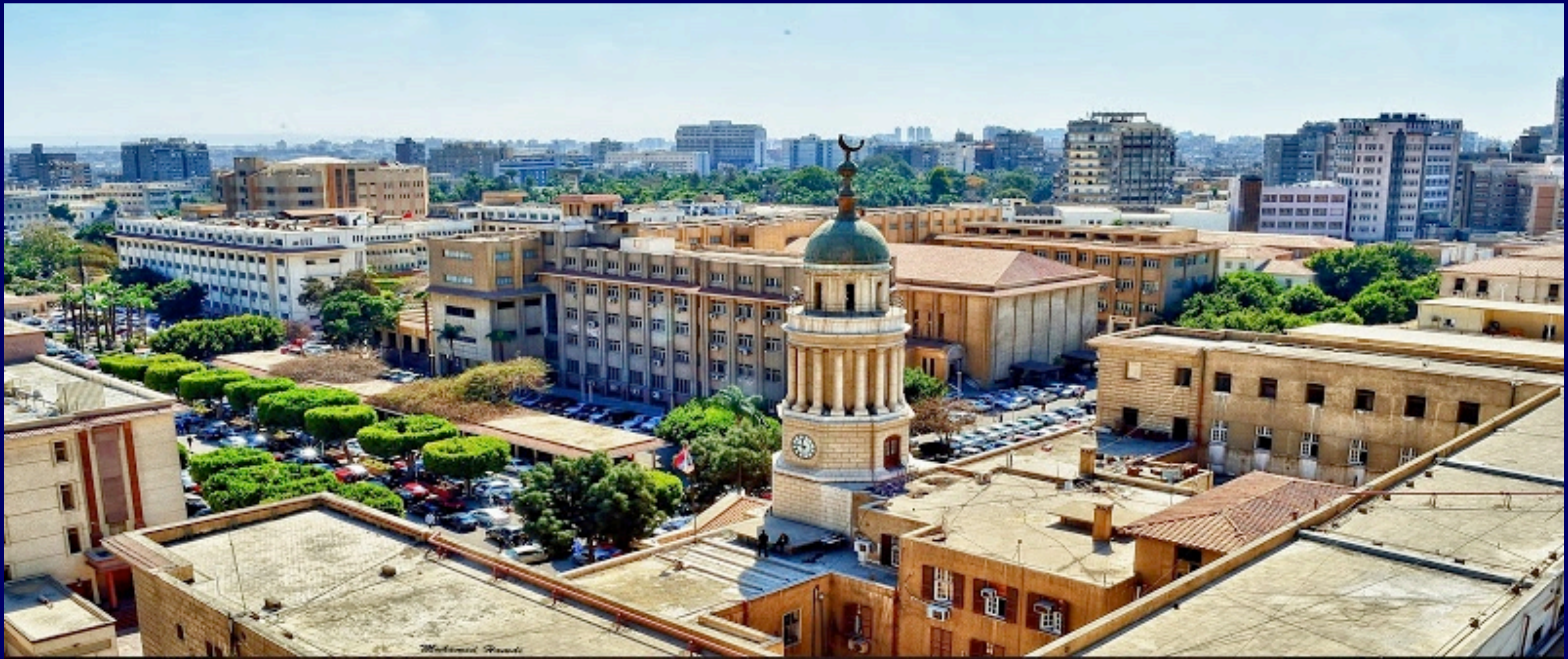

Thank you

Sherif.Galal@kasralainy.edu.eg

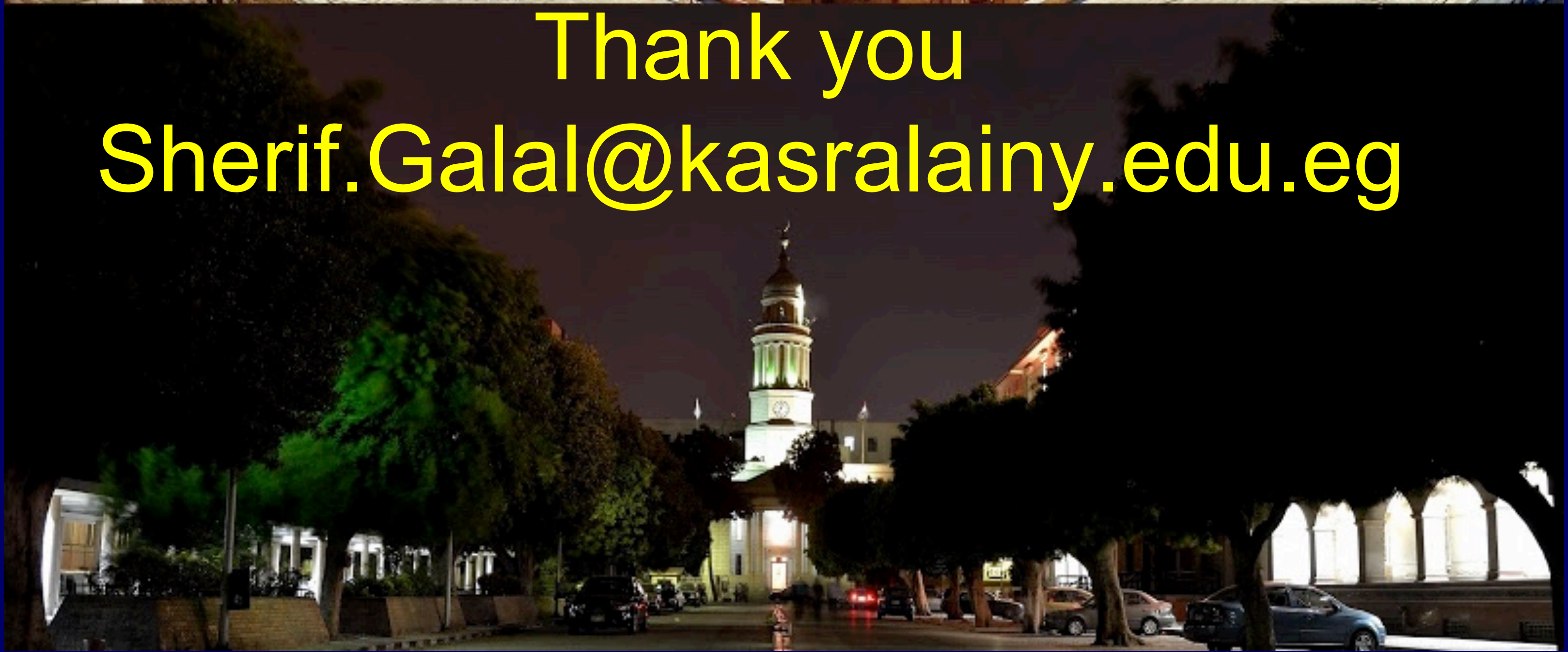

Supplement: Supplementary file 1 — Supplementary material 1 (PDF 29248 kb) [file 11751_2018_312_MOESM1_ESM.pdf]
